# Time trends in stroke incidence and modifiable risk factor prevalence in England (2018–2024): A record linkage population-based study

**DOI:** 10.1371/journal.pmed.1004999

**Published:** 2026-08-07

**Authors:** Joseph Kamtchum-Tatuene, James Farrell, John Nolan, Sarah Lessels, William N. Whiteley, Linxin Li, on behalf of the CVD-COVID-UK/COVID-IMPACT Consortium

**Affiliations:** 1 Wolfson Centre for Prevention of Stroke and Dementia, Nuffield Department of Clinical Neurosciences, John Radcliffe Hospital, University of Oxford, Oxford, United Kingdom; 2 British Heart Foundation Data Science Centre, Health Data Research UK, London, United Kingdom; 3 Centre for Clinical Brain Sciences, University of Edinburgh, Edinburgh, United Kingdom; Columbia University, UNITED STATES OF AMERICA

## Abstract

**Background:**

The incidence of stroke at younger ages appears to be increasing, whilst it is falling at older ages. We aimed to determine the current trend and how it might have changed during and after the COVID-19 pandemic.

**Methods and findings:**

We identified first-ever stroke events in adults in England between 1st April 2018 and 31st March 2024 through record-linkage with multiple overlapping sources including primary care, hospital admission, death certificate, and records from a stroke specific national audit. Standardised age-specific (age < 55 versus ≥ 55 years) stroke incidence was calculated and compared using incidence rate ratio (IRR) during three time periods (pre COVID-19 pandemic: 2018−2020; COVID-19 pandemic: 2020−2022 and post COVID-19 pandemic: 2022−2024). We quantified the age-specific divergence using the Relative Temporal Rate Ratio (RTTR). Diagnosis and treatment of modifiable stroke risk factors (hypertension, diabetes, atrial fibrillation, and hyperlipidaemia) before and after the stroke were also compared across the study periods. Analyses were stratified by sex, ethnicity, deprivation, regions, and stroke subtypes. 643,885 incident strokes were included (2018−2020: 206,290; 2020−2022: 211,420; and 2022−2024: 226,175). At age < 55 years, there was a significant increase of stroke incidence during the pandemic (2020−2022 versus 2018−2020: IRR = 1.07, 95%CI [1.05,1.09], *p* < 0.001), which was consistent by sex, ethnicity, deprivation, stroke subtype and region, and was most prominent for ethnic minority groups (Black: IRR = 1.13, 95%CI [1.05,1.21], *p* = 0.0005; Asian: IRR = 1.26, 95%CI [1.20,1.33], *p* < 0.001). This trend continued after the pandemic (2022−2024 vs. 2020−2022: IRR = 1.04, 95%CI [1.02,1.06], *p* < 0.001). On the contrary, stroke incidence at age ≥ 55 remained stable during the pandemic (IRR = 0.99, 95%CI [0.99,1.00], *p* = 0.06), and increased afterwards (IRR = 1.04, 95%CI [1.03,1.04], *p* < 0.001). Consequently, the age divergence in stroke incidence with a less favourable trend at younger ages widened significantly during the pandemic (RTTR = 1.07, 95%CI [1.05,1.08], *p* < 0.001) and remained unchanged since (RTTR = 0.99, 95%CI [0.97,1.00], *p* = 0.11). Prevalence of modifiable stroke risk factors remained largely unchanged during and after the pandemic for both age groups. However, there was a significant increase in the frequency of undiagnosed premorbid hypertension and diabetes at age < 55 years during the pandemic (hypertension RR = 1.07, 95%CI [1.02–1.13]; diabetes RR = 1.22, 95%CI [1.08–1.37]). Again, this trend was most prominent for ethnic minority groups and has not improved after the pandemic. A key limitation was the use of routinely collected data, which lacked granularity to explore potential mechanisms.

**Conclusions:**

There was an increase in stroke incidence at younger ages, with no increase at older ages, especially for ethnic minority groups, during and after the COVID-19 pandemic. This coincided with worsening in risk factor identification and diagnosis in primary care, highlighting missed opportunities that need to be urgently addressed.

## Author summary

**Why was this study done?:**

- Stroke incidence has declined at older ages but has approximately doubled among adults younger than 55 years in the UK over the past decade, with similar trends in other high-income countries.
- The divergence in incidence by age predates the COVID-19 pandemic, and trends during and after the pandemic were poorly characterised.
- Understanding the current trend is crucial for public health and service planning.

**What did the researchers do and find?:**

- This study used nation-wide record-linkage data with multiple overlapping sources to assess the ongoing time trends in stroke incidence by age.
- In England, the pre-existing age-specific divergence in stroke incidence widened during the COVID-19 pandemic, with less favourable trends among adults younger than 55 years, particularly in ethnic minority groups, and showed no evidence of reversal after the pandemic.
- This period was associated with sustained reductions in cardiovascular risk factor screening and diagnosis in primary care.

**What do the findings mean?:**

- Monitoring of ongoing trends in stroke incidence by age remains important.
- The worsening in risk factor screening and diagnosis in primary care during and after the COVID-19 pandemic means that there are potentially missed opportunities that could be addressed.
- From a methodology point of view, compared with a single source alone, linkage with multiple sources can help to minimise bias related to changes in patient behaviour. This also highlights support for the ongoing effort, policy and initiatives from funding agencies to improve electronic data collection and integration between various data sources.
- The main limitation of this study is the use of routinely collected data, which lacked granularity to allow further exploration of potential mechanistic drivers behind the apparent trends.

## Introduction

Although stroke incidence has decreased by about 42% in high-income countries over the last four decades, this is predominantly driven by a decline at older ages [1–3]. In younger people (under 55 years), there has been a doubling in stroke incidence over the last 10 years in the UK, and a consistent age-specific divergence in all other high-income countries, with a less favourable trend at younger ages [2,4]. However, available data only looked time trends until 2018 and little is known about the ongoing trend, especially in more recent periods, during and after the COVID-19 pandemic.

There are several potential causes of different stroke incidence trends in younger and older people in more recent periods. First, SARS-CoV-2 infection is associated with short-term risk of stroke with some evidence of a stronger effect at younger ages [5]. Second, the adenovirus-based COVID-19 vaccines were associated with intracranial venous thrombosis at age < 70 years, an otherwise rare cause of stroke [6,7]. Third, particularly during the pandemic, the control of vascular risk has declined, with fewer face-to-ace primary care appointment and prescription of antihypertensive, lipid lowering drugs and anticoagulation for atrial fibrillation [8,9]. Whether these changes differed by age is unclear but the decreased use of insulin - needed mainly in younger people with type 1 diabetes, but not other anti-diabetic medications, suggests that the use of preventive treatment more generally might have differentially affected patients at younger versus older ages [8]. Fourth, rising obesity levels appeared to be worse for younger than older individuals during the pandemic [10]. Fifth, there is some evidence to suggest that the COVID-19 pandemic had a greater impact on ethnic minorities who tend to be younger [11,12]. Finally, the high COVID-19 related mortality at older ages might have masked diagnosis of stroke, especially for those living in nursing homes, This might have contributed to an apparent low incidence of stroke at older ages, particularly during the pandemic.

We aimed to determine the age-specific changes in stroke incidence since 2018, particularly if there was any impact related to the COVID-19 pandemic, and to assess whether changes in the management of modifiable risk factors coincided with age-specific divergence in stroke incidence in England.

## Methods

### Study design, population and data sources

We accessed data in the National Health Service (NHS) England Secure Data Environment, via the British Heart Foundation (BHF) Data Science Centre’s CVD-COVID-UK/COVID-IMPACT Consortium. Detailed methods used for data linkage have been reported previously [13]. Briefly, the NHS England’s Master Person Service was used to match multiple records from different data sources to a single unique identifier, the NHS number, with 99% accuracy [13]. Once matching is completed, the NHS number is replaced with a nonidentifying unique master key (or pseudo-identifier), which then enables linkage of people’s records between datasets [13]. We included any first stroke between 1st April 2018 and 31st March 2024 in primary care (GP Extraction Service Data for Pandemic Planning and Research dataset, GDPPR), hospital admissions (Hospital Episodes Statistics, HES) and death records (Office of National Statistics, ONS). In addition, we also included stroke specific national audit data from the Sentinel Stroke National Audit Programme (SSNAP). The inclusion criteria were age ≥ 18 years at the time of stroke onset and diagnosis of stroke from at least one of the four data sources listed above. Patients were excluded if they had stroke recorded before 1st April 2018 in any source in their lifetime, their sex was unknown or the linkage across data sources was not possible. Patients were also excluded if they were either men with codes for pregnancy or women with codes for prostate cancer. For all patients identified, we recorded the ascertainment source. Events identified from different sources were treated as identical if the recorded event dates differed by <30 days.

### Diagnosis

Stroke: We defined stroke in HES and ONS records with the International Classification of Diseases 10th revision (ICD 10), which included I60 (nontraumatic subarachnoid haemorrhage - SAH), I61 (nontraumatic intracerebral haemorrhage - ICH), I63 (cerebral infarction) and I64 (unknown stroke); and in GDPPR with the Systematised Nomenclature of Medicine Clinical Terms (SNOMED-CT). In SSNAP, the diagnosis of stroke and the detailed stroke subtypes (ischaemic stroke versus intracerebral haemorrhage) are entered directly with the relevant text as a dropdown list. Fatal stroke was defined as any stroke with date of death (of any cause) recorded within 30 days of the index stroke date. Stroke severity was measured with the National Institutes of Health Stroke Scale (NIHSS) recorded in SSNAP.

Demographics: Patient age at stroke onset, sex, ethnicity (self reported: White individuals; Black individuals; Asian individuals; Other, including Arab or multiple ethnicity), deprivation (in quintiles of the Index of Multiple Deprivation - IMD), and regions (East, East Midlands, London, North East, North West, South East, South West, West Midlands, Yorkshire; defined with ONS Nomenclature of Territorial Units for Statistics - NUTS1 regions) were also collected.

Vascular risk factors: We also collected data on the presence and date of diagnosis of hypertension, diabetes, atrial fibrillation and hyperlipidaemia using pre-defined diagnostic codes in primary care (SNOMED CT codes from GDPPR) and in hospital (ICD-10 codes from HES). Medications taken before or after the index stroke were also recorded. With references to the index event, risk factors were considered (i) prevalent (present before stroke): if the patient received the diagnosis or the relevant treatment before or within 30 days of the index stroke; (ii) incident (likely absent before stroke, true new diagnosis after the stroke): if the diagnosis was made beyond 30 days after the index stroke, (iii) missed (false new diagnosis): among those with prevalent risk factors, if the diagnosis was absent before the index event but made at the time of the index stroke or within 30 days thereafter, and (iv) treated: if a relevant treatment was initiated within the study period. We chose to include risk factors diagnosed very early on after the stroke (i.e., within 30 days) as “prevalent” as it is most likely that these risk factors were already present at the time of the stroke but were not diagnosed. An illustration of the definitions for the diagnoses is listed under Appendix A in S1 File.

### Statistical analysis

We divided the study duration into three periods (pre COVID-19 pandemic 01 April 2018–31 March 2020; COVID-19 pandemic: 01 April 2020–31 March 2022 and post COVID-19 pandemic: 01 April 2022–31 March 2024). The study periods were defined using complete financial years (April–March) to ensure consistency in annual comparisons and to minimise seasonal variation. The pandemic period commenced on 01 April 2020, immediately following the declaration of the COVID-19 pandemic by the World Health Organization in March 2020 and the implementation of major public health restrictions in the UK. The post-pandemic period began on 01 April 2022, reflecting the widespread relaxation of COVID-19 restrictions and the gradual resumption of routine healthcare services. Crude incidence (per 1,000 population/year) was calculated with 95% confidence intervals estimated assuming a Poisson distribution for each time period. The mid-study population structure for each period (by age, sex, ethnicity, region, and IMD) reported by ONS was used as the reference population at risk. All crude incidence were directly standardised to the 2011 population of England and Wales. Poisson regression models, adjusted for the population age (18–34, 35–44, 45–54, 54–64, 65–74, 75–84, ≥85) and sex structures, were used to calculate incidence rate ratios (IRRs) for 2020–2022 versus 2018–2020 and for 2022–2024 versus 2020–2022. We also added an additional post-hoc analysis reporting the overall and the age-stratified incidence for each financial year per reviewers’ request. To measure the age-specific divergence in IRR, the relative temporal difference (Relative Temporal Rate Ratio- RTTR) was calculated by dividing the IRR at younger than 55 years by the IRR at age 55 years or older (Appendix B in S1 File) [4].

Rates of prevalent, incident, missed or treated hypertension, diabetes mellitus, dyslipidaemia, and atrial fibrillation were computed in each of the three study periods and compared using a chi-squared test. We also computed rate ratio with their 95% confidence interval to quantify the trends across the study periods.

All analyses were stratified by age (<55 years versus ≥55 years), sex, and ethnicity (White, Black, Asian). Participants with missing ethnicity data (*n* = 3,244), classified as “mixed” (*n* = 4,957), or “other” (*n* = 6,797) ethnic groups were not included in analyses stratified by ethnicity. For stroke incidence, we also performed further stratifications by deprivation, regions, stroke subtypes (ischaemic stroke; ICH; SAH; unknown strokes), and ascertainment methods (GDPPR versus HES versus SSNAP).

As per SDE requirement, all counts were rounded to the nearest 5 for reporting. All analyses were performed using Stata 17.0. The study protocol, all source code and the codelist used to define diagnoses and patient demographics are available at https://github.com/BHFDSC/CCU084_01 (https://doi.org/10.5281/zenodo.21672304).

### Reporting

This study is reported as per the Reporting of Studies Conducted using Observational Routinely-Collected Data (RECORD) guideline (S1 Checklist).

AI tools had no role and were not used in any form during the conduct of the study. The corresponding author retains responsibility for the final manuscript.

### Ethical approval

The North East - Newcastle and North Tyneside 2 research ethics committee provided ethical approval for the CVD-COVID-UK/COVID-IMPACT research programme (REC no. 20/NE/0161) to access, within secure trusted research environments, unconsented, whole-population, anonymised data from electronic health records collected as part of patients’ routine healthcare.

## Results

There were 643,885 incident strokes between 2018 and 2024 with 206,290, 211,420, and 226,175 events in 2018–2020, 2020–2022 and 2022–2024 (Appendix C in S1 File).

Table 1 shows the crude incidence by age. The overall age standardised incidence was 2.17 (95% [2.16,2.18]) per 1,000 population in 2018–2020, which remained largely the same in 2020–2022 during the pandemic (IRR = 1.00, 95%CI [1.00,1.01], *p* = 0.14; Table 1) and increased in 2022–2024 (IRR = 1.04, 95%CI [1.03,1.04], *p* < 0.001; Table 1). Results were consistent in analyses by each financial year (Appendix D in S1 File). These overall trends were consistent by sex, deprivation, region and were also seen for ischaemic stroke, ICH and unknown stroke. Age standardised incidence for SAH was stable between 2020–2022 versus 2018–2020 and declined by 4% in 2022–2024 (Appendix E in S1 File).

**Table 1:** Age-standardised stroke incidence (per 1,000 person-years) in England between 2018 and 2024.

	2018–2020:			2020–2022:			2022–2024:		
Age	No. stroke	No. at risk	Rate (95%CI)	No. stroke	No. at risk	Rate (95%CI)	No. stroke	No. at risk	Rate (95%CI)
18–34	3,185	24,074,387	0.13 (0.13, 0.14)	3,395	24,731,274	0.14 (0.13, 0.14)	3,585	25,192,810	0.14 (0.14, 0.15)
35–44	6,200	14,309,229	0.43 (0.42, 0.44)	6,880	14,778,767	0.47 (0.45, 0.48)	7,475	15,468,604	0.48 (0.47, 0.49)
45–54	17,275	15,237,615	1.13 (1.12, 1.15)	17,975	14,908,108	1.21 (1.19, 1.22)	18,395	14,486,580	1.27 (1.25, 1.29)
55–64	30,025	13,570,635	2.21 (2.19, 2.24)	32,820	14,199,736	2.31 (2.29, 2.34)	35,415	14,669,186	2.41 (2.39, 2.44)
65–74	45,985	11,148,977	4.12 (4.09, 4.16)	46,645	11,108,571	4.20 (4.16, 4.24)	48,320	10,945,730	4.41 (4.38, 4.45)
75–84	59,060	6,743,631	8.76 (8.69, 8.83)	59,730	7,097,957	8.42 (8.35, 8.48)	66,255	7,727,220	8.57 (8.51, 8.64)
85+	44,560	2,782,745	16.01 (15.86, 16.16)	43,980	2,809,404	15.65 (15.51, 15.80)	46,730	2,893,224	16.15 (16.01, 16.30)
**Total**	**206,290**	**87,867,217**	**2.35 (2.34, 2.36)**	**211,425**	**89,633,815**	**2.36 (2.35, 2.37)**	**226,175**	**91,383,354**	**2.48 (2.46, 2.49)**
		Standardised	**2.17 (2.16–2.18)**		Standardised	**2.18 (2.17–2.19)**		Standardised	**2.26 (2.25–2.27)**
**IRR (95%CI)**					2020-2022 vs. 2018–2020:			2024−2022 vs. 2022−2020:	
					1.00 (1.00–1.01) *p* = 0.18			1.04 (1.03–1.04) *p* < 0.001	

Poisson regression models, standardised for the population age (18–34, 35–44, 45–54, 54–64, 65–74, 75–84, ≥ 85), were used to calculate incidence rate ratios (IRRs). Data were standardised to the 2011 census population of England and Wales.

### The trends, however, differed by age and by ethnic group

At ages 55 and over, stroke incidence was similar in 2018–2020 and 2020–2022 (IRR = 0.99, 95%CI [0.99,1.00], *p* = 0.06; Appendix E in S1 File), largely consistent by sex, deprivation, stroke subtype, most of the regions and White ethnicity (Table 2). Incidence was higher in 2020–2022 for people with Black ethnicity (IRR = 1.08, 95%CI [1.03,1.13], *p* = 0.001; Table 2), mostly in Black African (IRR = 1.13, 95%CI [1.03,1.23] versus Black Caribbean IRR = 1.02, 95%CI [0.96,1.09]; Appendix F in S1 File), and for people with Asian ethnicity(IRR = 1.04, 95%CI [1.00,1.07], *p* = 0.02; Table 2), mostly in Asian Bangladeshi (IRR = 1.09, 95%CI [0.98,1.22]; Indian IRR = 1.00, 95%CI [0.96,1.05]; Pakistani IRR = 0.99, 95%CI [0.93,1.05]; Appendix F in S1 File).

**Table 2:** Time trends of age-standardised stroke incidence (per 1,000 person-years) in England between 2018 and 2024 stratified by sex, ethnicity, deprivation, stroke subtypes and region in those aged ≥55 years. (ASI = Absolute stroke incidence) Poisson regression models, standardised for the population age (18–34, 35–44, 45–54, 54–64, 65–74, 75–84, ≥85), were used to calculate incidence rate ratios (IRRs). Data were standardised to the 2011 census population of England and Wales.

	2018–2020	2020–2022	2022–2024	2020–2022 vs. 2018–2020		2022–2024 vs. 2020–2022	
	ASI (95%CI)	ASI (95%CI)	ASI (95%CI)	IRR (95%CI)	*p*	IRR (95%CI)	*p*
**Sex**							
Male	5.79 (5.75, 5.83)	5.80 (5.76, 5.84)	6.03 (5.99, 6.07)	1.00 (0.99, 1.01)	0.71	1.04 (1.03, 1.05)	<0.001
Female	4.62 (4.59, 4.65)	4.55 (4.52, 4.58)	4.69 (4.66, 4.72)	0.98 (0.97, 0.99)	0.001	1.03 (1.02, 1.04)	<0.001
**Ethnicity**							
White	5.12 (5.10, 5.14)	5.07 (5.05, 5.09)	5.21 (5.19, 5.23)	0.99 (0.98, 1.00)	0.004	1.03 (1.02, 1.04)	<0.001
Black	5.75 (5.55, 5.95)	6.21 (6.01, 6.41)	7.07 (6.86, 7.28)	1.08 (1.03, 1.13)	0.001	1.14 (1.09, 1.19)	<0.001
Asian	5.61 (5.48, 5.74)	5.82 (5.69, 5.95)	6.44 (6.31, 6.57)	1.04 (1.00, 1.07)	0.02	1.11 (1.07, 1.18)	<0.001
**Deprivation**							
Quintile 1 (most deprived)	6.45 (6.38, 6.52)	6.31 (6.24, 6.38)	6.49 (6.42, 6.56)	0.98 (0.96, 0.99)	0.005	1.03 (1.01, 1.04)	<0.001
Quintile 2	5.59 (5.53, 5.65)	5.60 (5.54, 5.66)	5.71 (5.65, 5.77)	1.00 (0.99, 1.02)	0.81	1.02 (1.00, 1.03)	0.009
Quintile 3	5.14 (5.09, 5.20)	5.07 (5.02, 5.12)	5.21 (5.16, 5.26)	0.99 (0.97, 1.00)	0.06	1.03 (1.01, 1.04)	<0.001
Quintile 4	4.75 (4.70, 4.80)	4.79 (4.74, 4.84)	5.01 (4.96, 5.06)	1.01 (0.99, 1.02)	0.25	1.05 (1.03, 1.06)	<0.001
Quintile 5 (least deprived)	4.49 (4.44, 4.54)	4.44 (4.39, 4.49)	4.67 (4.62, 4.72)	0.99 (0.97, 1.00)	0.13	1.05 (1.04, 1.07)	<0.001
**Subtype**							
Ischaemic stroke	3.75 (3.73, 3.77)	3.73 (3.71, 3.75)	3.87 (3.85, 3.89)	0.99 (0.98, 1.00)	0.17	1.04 (1.03, 1.05)	<0.001
Intracerebral haemorrhage	0.59 (0.58, 0.60)	0.58 (0.57, 0.59)	0.60 (0.59, 0.61)	0.98 (0.96, 1.00)	0.08	1.03 (1.02, 1.05)	<0.001
Subarachnoid haemorrhage	0.15 (0.14, 0.15)	0.15 (0.14, 0.15)	0.14 (0.14, 0.14)	1.01 (0.97, 1.05)	0.73	0.96 (0.92, 1.00)	0.03
Unknown stroke	0.71 (0.70, 0.72)	0.70 (0.69, 0.70)	0.73 (0.72, 0.74)	0.98 (0.96, 1.00)	0.04	1.05 (1.03, 1.07)	<0.001
**Region**							
East	5.03 (4.96, 5.10)	4.92 (4.85, 4.99)	5.05 (4.98, 5.12)	0.98 (0.96, 1.00)	0.02	1.03 (1.01, 1.05)	0.009
East Midlands	5.38 (5.29, 5.46)	5.40 (5.32, 5.48)	5.56 (5.48, 5.64)	1.01 (0.98, 1.03)	0.65	1.03 (1.01, 1.05)	0.008
London	5.54 (5.46, 5.62)	5.37 (5.29, 5.45)	5.65 (5.57, 5.73)	0.97 (0.95, 0.99)	0.003	1.05 (1.03, 1.07)	<0.001
North East	6.22 (6.10, 6.34)	5.99 (5.88, 6.11)	6.34 (6.22, 6.46)	0.96 (0.94, 0.99)	0.006	1.06 (1.03, 1.09)	<0.001
North West	5.83 (5.76, 5.90)	5.73 (5.66, 5.80)	6.00 (5.93, 6.07)	0.98 (0.97, 1.00)	0.07	1.05 (1.03, 1.06)	<0.001
South East	5.16 (5.10, 5.22)	5.18 (5.12, 5.24)	5.19 (5.13, 5.25)	1.00 (0.99, 1.02)	0.67	1.00 (0.99, 1.02)	0.83
South West	5.53 (5.46, 5.60)	5.34 (5.27, 5.41)	5.50 (5.43, 5.57)	0.97 (0.95, 0.98)	0.0003	1.03 (1.01, 1.05)	0.002
West Midlands	5.27 (5.20, 5.35)	5.18 (5.11, 5.25)	5.26 (5.19, 5.33)	0.98 (0.96, 1.00)	0.09	1.02 (1.00, 1.04)	0.13
Yorkshire	5.73 (5.65, 5.81)	5.74 (5.66, 5.82)	6.08 (6.00, 6.16)	1.00 (0.98, 1.02)	0.87	1.06 (1.04, 1.08)	<0.001

At ages 55 and under, stroke incidence was higher in 2020–2022 than 2018–2020 (IRR = 1.07, 95%CI [1.05,1.09], *p* < 0.001), consistent by sex, ethnicity, deprivation, stroke subtype, and region (Table 3). The increase was marked for ethnic minority groups (Black ethnicity: IRR = 1.13, 95%CI [1.05,1.21], *p* = 0.0005; Asian ethnicity: IRR = 1.26, 95%CI [1.20,1.33], *p* < 0.001; Table 3) and was consistent with further stratification within the Asian ethnicity group (Appendix F in S1 File).

**Table 3:** Time trends of age-standardised stroke incidence (per 1,000 person-years) in England between 2018 and 2024 stratified by sex, ethnicity, deprivation, stroke subtypes and region in those aged < 55 years. (ASI = Absolute stroke incidence) Poisson regression models, standardised for the population age (18–34, 35–44, 45–54, 54–64, 65-74–, 75-–84, ≥85), were used to calculate incidence rate ratios (IRRs). Data were standardised to the 2011 census population of England and Wales.

	2018–2020	2020–2022	2022–2024	2020–2022 vs. 2018–2020		2022–2024 vs. 2020–2022	
	ASI (95%CI)	ASI (95%CI)	ASI (95%CI)	IRR (95%CI)	*p*	IRR (95%CI)	*p*
**Sex**							
Male	0.57 (0.56, 0.58)	0.62 (0.61, 0.63)	0.65 (0.64, 0.66)	1.09 (1.06, 1.11)	<0.001	1.05 (1.03, 1.07)	<0.001
Female	0.40 (0.39, 0.41)	0.42 (0.41, 0.43)	0.44 (0.43, 0.45)	1.05 (1.02, 1.08)	0.0003	1.05 (1.02, 1.07)	<0.001
**Ethnicity**							
White	0.49 (0.48, 0.50)	0.51 (0.50, 0.52)	0.52 (0.51, 0.53)	1.04 (1.02, 1.06)	<0.001	1.02 (1.00, 1.04)	0.04
Black	0.63 (0.60, 0.66)	0.71 (0.68, 0.74)	0.80 (0.77, 0.83)	1.13 (1.05, 1.21)	0.0005	1.13 (1.06, 1.20)	0.0002
Asian	0.42 (0.40, 0.44)	0.53 (0.51, 0.55)	0.60 (0.58, 0.62)	1.26 (1.20, 1.33)	<0.001	1.13 (1.08, 1.19)	<0.001
**Deprivation**							
Quintile 1 (most deprived)	0.72 (0.70, 0.74)	0.77 (0.75, 0.79)	0.81 (0.79, 0.83)	1.07 (1.04, 1.10)	<0.001	1.05 (1.02, 1.08)	0.001
Quintile 2	0.54 (0.53, 0.55)	0.58 (0.57, 0.59)	0.59 (0.58, 0.60)	1.07 (1.04, 1.11)	<0.001	1.02 (0.98, 1.05)	0.33
Quintile 3	0.44 (0.43, 0.45)	0.47 (0.45, 0.48)	0.49 (0.48, 0.50)	1.06 (1.02, 1.11)	0.002	1.05 (1.01, 1.09)	0.01
Quintile 4	0.38 (0.37, 0.39)	0.40 (0.39, 0.41)	0.43 (0.42, 0.44)	1.05 (1.01, 1.10)	0.02	1.08 (1.03, 1.12)	0.0006
Quintile 5 (least deprived)	0.33 (0.32, 0.34)	0.35 (0.34, 0.36)	0.36 (0.35, 0.37)	1.06 (1.01, 1.11)	0.01	1.03 (0.98, 1.08)	0.22
**Subtype**							
Ischaemic stroke	0.28 (0.28, 0.28)	0.31 (0.31, 0.31)	0.32 (0.32, 0.32)	1.11 (1.08, 1.13)	<0.001	1.03 (1.01, 1.05)	0.003
Intracerebral haemorrhage	0.07 (0.07, 0.07)	0.07 (0.07, 0.07)	0.08 (0.08, 0.08)	1.09 (1.04, 1.14)	0.0001	1.07 (1.02, 1.12)	0.002
Subarachnoid haemorrhage	0.06 (0.05, 0.06)	0.06 (0.06, 0.06)	0.05 (0.05, 0.05)	1.03 (0.98, 1.09)	0.19	0.92 (0.88, 0.97)	0.002
Unknown stroke	0.08 (0.08, 0.08)	0.08 (0.08, 0.08)	0.09 (0.09, 0.09)	1.02 (0.98, 1.07)	0.30	1.11 (1.06, 1.15)	<0.001
**Region**							
East	0.42 (0.41, 0.44)	0.45 (0.43, 0.47)	0.49 (0.47, 0.51)	1.07 (1.01, 1.12)	0.02	1.09 (1.04, 1.15)	0.0008
East Midlands	0.48 (0.46, 0.50)	0.53 (0.51, 0.55)	0.56 (0.54, 0.58)	1.12 (1.06, 1.18)	<0.001	1.05 (1.00, 1.11)	0.07
London	0.46 (0.45, 0.48)	0.49 (0.48, 0.51)	0.51 (0.50, 0.52)	1.07 (1.03, 1.11)	0.002	1.03 (0.99, 1.15)	<0.001
North East	0.59 (0.56, 0.62)	0.63 (0.60, 0.66)	0.66 (0.63, 0.69)	1.07 (0.99, 1.14)	0.08	1.05 (0.98, 1.13)	0.15
North West	0.54 (0.52, 0.56)	0.58 (0.56, 0.60)	0.59 (0.57, 0.61)	1.07 (1.03, 1.12)	0.002	1.02 (0.98, 1.06)	0.39
South East	0.40 (0.39, 0.42)	0.44 (0.42, 0.45)	0.45 (0.44, 0.46)	1.08 (1.03, 1.13)	0.0007	1.03 (0.99, 1.08)	0.12
South West	0.43 (0.42, 0.45)	0.46 (0.44, 0.48)	0.51 (0.49, 0.53)	1.06 (1.00, 1.12)	0.06	1.12 (1.06, 1.18)	<0.001
West Midlands	0.48 (0.46, 0.50)	0.50 (0.48, 0.52)	0.52 (0.50, 0.54)	1.05 (0.99, 1.10)	0.08	1.03 (0.98, 1.09)	0.20
Yorkshire	0.53 (0.51, 0.55)	0.57 (0.55, 0.59)	0.64 (0.62, 0.66)	1.09 (1.04, 1.15)	0.0008	1.11 (1.06, 1.17)	<0.001

**Fig 1:**
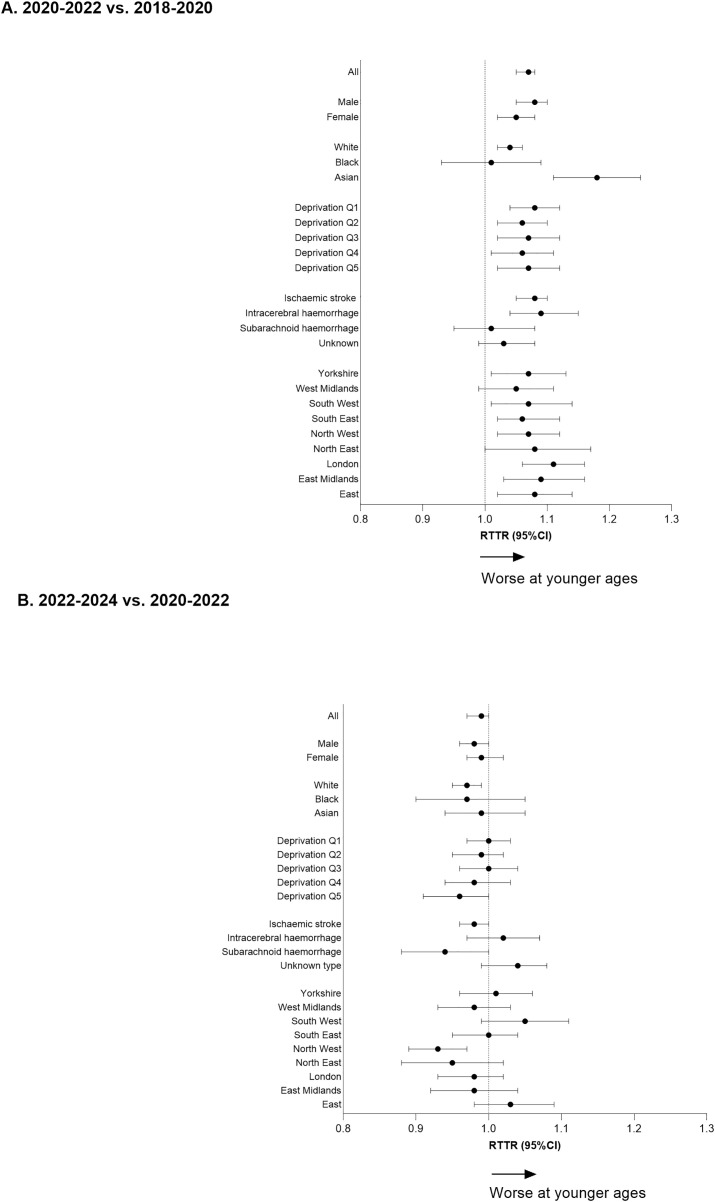
Age-specific divergence in stroke incidence in England between 2018 and 2024. Each line represents the RTTR with 95%CI for a specified strata. **A.** Comparisons between 2020–2022 and 2018–2020; **B.** Comparisons between 2022–2024 vs. 2020–2022. Details of the calculation and interpretation of the relative temporal trend ratio (RTTR) are presented in Appendix B in S1 File. RTTR = relative temporal trend ratio, RTTR>1 indicates a less favourable trend at younger ages.

Consequently, the age divergence in stroke incidence with a less favourable trend at younger ages was greater in 2020–2022 compared to 2018–2020 (incidence at younger versus older ages between the two time periods - RTTR = 1.07, 95%CI [1.05,1.08], *p* < 0.001; Fig 1A) and was consistent for all subgroups except for individuals with Black ethnicity (RTTR = 1.01, 95%CI [0.93,1.09], *p* = 0.86; Fig 1A).

From 2022–2024 to 2020–2022, stroke incidence was higher, at both younger and at older ages (Tables 2, 3 and Appendix E in S1 File; RTTR 0.99, 95%CI [0.97,1.00], *p* = 0.11) with little important difference by sex, ethnicity, deprivation, stroke subtype and region (Fig 1B).

Results stratified by age and ascertainment methods are presented in Appendix G in S1 File. Despite the differences in the absolute incidence and changes in incidence by ascertainment, the age-specific divergence with a less favourable trend at younger ages was largely consistent for HES and SSNAP between 2018–2022 and 2020–2022 (HES: RTTR = 1.08, 95%CI [1.06,1.11]; SSNAP: RTTR = 1.13, 95%CI [1.10,1.16]), but not in GDPPR alone (RTTR = 0.98, 95%CI [0.96,1.00]). (Appendix G in S1 File). These also stabilised between 2022–2024 and 2020–2022 (GDPPR: RTTR = 1.00, 95%CI [0.98,1.02]; HES: RTTR = 0.98, 95%CI [0.96,1.00]; SSNAP: RTTR = 0.99, 95%CI [0.97,1.02]).

The proportion of strokes that were fatal was largely similar in 2018–2020 (11.8%), 2020–2022 (11.9%), and 2022–2024 (10.9%), consistent between subtypes (Appendix H in S1 File). Where severity was measured with NIHSS, stroke tended to be less severe in 2022–2024 versus 2020–2022 versus 2018–2020, which was consistent by age and ethnicity (Appendix I and J in S1 File).

**Fig 2:**
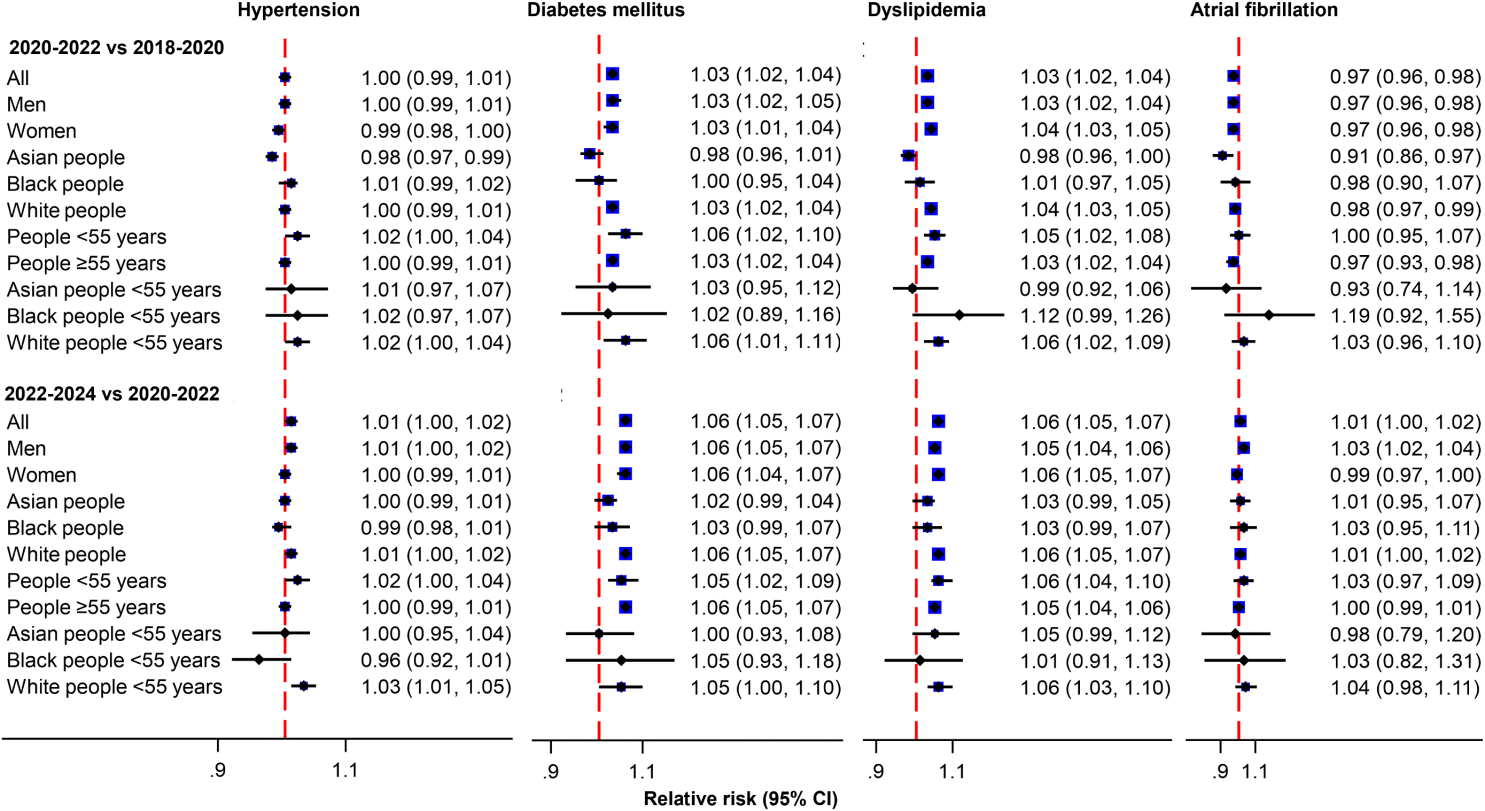
Time trends of prevalent vascular risk factors in England between 2018 and 2024. Each blue diamond represents the relative risk of hypertension, diabetes, dyslipidaemia, or atrial fibrillation within the relevant population subgroup. The horizontal tip on each side of the blue diamond represents the 95% CI of the relative risk. The vertical dashed red line represents the line of no difference (risk ratio = 1) between the periods compared (2020–2022 vs. 2018–2020 or 2022–2024 vs. 2020–2022). Risk ratio (RR)>1 means that the risk factor of interest is more common in the period listed first (i.e., 2020–2022 in the plots of the top row and 2022–2024 in the plots of the bottom row). RR < 1 means that the risk factor of interest is less common in the period listed first (i.e., 2020–2022 in the plots of the top row and 2022–2024 in the plots of the bottom row).

**Fig 3:**
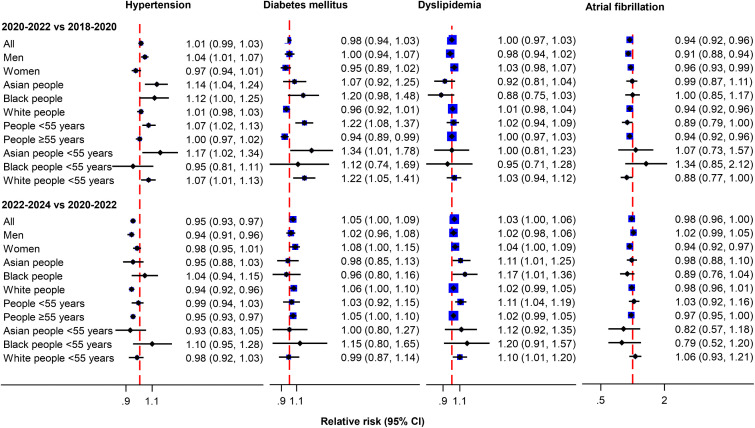
Time trends of vascular risk factors that were missed in primary care before the index stroke event in England between 2018 and 2024. Each blue diamond represents the relative risk of hypertension, diabetes, dyslipidaemia, or atrial fibrillation within the relevant population subgroup. The horizontal tip on each side of the blue diamond represents the 95% CI of the relative risk. The vertical dashed red line represents the line of no difference (odds ratio = 1) between the periods compared (2020–2022 vs. 2018–2020 or 2022–2024 vs. 2020–2022).

For risk factors, the prevalence of known and treated hypertension was similar in 2018–2020 and 2020–2022, and higher in 2022–2024 versus 2020–2022 (Appendix K in S1 File), consistent by sex (Appendix L and M in S1 File), at age ≥ 55 years (Figs 2 and 3 and Appendix N in S1 File) and for White (Appendix O in S1 File). At age < 55 years, there was a small increase in prevalent and treated hypertension over three study periods (Appendix P in S1 File). Moreover, at age < 55 years, undiagnosed premorbid hypertension was also higher during the pandemic (10.8% versus 11.6%; RR = 1.07, 95%CI [1.02,1.13], *p* = 0.003; Fig 3 and A*pp*endix P in S1 File), with no change since (RR = 0.99, 95%CI [0.94,1.03], *p* = 0.57; Fig 3 and A*pp*endix P in S1 File). Furthermore, compared with older patients, those aged < 55 years with known hypertension were less likely treated prior to stroke, similarly over time (age </≥55 years – 2018–2020 42.2% versus 48.6%; 2020–2022 42.1% versus 49.0%; 2022–2024 44.7% versus 49.9%; all *p* < 0.0001; A*pp*endix N and P in S1 File).

For individuals with Black and Asian ethnicity, prevalences of known and treated hypertension remained largely stable during the three study periods (Fig 2 and Appendix Q-R in S1 File). However, the significant increase in the frequency of undiagnosed hypertension was also seen (Asian RR = 1.14, 95%CI [1.04,1.24]; Black RR = 1.12. 95%CI [1.00,1.25]; Fig 3 and Appendix Q–R in S1 File), which again remained consistent since the pandemic (Asian RR = 0.95, 95%CI [0.99,1.03]; Black RR = 1.04, 95%CI [0.98,1.15]; Appendix Q-R in S1 File) and was most prominent for individuals with Asian ethnicity at age < 55 years (RR = 1.17, 95%CI [1.02,1.34]; Appendix S-U in S1 File). It is also worth noting that compared to individuals with White and Asian ethnicity, those with Black ethnicity was less often prescribed any blood pressure lowering drug despite known history of hypertension, which again remained unchanged during the three study periods (2018–2020: Black ethnicity 44.1% versus White ethnicity 47.9% versus Asian ethnicity 54.6%; 2020–2022: Black ethnicity 45.2% versus White ethnicity 48.2% versus Asian ethnicity 54.1%; 2022–2024: Black ethnicity 45.0% versus White ethnicity 49.4% versus Asian ethnicity 55.7%; all *p* < 0.0001; Appendix V in S1 File).

Overall, diabetes and hyperlipidaemia had higher prevalence from 2018–2020 to 2020–2022 to 2022–2024 (Appendix K), consistent by age and sex (Appendix L and M, N and P in S1 File). This increase was true only for patients with White ethnicity (Appendix O in S1 File), while patients with Asian (Appendix Q in S1 File) and Black ethnicity (Appendix R in S1 File) had fewer diagnoses between 2018–2020 and2020–2022, with an increase in 2022–2024. There was more undiagnosed diabetes during the pandemic at age < 55 years (2.1% versus 1.8%; RR = 1.22, 95%CI [1.08,1.37]; Fig 3 and Appendix P in S1 File) and for patients with Asian and Black ethnicity at any age (Asian RR = 1.07, 95%CI [0.92,1.25]; Black RR = 1.20, 95%CI [0.98,1.48]; Fig 3 and Appendix Q–R in S1 File), most prominent for Asian people <55 years (RR = 1.34, 95%CI [1.01,1.78]; Appendix S–U in S1 File), which remained unchanged since the pandemic (Fig 3 and Appendix P–R in S1 File).

Similar to hypertension, there were differences in initiation of treatment by age and ethnicity in patients with known diabetes and hyperlipidaemia. Compared with patients ≥55 years, patients <55 years with known hyperlipidaemia were less likely to be treated with lipid lowering drugs throughout the three study periods (age </≥55 years: 2018–2020 74.3% versus 81.1%; 2020–2022 75.4% versus 82.4%; 2022–2024 75.8% versus 83.2%; all *p* < 0.0001; Appendix N and P in S1 File), although there was no difference in initiation of diabetic-related treatment in those with diabetes (age </≥55 years: 2018–2020 62.7% versus 61.3%, *p* = 0.15; 2020–2022 62.6% versus 62.4%, *p* = 0.78; 2022–2024 65.5% versus 64.3%, *p* = 0.78; Appendix N and P in S1 File). For ethnic groups, compared to patients with White and Asian ethnicity, patients with Black ethnicity were again less often prescribed lipid lowering or diabetic treatment despite known history of diabetes and hyperlipidaemia, which remained unchanged during the three study periods. The ethnic differences were most prominent at age < 55 years (Appendix V in S1 File).

There was an overall decrease in incident and prevalent atrial fibrillation from 2018–2020 to 2020–2022, followed by an increase in 2022–2024. This overall trend was consistent in analyses stratified by age, sex, and ethnic group (web appendices K-R).

## Discussion

In this nationwide study in England, we used multiple overlapping data sources to identify all patients with incident stroke. Stroke incidence at age < 55 years increased between 2018 and 2024. Consequently, the age divergence in stroke incidence, with greater incidence at younger ages, widened during the COVID-19 pandemic, especially for ethnic minority groups. This has not reversed since the pandemic ended in 2022. These changes in incidence coincided with sub-optimal risk factor management, screening and diagnosis throughout the study periods, which was most prominent at younger ages and for ethnic minority groups.

Our data adds to existing literature on time trends in stroke incidence by age. We previously showed that stroke incidence at age < 55 years almost doubled between 2002 and 2018 in Oxfordshire, UK [2]. The absolute increase of ~10% between 2018 and 2024 in the current study may appear to be slower than expected but might still be an underestimation due to possible under-ascertainment between 2020 and 2022 as younger patients with less severe symptoms were less likely to seek medical attention for stroke symptoms during the pandemic [14].

There are probably two drivers for the higher incidence at younger ages. First, the sub-optimal diagnosis and management of vascular risk factors. There was a significant increase in the frequency of undiagnosed premorbid hypertension and diabetes at age < 55 years at stroke diagnosis during the pandemic, which has not improved since. This is supported by the national health survey in England; a national study on medicine prescription in primary care; and a fall in blood pressure recording in London, particularly at younger ages [15–17]. This reduction could be due to substitution of face-to-face consultation by virtual clinics, particularly for younger people and sub-optimal risk factor management during hospital admissions [18]. Research into the best method to reduce missed opportunities for primary prevention is needed, especially for younger patients. This is particularly relevant since the latest guidelines recommend treatment of vascular risk factors based on level of measurements rather than purely based on the predicted 10-year risk, which is often low at younger ages [19–21]. Second, there is likely further contribution from unknown factors that were seen pre-pandemic [2,4]. Changes in patient behaviour or investigations are unlikely to be the main drivers and some synergistic effect of traditional vascular risk factors, such as hypertension, diabetes and obesity, and emerging stroke risk factors, such as stress and pollution, could likely be playing a role [2].

In contrast with the previously reported decline [1,3], we found that stroke incidence at older ages remained stable during the COVID-19 pandemic and started to increase since the end of the pandemic. This is in line with recent data from the Global Burden of Disease Study, which also showed that whilst there was a decline of stroke incidence between 1990 and 2019, the burden of stroke exhibited a stable trajectory from 2019 to 2021 [22]. As we did not have data before 2018, the exact turning point is difficult to assess. A previous UK-wide study showed that stroke incidence declined by 25% between 2000 and 2019 [3], suggesting that the pandemic which started in early 2020 most likely contributed to the stalling and increase we observed between 2020 and 2024. Moreover, as patients were less likely to present to medical attention and hence less likely to be diagnosed during the peak of the pandemic [14], the apparent stabilisation of stroke incidence during this period could be an underestimation of a true increase. This possible reversal in trend at older ages share some of the mechanistic drivers discussed above for strokes at younger ages, such as the long-term impact related to the SARS-CoV-2 infection and the worsening of risk factor control in primary prevention more generally [6,8]. However, unlike at younger ages, we did not see any significant increase of under-diagnosis of stroke risk factors during the study periods at age ≥ 55 years. This was also true for atrial fibrillation, which is more common at older ages. Nevertheless, the proportions of patients on treatment for known risk factors were sub-optimal and remained unchanged between 2020 and 2024. Therefore, improvement in risk factor management is likely to translate into better stroke prevention and close monitoring of the ongoing trend will be crucial.

There were ethnic disparities in stroke risk and in management of stroke risk factors. Compared with people with White ethnicity, we found higher stroke incidence for people with Black (particularly African Black) and Asian ethnicity, which increased from 2018 to 2024, at all ages. Moreover, diagnosis and treatment for known stroke risk factors were worse for minority groups, particularly hypertension and diabetes, although whether these observed unfavourable trends by ethnicity in England were an acceleration or a reversal of trends observed before the pandemic is yet to be determined [23–29]. This suggests that whilst recovery of management of vascular risk factors, irrespective of ethnicity is still ongoing, targeted intervention might be needed to narrow the long-standing inequalities by ethnicity. This is likely to require targeted public health education to enhance health literacy, the use of culturally sensitive screening strategies to improve uptake of risk factor screening and adherence to treatment, and greater community engagement.

Our study also has other important policy and research implications. Whilst disease specific population-based studies are still invaluable, they are costly and resource intensive. In contrast, we showed that linkage with overlapping health system data is feasible and can offer potentially more timely monitoring of ongoing trends in disease incidence. The combination of primary care data with hospital episodes is particularly important for the assessment of a condition like acute stroke, when a significant proportion of cases are not hospitalised in the UK [30]. Compared with a single source, linkage with multiple sources also helped to minimise bias related to changes in patient behaviour. This also highlights support for the ongoing effort, policy and initiatives from funding agencies to improve electronic data collection and integration between various data sources.

The strength of the study included the nationwide coverage and the large sample size, allowing study stroke incidence by age, sex, ethnicity, deprivation and region. There were, however, some limitations. First, data prior to 2018 were not available so we were not able to directly compare the trends that we observed after 2018 to what was happening before. For example, previous data from population-based studies in the UK suggested that there was already a trend of increase in stroke incidence at younger ages before 2018, which increased by 67% in 8 years [2]. In the current study, the increase in incidence was approximately 7% in 2 years. However, given the differences in the underlying population included, we were not able to assess whether this apparent slowing in acceleration since 2018 was genuinely a sign of stalling. Further monitoring remains crucial to understand the ongoing trends. Second, we did not have data on stroke aetiological subtypes and were not able to assess whether there were specific aetiologies that were driving the observed trends. Nevertheless, prevalences of diagnosed atrial fibrillation as well as treated atrial fibrillation remained largely unchanged during the study periods, suggesting that changes in the diagnosis and management of atrial fibrillation were unlikely a key mechanistic factor. Thirdly, stroke severity as measured by the NIHSS was only available for a subset of patients who were admitted primarily to acute stroke units. Therefore, we were not able to determine if there were changes in stroke severity more generally during the study periods. However, we previously showed that the increase in stroke at younger ages before 2018 was also seen for major strokes, although stroke tended to be milder at younger ages [2,31]. Fourth, age- and sex-specific population data were not available for smaller strata by ethnicity and region, or by ethnicity and deprivation; hence, we were not able to provide more detailed analyses looking at any synergistic effect with these metrics. There was also a very small percentage of patients without any data on ethnicity (<1%) or with potential misclassification (<5%) [32]. Fifth, GDPPR did not include participants who died before 1^st^ November 2019; hence, we might miss a very small group of patients who had strokes only recorded in primary care, and who had a stroke between 1st April 2018 and 31st October 2019 and died before 1^st^ November 2019. Sixth, we used age < 55 years to define “young stroke” based on previous work showing an age-specific divergence comparing age < 55 years versus age ≥ 55 years [2,4]. Although this cut-off is most commonly used in the young stroke literature, it is nevertheless arbitrary. Seventh, we used the presence of preventive medication as a surrogate marker to assess control of risk factors. However, we did not have measurements of blood pressure, lipid level, or diabetes control during the study periods. Eighth, we previously showed that there was similar magnitude of increase in stroke incidence at younger ages in those with versus without vascular risk factors [2]. As we did not have the age-specific risk factor prevalence data in more recent periods in the underlying population, we were not able to determine if the observed changes since 2018 were driven by patients with certain risk factor profiles. Finally, our analyses were based on record-linkage of health system data, which relies on the accuracy of diagnostic coding. Although studies showed that, compared to chart review, the positive predictive value was reasonable for identifying any stroke (89% for hospital admission codes, 80% for primary care codes) [30,33], the overall sensitivity was only 70% for hospitalised stroke [30], and further approaches to improve coding accuracy are still required. Moreover, data assessing the timeliness of stroke recording by various sources are also important for ongoing monitoring.

In conclusion, using nationwide health system data, we showed that the age-specific divergence in stroke incidence with a less favourable trend at younger ages continued to widen during the pandemic in England, especially for ethnic minority groups, and has not reversed since. This coincided with worsening in risk factor screening and diagnosis in primary care, highlighting missed opportunities that could have been addressed.

## Supporting information

S1 Checklist RECORD checklist. (DOCX)

S1 File Appendix A–V. **Appendix A:** Schematic representation of the definitions of prevalent, missed, and incident diagnosis of risk factors in relation to the index stroke. **Appendix B:** Calculation and interpretation of the relative temporal trend ratio (RTTR). **Appendix C:** Study flow chart. **Appendix D:** Age-standardised incidence (per 1,000) in England by each financial year between 2018 and 2024. **Appendix E:** Time trends of age-standardised stroke incidence (per 1,000) in England between 2018 and 2024 stratified by age, sex, ethnicity, deprivation, and stroke subtypes. **Appendix F:** Time trends of age-standardised stroke incidence (per 1,000) in England between 2018 and 2024 stratified by age and more detailed ethnicity groups. **Appendix G:** Time trends of age-standardised stroke incidence in England between 2018 and 2024 stratified by ascertainment methods. **Appendix H:** Proportions of fatal stroke between 2018–2024 in England. **Appendix I:** Distributions of NIHSS in SSNAP between 2018 and 2024 stratified by age. **Appendix J:** Distributions of NIHSS in SSNAP between 2018 and 2024 stratified by ethnicity. **Appendix K:** Time trends of incident, prevalent, missed, and treated vascular risk factors in England between 2018 and 2024. **Appendix L:** Time trends of incident, prevalent, missed, and treated vascular risk factors in England between 2018 and 2024 in Men. **Appendix M:** Time trends of incident, prevalent, missed, and treated vascular risk factors in England between 2018 and 2024 in Women. **Appendix N:** Time trends of incident, prevalent, missed, and treated vascular risk factors in England between 2018 and 2024 in people aged ≥ 55 years. **Appendix O:** Time trends of incident, prevalent, missed, and treated vascular risk factors in England between 2018 and 2024 in White people. **Appendix P:** Time trends of incident, prevalent, missed, and treated vascular risk factors in England between 2018 and 2024 in people aged < 55 years. **Appendix Q:** Time trends of incident, prevalent, missed, and treated vascular risk factors in England between 2018 and 2024 in Asian people. **Appendix R:** Time trends of incident, prevalent, missed, and treated vascular risk factors in England between 2018 and 2024 in Black people. **Appendix S:** Time trends of incident, prevalent, missed, and treated vascular risk factors in England between 2018 and 2024 in Black people aged <55 years. **Appendix T:** Time trends of incident, prevalent, missed, and treated vascular risk factors in England between 2018 and 2024 in Asian people aged <55 years. **Appendix U:** Time trends of incident, prevalent, missed, and treated vascular risk factors in England between 2018 and 2024 in White people aged <55 years. **Appendix V:** Prevalence of patients on relevant treatment for known modifiable stroke risk factors stratified by ethnic groups and time periods.
(DOCX)

## Abbreviations

BHF: British Heart Foundation
GDPPR: GP Extraction Service Data for Pandemic Planning and Research dataset
HES: Hospital Episodes Statistics
ICD 10: International Classification of Diseases 10th revision
IRR: incidence rate ratio
IRRs: incidence rate ratios
NHS: National Health Service
NIHSS: National Institutes of Health Stroke Scale
RR: risk ratio
RTTR: relative temporal trend ratio
SSNAP: Sentinel Stroke National Audit Programme.
