# Supplementary material for: Time trends in stroke incidence and modifiable risk factor prevalence in England (2018–2024): A record linkage population-based study
Source: PLoS Med. 2026 Aug 7;23(8):e1004999. doi: 10.1371/journal.pmed.1004999 (PMC13485125; doi:10.1371/journal.pmed.1004999)
Supplement: Supplementary file 2 [file pmed.1004999.s002.docx]

**Recent trends in stroke incidence and risk factor control in England (2018-2024): a record linkage population-based study**

Joseph Kamtchum-Tatuene, James Farrell, John Nolan, Sarah Lessels, William N Whiteley, Linxin Li , on behalf of the CVD-COVID-UK/COVID-IMPACT Consortium

**Appendix A** Schematic representation of the definitions of prevalent, missed, and incident diagnosis of risk factors in relation to the index stroke

**Appendix B** Calculation and interpretation of the relative temporal trend ratio (RTTR)

**Appendix C** Study flow chart

**Appendix D** Age-standardised incidence (per 1,000) in England by each financial year between 2018 and 2024

**Appendix E** Time trends of age-standardised stroke incidence (per 1,000) in England between 2018 and 2024 stratified by age, sex, ethnicity, deprivation, and stroke subtypes

**Appendix F** Time trends of age-standardised stroke incidence (per 1,000) in England between 2018 and 2024 stratified by age and more detailed ethnicity groups

**Appendix G** Time trends of age-standardised stroke incidence in England between 2018 and 2024 stratified by ascertainment methods

**Appendix H** Proportions of fatal stroke between 2018-2024 in England

**Appendix I** Distributions of NIHSS in SSNAP between 2018 and 2024 stratified by age

**Appendix J** Distributions of NIHSS in SSNAP between 2018 and 2024 stratified by ethnicity

**Appendix K** Time trends of incident, prevalent, missed, and treated vascular risk factors in England between 2018 and 2024

**Appendix L** Time trends of incident, prevalent, missed, and treated vascular risk factors in England between 2018 and 2024 in Men

**Appendix M** Time trends of incident, prevalent, missed, and treated vascular risk factors in England between 2018 and 2024 in Women

**Appendix N** Time trends of incident, prevalent, missed, and treated vascular risk factors in England between 2018 and 2024 in people aged ≥ 55 years

**Appendix O** Time trends of incident, prevalent, missed, and treated vascular risk factors in England between 2018 and 2024 in White people

**Appendix P** Time trends of incident, prevalent, missed, and treated vascular risk factors in England between 2018 and 2024 in people aged < 55 years

**Appendix Q** Time trends of incident, prevalent, missed, and treated vascular risk factors in England between 2018 and 2024 in Asian people

**Appendix R** Time trends of incident, prevalent, missed, and treated vascular risk factors in England between 2018 and 2024 in Black people

**Appendix S** Time trends of incident, prevalent, missed, and treated vascular risk factors in England between 2018 and 2024 in Black people aged <55 years

**Appendix T** Time trends of incident, prevalent, missed, and treated vascular risk factors in England between 2018 and 2024 in Asian people aged <55 years

**Appendix U** Time trends of incident, prevalent, missed, and treated vascular risk factors in England between 2018 and 2024 in White people aged <55 years

**Appendix V** Prevalence of patients on relevant treatment for known modifiable stroke risk factors stratified by ethnic groups and time periods

**Appendix A Schematic representation of the definitions of prevalent, missed, and incident diagnosis of risk factors in relation to the index stroke**

**Appendix B Calculation and interpretation of the relative temporal trend ratio (RTTR)**

A. **Where crude numbers are available for each time period**

Where: *_y_n_p0_*= number of new cases stroke age<55 years during earliest time period (p_0_)

*_y_N_p0_*= population of those age <55 years ×time during which cases were ascertained during p_0_

*_y_n_p1_*= number of new cases stroke age<55 years during latest time period (p_1_)

*_y_N_p1_*= population of those age <55 years ×time during which cases were ascertained during p_1_

*_o_n_p0_*= number of new cases stroke age ≥55 years during p_0_

*_o_N_p0_*= population of those age ≥55 years ×time during which cases were ascertained during p_0_

*_o_n_p1_*= number of new cases stroke age ≥55 years during p_1_

*_o_N_p1_*= population of those age ≥55 years ×time during which cases were ascertained during p_1_

IR=incidence rate; IRR=incidence rate ratio

1. IR for those aged under 55 years (_y_IR) during earliest time period (p_0_):

$${yIR}_{p0}=\frac{{yn}_{p0}}{{yN}_{p0}}$$

2. IR for those aged 55 years or older (_o_IR) during earliest time period (p_0_):

$${oIR}_{p0}=\frac{on_{p0}}{{oN}_{p0}}$$

3. IR for those aged under 55 years (_y_IR) during latest time period (p_1_):

$${yIR}_{p1}=\frac{{yn}_{p1}}{{yN}_{p1}}$$

4. IR for those aged 55 years or older (_o_IR) during latest time period (p_1_):

$${oIR}_{p1}=\frac{{on}_{p1}}{{oN}_{p1}}$$

5. IRR for those aged under 55 years (_y_IRR):

$$yIRR=\frac{{yIR}_{p1}}{{yIR}_{p0}}$$

6. IRR for those aged 55 years or older (_o_IRR):

$$oIRR=\frac{{oIR}_{p1}}{{oIR}_{p0}}$$

7. Relative temporal trend ratio (RTTR):

$$RTTR=\frac{\mathrm{yIRR}}{oIRR}$$

Uncertainty about the estimate of the RTTR due to chance were estimated by calculating the standard error (s.e) of the log (RTTR) as follows:

$$s.e.(logRTTR)=\sqrt{\frac{1}{{yn}_{p0}}-\frac{1}{{yN}_{p0}}+\frac{1}{{on}_{p0}}-\frac{1}{{oN}_{p0}}+\frac{1}{{yn}_{p1}}-\frac{1}{{yN}_{p1}}+\frac{1}{{on}_{p1}}-\frac{1}{{oN}_{p1}}}$$

B. **Where raw numbers were not reported but age specific IRR and 95% CI were reported:**

IRR and 95% CI were used to estimate SE as follows:

$$s.e.\left( IRR \right)=\frac{\left( \frac{ln (95\% upper limit of IRR) - ln(IRR)}{1.96} \right)+\left( \frac{ln(IRR) -ln (95\% lower limit of IRR)}{1.96} \right)}{2}$$

$$s.e.\left( RTTR \right)=\sqrt{{s.e(yIRR)}^{2}+{s.e(oIRR)}^{2}}$$

**Interpretation of RTTR**

The relative temporal trend ratio (RTTR) is a comparative measure to identify and quantify divergence in stroke incidence trends between younger and older people within a study. The RTTR is calculated from age-specific temporal incidence rate ratios (IRR), as demonstrated in the formula above. Here, we define IRR as the incidence rate in the latest reported time period divided by the incidence rate in the earliest reported time period. The RTTR is then calculated by dividing the IRR at younger ages by the IRR at older ages. The RTTR can therefore help identify age-specific differences in incidence trends which may be masked by overall incidence trends especially in those studies where the IRR at younger ages was also declining. While the RTTR is cruder than comparisons of age-specific average annual percent change (AAPC) in incidence, it allows a single summary comparison of age-specific incidence trends which can be measured in different studies reporting data in different age groups, at different time periods, including those which only reported at 2 time periods, and can ultimately be meta-analysed across studies.

The RTTR can be interpreted as follows:

- RTTR less than 1 would indicate that stroke incidence trends at younger ages were more favourable than stroke incidence trends at older ages.
- RTTR of 1 would indicate the same trends in stroke incidence were seen at younger and older ages.
- RTTR of greater than 1 would indicate that the stroke incidence trends at younger ages were less favourable than at older ages.

As the RTTR is a ratio of two measures, additional contextual information is required to interpret differences in the RTTR; for instance a RTTR of less than 1 could be driven either by a decrease in stroke incidence at younger ages or an increase in stroke incidence at older ages, or a combination of both to differing degrees. Two particular issues should also be understood:

1. The magnitude (but not the direction) of the RTTR will depend to some degree on the duration of the time period studied; therefore the RTTR should be interpreted with caution when comparing studies with very different durations. In a sensitivity analysis, we therefore attempted to standardise the RTTR to a 10-year study duration.

2. The RTTR can also be impacted by age-specific changes in population structure over time or any age-specific changes in completeness of case-ascertainment. For example, estimates of RTTR were based only on the crude incidence of stroke in each of the two age groups and would not therefore fully adjust for the ageing of the underlying study population over time. Any fall in age-specific stroke incidence in the older age group would be underestimated by the crude incidence rate due to the continuing population aging that will have occurred during these studies in high-income countries. Since this same bias would not be seen in the younger age group, the RTTR will tend to underestimate divergence.

**Appendix C Study flow chart**

GDPPR=GP Extraction Service Data for Pandemic Planning and Research dataset; HES= Hospital Episodes Statistics; SSNAP= Sentinel Stroke National Audit Programme; LSOA=Lower layer super output area.

GDPPR

(n=433,290)

HES

(n=468,495)

SSNAP

(n=354,680)

Index stroke n=832,625

(01/04/2018 - 31/03/2024)

Index stroke n=831,675

(01/04/2018 - 31/03/2024)

Index stroke n=812,010

(01/04/2018 - 31/03/2024)

First ever stroke n=652,840

(01/04/2018 - 31/03/2024)

First ever stroke n=643,885

(01/04/2018 - 31/03/2024)

Wrong event date n=945

Missing valid NHS number n=19,665

History of stroke n=159,170

Wrong age n=3,480

No valid LSOA n=5,205

Wrong/missing sex n=265

**Appendix D Age-standardised incidence (per 1,000) in England by each financial year between 2018 and 2024**

|  | **Overall**  **Incidence (95%CI)** | **<55 years**  **Incidence (95%CI)** | **≥55 years**  **Incidence (95%CI)** |
| --- | --- | --- | --- |
| **2018-2019** | 2.15 (2.13-2.16) | 0.47 (0.46-0.48) | 5.16 (5.12-5.19) |
| **2019-2020** | 2.18 (2.16-2.19) | 0.48 (0.47-0.49) | 5.22 (5.19-5.26) |
| **2020-2021** | 2.10 (2.09-2.11) | 0.49 (0.48-0.50) | 5.00 (4.97-5.03) |
| **2021-2022** | 2.24 (2.22-2.25) | 0.52 (0.51-0.53) | 5.31 (5.28-5.35) |
| **2022-2023** | 2.25 (2.23-2.26) | 0.53 (0.52-0.53) | 5.34 (5.30-5.37) |
| **2023-2024** | 2.25 (2.24-2.26) | 0.53 (0.52-0.54) | 5.33 (5.30-5.36) |

**Appendix E Time trends of age-standardised stroke incidence (per 1,000) in England between 2018 and 2024 stratified by age, sex, ethnicity, deprivation, and stroke subtypes**

|  | **2018-2020** | **2020-2022** | **2022-2024** | **2020-2022 vs. 2018-2020** | | **2022-2024 vs. 2020-2022** | | | | |  |  |  |
| --- | --- | --- | --- | --- | --- | --- | --- | --- | --- | --- | --- | --- | --- |
|  | **ASI (95%CI)** | **ASI (95%CI)** | **ASI (95%CI)** | **IRR (95%CI)** | **P** | **IRR (95%CI)** | | **P** | | |  |  |  |
| **Age** |  |  |  |  |  |  | |  | | |  |  |  |
| <55 years | 0.49 (0.48-0.49) | 0.52 (0.51-0.53) | 0.54 (0.53-0.55) | 1.07 (1.05-1.09) | <0.001 | 1.04 (1.02-1.06) | | <0.001 | | |  |  |  |
| ≥55 years | 5.19 (5.16-5.21) | 5.16 (5.13-5.18) | 5.34 (5.32-5.36) | 0.99 (0.99-1.00) | 0.06 | 1.04 (1.03-1.04) | | <0.001 | | |  |  |  |
|  |  |  |  |  |  |  | |  | | |  |  |  |
| **Sex** |  |  |  |  |  |  | |  | | |  |  |  |
| Male | 2.44 (2.43-2.45) | 2.47 (2.46-2.48) | 2.58 (2.57-2.59) | 1.01 (1.00-1.02) | 0.005 | 1.04 (1.04-1.05) | | <0.001 | | |  |  |  |
| Female | 1.91 (1.90-1.92) | 1.90 (1.89-1.91) | 1.96 (1.95-1.97) | 0.99 (0.99-1.00) | 0.24 | 1.03 (1.02-1.04) | | <0.001 | | |  |  |  |
|  |  |  |  |  |  |  | |  | | |  |  |  |
| **Ethnicity** |  |  |  |  |  |  | |  | | |  |  |  |
| White | 2.14 (2.13-2.15) | 2.14 (2.13-2.15) | 2.20 (2.19-2.21) | 1.00 (0.99-1.00) | 0.48 | 1.03 (1.02-1.04) | | <0.001 | | |  |  |  |
| Blacks | 2.46 (2.39-2.53) | 2.68 (2.61-2.75) | 3.04 (2.96-3.12) | 1.09 (1.05-1.13) | <0.001 | 1.13 (1.09-1.18) | | <0.001 | | |  |  |  |
| Asians | 2.28 (2.23-2.33) | 2.42 (2.37-2.47) | 2.69 (2.64-2.74) | 1.06 (1.03-1.09) | <0.001 | 1.11 (1.08-1.14) | | <0.001 | | |  |  |  |
|  |  |  |  |  |  | |  | | |  |  |  |  |
| **Deprivation** |  |  |  |  |  |  | |  |  |  |  |  |  |
| Quintile 1 (most deprived) | 2.77 (2.74-2.80) | 2.75 (2.72-2.78) | 2.84 (2.81-2.87) | 0.99 (0.98-1.01) | 0.30 | 1.03 (1.02-1.05) | | <0.001 |  |  |  |  |  |
| Quintile 2 | 2.35 (2.33-2.37) | 2.37 (2.35-2.39) | 2.42 (2.40-2.44) | 1.01 (0.99-1.02) | 0.22 | 1.02 (1.01-1.03) | | 0.002 |  |  |  |  |  |
| Quintile 3 | 2.12 (2.10-2.14) | 2.12 (2.10-2.13) | 2.18 (2.16-2.22) | 1.00 (0.98-1.01) | 0.58 | 1.03 (1.02-1.04) | | <0.001 |  |  |  |  |  |
| Quintile 4 | 1.94 (1.92-1.96) | 1.97 (1.95-1.99) | 2.07 (2.05-2.09) | 1.02 (1.00-1.03) | 0.03 | 1.05 (1.04-1.06) | | <0.001 |  |  |  |  |  |
| Quintile 5 (least deprived) | 1.81 (1.79-1.83) | 1.81 (1.79-1.83) | 1.90 (1.88-1.92) | 1.00 (0.99-1.01) | 0.99 | 1.05 (1.04-1.06) | | <0.001 |  |  |  |  |  |
|  |  |  |  |  |  |  | |  |  |  |  |  |  |
| **Subtype** |  |  |  |  |  |  | |  |  |  |  |  |  |
| Ischaemic stroke | 1.52 (1.52-1.53) | 1.53 (1.53-1.54) | 1.59 (1.58-1.60) | 1.01 (1.00-1.01) | 0.11 | 1.04 (1.03-1.04) | | <0.001 |  |  |  |  |  |
| Intracerebral haemorrhage | 0.25 (0.25-0.26) | 0.25 (0.25-0.26) | 0.26 (0.26-0.27) | 1.00 (0.99-1.02) | 0.66 | 1.04 (1.02-1.05) | | <0.001 |  |  |  |  |  |
| Subarachnoid haemorrhage | 0.09 (0.09-0.09) | 0.09 (0.09-0.09) | 0.09 (0.08-0.09) | 1.01 (0.98-1.04) | 0.47 | 0.96 (0.93-0.98) | | 0.003 |  |  |  |  |  |
| Unknown stroke | 0.30 (0.30-0.31) | 0.30 (0.30-0.30) | 0.32 (0.32-0.32) | 0.99 (0.97-1.00) | 0.12 | 1.06 (1.05-1.08) | | <0.001 |  |  |  |  |  |
|  |  |  |  |  |  |  | |  |  |  |  |  |  |
| **Region** |  |  |  |  |  |  | |  |  |  |  |  |  |
| East | 2.07 (2.04-2.10) | 2.05 (2.02-2.08) | 2.12 (2.09-2.15) | 0.99 (0.97-1.01) | 0.26 | 1.03 (1.02-1.05) | | 0.002 |  |  |  |  |  |
| East Midlands | 2.23 (2.20-2.26) | 2.28 (2.24-2.31) | 2.35 (2.32-2.38) | 1.02 (1.00-1.04) | 0.049 | 1.03 (1.01-1.05) | | 0.001 |  |  |  |  |  |
| London | 2.28 (2.25-2.31) | 2.24 (2.21-2.27) | 2.35 (2.32-2.38) | 0.98 (0.97-1.00) | 0.06 | 1.05 (1.03-1.07) | | <0.001 |  |  |  |  |  |
| North East | 2.61 (2.56-2.65) | 2.55 (2.50-2.59) | 2.69 (2.64-2.74) | 0.98 (0.95-1.00) | 0.08 | 1.06 (1.03-1.08) | | <0.001 |  |  |  |  |  |
| North West | 2.43 (2.40-2.46) | 2.42 (2.40-2.45) | 2.52 (2.49-2.55) | 1.00 (0.98-1.01) | 0.69 | 1.04 (1.02-1.06) | | <0.001 |  |  |  |  |  |
| South East | 2.11 (2.08-2.13) | 2.13 (2.11-2.16) | 2.14 (2.12-2.16) | 1.01 (1.00-1.03) | 0.10 | 1.00 (0.99-1.02) | | 0.66 |  |  |  |  |  |
| South West | 2.26 (2.23-2.29) | 2.21 (2.18-2.23) | 2.29 (2.26-2.32) | 0.98 (0.96-0.99) | 0.01 | 1.04 (1.02-1.06) | | <0.001 |  |  |  |  |  |
| West Midlands | 2.20 (2.17-2.22) | 2.18 (2.15-2.20) | 2.21 (2.18-2.24) | 0.99 (0.97-1.01) | 0.37 | 1.02 (1.00-1.03) | | 0.10 |  |  |  |  |  |
| Yorkshire | 2.39 (2.36-2.42) | 2.42 (2.39-2.45) | 2.59 (2.56-2.62) | 1.01 (1.00-1.03) | 0.14 | 1.07 (1.05-1.09) | | <0.001 |  |  |  |  |  |

**Appendix F Time trends of age-standardised stroke incidence (per 1,000) in England between 2018 and 2024 stratified by age and more detailed ethnicity groups**

|  | **2018-2020** | **2020-2022** | **2022-2024** | **2020-2022 vs. 2018-2020** | | **2022-2024 vs. 2020-2022** | |
| --- | --- | --- | --- | --- | --- | --- | --- |
|  | **ASI (95%CI)** | **ASI (95%CI)** | **ASI (95%CI)** | **IRR (95%CI)** | **p** | **IRR (95%CI)** | **p** |
| **<55 years** |  |  |  |  |  |  |  |
| **Black** |  |  |  |  |  |  |  |
| Black African | 0.56 (0.52-0.60) | 0.68 (0.64-0.72) | 0.79 (0.75-0.83) | 1.21 (1.11-1.32) | <0.0001 | 1.16 (1.07-1.26) | 0.0002 |
| Caribbean African | 0.60 (0.54-0.66) | 0.58 (0.52-0.64) | 0.61 (0.55-0.67) | 0.97 (0.84-1.11) | 0.64 | 1.05 (0.91-1.21) | 0.48 |
| **Asian** |  |  |  |  |  |  |  |
| Bangladeshi | 0.46 (0.41-0.51) | 0.62 (0.56-0.68) | 0.66 (0.59-0.73) | 1.35 (1.16-1.57) | 0.0001 | 1.06 (0.92-1.23) | 0.39 |
| Indian | 0.37 (0.34-0.40) | 0.45 (0.42-0.48) | 0.55 (0.52-0.58) | 1.22 (1.10-1.34) | <0.0001 | 1.22 (1.12-1.34) | <0.0001 |
| Pakistani | 0.51 (0.47-0.55) | 0.61 (0.57-0.65) | 0.70 (0.66-0.74) | 1.20 (1.09-1.32) | 0.0003 | 1.15 (1.05-1.26) | 0.003 |
| **≥55 years** |  |  |  |  |  |  |  |
| **Black** |  |  |  |  |  |  |  |
| Black African | 6.00 (5.62-6.38) | 6.75 (6.36-7.14) | 8.43 (7.99-8.87) | 1.13 (1.03-1.23) | 0.007 | 1.25 (1.16-1.35) | <0.0001 |
| Caribbean African | 5.11 (4.87-5.35) | 5.22 (4.98-5.46) | 5.67 (5.43-5.91) | 1.02 (0.96-1.09) | 0.52 | 1.09 (1.02-1.16) | 0.098 |
| **Asian** |  |  |  |  |  |  |  |
| Bangladeshi | 6.89 (6.33-7.45) | 7.52 (6.94-8.10) | 8.33 (7.73-8.93) | 1.09 (0.98-1.22) | 0.13 | 1.11 (1.00-1.23) | 0.06 |
| Indian | 5.07 (4.89-5.25) | 5.09 (4.91-5.27) | 5.51 (5.33-5.69) | 1.00 (0.96-1.05) | 0.88 | 1.08 (1.03-1.14) | 0.001 |
| Pakistani | 6.70 (6.40-7.00) | 6.63 (6.33-6.93) | 7.35 (7.04-7.66) | 0.99 (0.93-1.05) | 0.75 | 1.11 (1.04-1.18) | 0.0009 |

**Appendix G Time trends of age-standardised stroke incidence in England between 2018 and 2024 stratified by ascertainment methods**

|  | **2018-2020** | **2020-2022** | **2022-2024** | **2020-2022 vs. 2018-2020** | | **2022-2024 vs. 2020-2022** | |
| --- | --- | --- | --- | --- | --- | --- | --- |
|  | **ASI (95%CI)** | **ASI (95%CI)** | **ASI (95%CI)** | **IRR (95%CI)** | **p** | **IRR (95%CI)** | **p** |
| **Age<55 years** |  |  |  |  |  |  |  |
| GDPPR only | 0.34 (0.34-0.34) | 0.37 (0.37-0.38) | 0.38 (0.37-0.39) | 1.09 (1.07-1.11) | <0.001 | 1.02 (1.00-1.04) | 0.02 |
| HES only | 0.33 (0.33-0.33) | 0.35 (0.35-0.35) | 0.36 (0.35-0.37) | 1.06 (1.04-1.08) | <0.001 | 1.03 (1.01-1.05) | 0.006 |
| SSNAP only | 0.23 (0.23-0.23) | 0.22 (0.22-0.22) | 0.23 (0.23-0.23) | 0.96 (0.93-0.98) | 0.0005 | 1.05 (1.02-1.07) | <0.001 |
|  |  |  |  |  |  |  |  |
| **Age ≥ 55 years** |  |  |  |  |  |  |  |
| GDPPR only | 3.27 (3.25-3.29) | 3.60 (3.58-3.62) | 3.61 (3.59-3.62) | 1.10 (1.09-1.11) | <0.001 | 1.00 (0.99-1.01) | 0.83 |
| HES only | 3.89 (3.87-3.91) | 3.79 (3.77-3.81) | 3.84 (3.82-3.86) | 0.97 (0.97-0.98) | <0.001 | 1.01 (1.01-1.02) | <0.001 |
| SSNAP only | 3.33 (3.31-3.35) | 2.78 (2.76-2.80) | 2.80 (2.78-2.82) | 0.83 (0.82-0.84) | <0.001 | 1.01 (1.00-1.02) | 0.11 |
|  |  |  |  |  |  |  |  |
| **All** |  |  |  |  |  |  |  |
| GDPPR only | 1.39 (1.38-1.40) | 1.53 (1.52-1.53) | 1.53 (1.53-1.54) | 1.10 (1.09-1.11) | <0.001 | 1.00 (1.00-1.01) | 0.21 |
| HES only | 1.61 (1.60-1.62) | 1.58 (1.57-1.59) | 1.61 (1.60-1.62) | 0.98 (0.97-0.99) | <0.001 | 1.02 (1.01-1.03) | <0.001 |
| SSNAP only | 1.34 (1.33-1.35) | 1.14 (1.13-1.15) | 1.15 (1.14-1.16) | 0.85 (0.84-0.86) | <0.001 | 1.01 (1.00-1.02) | 0.04 |
|  |  |  |  |  |  |  |  |

GDPPR=GP Extraction Service Data for Pandemic Planning and Research dataset; HES= Hospital Episodes Statistics; SSNAP= Sentinel Stroke National Audit Programme

ASI=Age-standardised stroke incidence (standardised to the 2011 census population of England and Wales); IRR=Incidence rate ratio.

**Appendix H Proportions of fatal stroke between 2018-2024 in England**

|  | **2018-2020** | **2020-2022** | **2022-2024** | |
| --- | --- | --- | --- | --- |
| **Overall** |  |  |  |  |
| All stroke | 206290 | 211420 | 226175 | p<0.0001 |
| Fatal stroke | 24435 (11.8) | 25240 (11.9） | 24745 (10.9) | |
|  |  |  |  |  |
| **Age groups** |  |  |  |  |
| **<55 years** |  |  |  |  |
| All stroke | 26660 | 28250 | 29455 | p=0.13 |
| Fatal stroke | 1400 (5.3) | 1515 (5.4) | 1475 (5.0) | |
| **≥55 years** |  |  |  |  |
| All stroke | 179630 | 183170 | 196720 | p<0.0001 |
| Fatal stroke | 23040 (12.8) | 23725 (13.0) | 23270 (11.8) | |
|  |  |  |  |  |
| **Ethnicity** |  |  |  |  |
| **White** |  |  |  |  |
| All stroke | 185925 | 189240 | 200865 | p<0.0001 |
| Fatal stroke | 22245 (12.0) | 23195 (12.3) | 22805 (11.4) |  |
| **Blacks** |  |  |  |  |
| All stroke | 5325 | 6060 | 7055 | p=0.32 |
| Fatal stroke | 365 (6.9) | 435 (7.2) | 460 (6.6) |  |
| **Asians** |  |  |  |  |
| All stroke | 10150 | 11370 | 12895 | p=0.14 |
| Fatal stroke | 825 (8.1) | 955 (8.4) | 995 (7.7) |  |
|  |  |  |  |  |
| **Stroke subtype:** | |  |  |  |
| **Ischaemic stroke** | |  |  |  |
| All stroke | 145415 | 149385 | 160060 | p<0.0001 |
| Fatal stroke | 14750 (10.1) | 14485 (9.7) | 14225 (8.9) | |
| **Intracerebral haemorrhage** | | |  |  |
| All stroke | 23955 | 24605 | 26240 | p<0.0001 |
| Fatal stroke | 7275 (30.4) | 7445 (30.3) | 7450 (28.4) | |
| **Subarachnoid haemorrhage** | | |  |  |
| All stroke | 8045 | 8290 | 8045 | p=0.01 |
| Fatal stroke | 1745 (21.7) | 1650 (19.9) | 1625 (20.2) | |

**Appendix I Distributions of NIHSS in SSNAP between 2018 and 2024 stratified by age**

A. **Age<55 years** (p for trend =0.02)

|  | 0-5 | 6-9 | 10-14 | 15-19 | 20-24 | 25-29 | ≥30 |
| --- | --- | --- | --- | --- | --- | --- | --- |
| 2018-2020 | 8550 | 1795 | 765 | 725 | 325 | 205 | 110 |
| 2020-2022 | 8125 | 1640 | 755 | 685 | 290 | 155 | 90 |
| 2022-2024 | 8220 | 1755 | 805 | 700 | 305 | 125 | 95 |

B. **Age≥55 years** (p for trend <0.0001)

**NIHSS**

|  | 0-5 | 6-9 | 10-14 | 15-19 | 20-24 | 25-29 | ≥30 |
| --- | --- | --- | --- | --- | --- | --- | --- |
| 2018-2020 | 65230 | 17690 | 8310 | 8585 | 5205 | 3725 | 1920 |
| 2020-2022 | 58180 | 15060 | 6740 | 6835 | 4095 | 2810 | 1295 |
| 2022-2024 | 60275 | 16115 | 7370 | 7395 | 4235 | 2760 | 1195 |

**Appendix J Distributions of NIHSS in SSNAP between 2018 and 2024 stratified by ethnicity**

A. **White (p<0.0001)**

|  | 0-5 | 6-9 | 10-14 | 15-19 | 20-24 | 25-29 | ≥30 |
| --- | --- | --- | --- | --- | --- | --- | --- |
| 2018-2020 | 67300 | 17550 | 8220 | 8470 | 4985 | 3555 | 1835 |
| 2020-2022 | 60090 | 14980 | 6720 | 6755 | 3955 | 2680 | 1250 |
| 2022-2024 | 61600 | 15935 | 7280 | 7240 | 4030 | 2630 | 1180 |

B. **Asians (p=0.04)**

|  | 0-5 | 6-9 | 10-14 | 15-19 | 20-24 | 25-29 | ≥30 |
| --- | --- | --- | --- | --- | --- | --- | --- |
| 2018-2020 | 3305 | 990 | 405 | 380 | 240 | 150 | 75 |
| 2020-2022 | 3300 | 880 | 395 | 375 | 220 | 135 | 65 |
| 2022-2024 | 3645 | 955 | 455 | 415 | 260 | 110 | 60 |

C. **Blacks (p=0.80)**

**NIHSS**

|  | 0-5 | 6-9 | 10-14 | 15-19 | 20-24 | 25-29 | ≥30 |
| --- | --- | --- | --- | --- | --- | --- | --- |
| 2018-2020 | 1700 | 535 | 210 | 210 | 120 | 70 | 32 |
| 2020-2022 | 1640 | 510 | 225 | 215 | 115 | 70 | 30 |
| 2022-2024 | 1845 | 580 | 270 | 255 | 145 | 80 | 20 |

**Appendix K Time trends of incident, prevalent, missed, and treated vascular risk factors in England between 2018 and 2024**

|  | **2018-2020** (n=206290) | **2020-2022** (n=211420) | **2022-2024** (n=226175) | **2020-2022 vs 2018-2020** | | **2022-2024 vs 2020-2022** | |
| --- | --- | --- | --- | --- | --- | --- | --- |
|  |  |  |  | RR (95% CI) | p | RR (95% CI) | p |
| **Hypertension** | | | | | | | |
| Overall | 153335 (74.3) | 156555 (74.0) | 168445 (74.5) | 1.00 (0.99-1.00) | 0.04 | 1.01 (1.00-1.01) | 0.001 |
| Prevalent | 151540 (73.5) | 154845 (73.2) | 166525 (73.6) | 1.00 (0.99-1.00) | 0.11 | 1.01 (1.00-1.01) | 0.004 |
| Treated | 72905 (35.3) | 75015 (35.5) | 82440 (36.4) | 1.00 (1.00-1.01) | 0.34 | 1.03 (1.02-1.04) | <0.001 |
| Missed | 15580 (7.6) | 16195 (7.7) | 16520 (7.3) | 1.01 (0.99-1.03) | 0.29 | 0.95 (0.93-0.97) | <0.001 |
| Incident | 1795 (0.9) | 1710 (0.8) | 1920 (0.8) | 0.93 (0.87-0.99) | 0.03 | 1.05 (0.98-1.12) | 0.14 |
| **Diabetes mellitus** | | | | | | | |
| Overall | 53900 (26.1) | 56965 (26.9) | 64515 (28.5) | 1.03 (1.02-1.04) | <0.001 | 1.06 (1.05-1.07) | <0.001 |
| Prevalent | 53245 (25.8) | 56245 (26.6) | 63675 (28.1) | 1.03 (1.02-1.04) | <0.001 | 1.06 (1.05-1.07) | <0.001 |
| Treated | 32710 (15.9) | 35110 (16.6) | 41010 (18.1) | 1.05 (1.03-1.06) | <0.001 | 1.09 (1.08-1.11) | <0.001 |
| Missed | 3680 (1.8) | 3700 (1.8) | 4135 (1.8) | 0.98 (0.94-1.03) | 0.41 | 1.05 (1.00-1.09) | 0.05 |
| Incident | 655 (0.3) | 720 (0.3) | 840 (0.4) | 1.07 (0.97-1.19) | 0.19 | 1.09 (1.00-1.20) | 0.09 |
| **Hyperlipidemia** | | | | | | | |
| Overall | 108165 (52.4) | 114390 (54.1) | 129270 (57.2) | 1.03 (1.03-1.04) | <0.001 | 1.06 (1.05-1.06) | <0.001 |
| Prevalent | 107575 (52.1) | 113850 (53.8) | 128700 (56.9) | 1.03 (1.03-1.04) | <0.001 | 1.06 (1.05-1.06) | <0.001 |
| Treated | 86755 (42.0) | 93280 (44.2) | 106425 (47.1) | 1.05 (1.04-1.06) | <0.001 | 1.07 (1.06-1.07) | <0.001 |
| Missed | 7890 (3.8) | 8115 (3.8) | 8945 (4.0) | 1.00 (0.97-1.03) | 0.82 | 1.03 (1.00-1.06) | 0.046 |
| Incident | 590 (0.3) | 540 (0.3) | 570 (0.3) | 0.89 (0.80-1.00) | 0.06 | 0.99 (0.88-1.11) | 0.82 |
| **Atrial fibrillation** | | | | | | | |
| Overall | 64170 (31.1) | 63540 (30.1) | 68485 (30.3) | 0.97 (0.96-0.98) | <0.001 | 1.01 (1.00-1.02) | 0.10 |
| Prevalent | 62000 (30.0) | 61530 (29.1) | 66335 (29.3) | 0.97 (0.96-0.98) | <0.001 | 1.01 (1.00-1.02) | 0.10 |
| Treated | 30295 (14.7) | 31695 (15.0) | 36075 (16.0) | 1.02 (1.01-1.04) | 0.005 | 1.06 (1.05-1.08) | <0.001 |
| Missed | 15820 (7.7) | 15180 (7.2) | 15895 (7.0) | 0.94 (0.92-0.96) | <0.001 | 0.98 (0.96-1.00) | 0.05 |
| Incident | 2170 (1.1) | 2010 (1.0) | 2150 (1.0) | 0.90 (0.85-0.96) | 0.001 | 1.00 (0.94-1.06) | 0.99 |

Numbers are presented as n (%). RR=risk ratio

**Appendix L Time trends of incident, prevalent, missed, and treated vascular risk factors in England between 2018 and 2024 in Men**

|  | **2018-2020**  (n=105780) | **2020-2022** (n=109880) | **2022-2024** (n=118120) | **2020-2022 vs 2018-2020** | | **2022-2024 vs 2020-2022** | |
| --- | --- | --- | --- | --- | --- | --- | --- |
|  |  |  |  | RR (95%CI) | p | RR (95%CI) | p |
| **Hypertension** | | | | | | | |
| Overall | 77585 (73.3) | 80690 (73.4) | 87420 (74.0) | 1.00 (1.00-1.01) | 0.64 | 1.01 (1.00-1.01) | 0.002 |
| Prevalent | 76575 (72.4) | 79710 (72.5) | 86320 (73.1) | 1.00 (1.00-1.01) | 0.43 | 1.01 (1.00-1.01) | 0.004 |
| Treated | 38790 (36.7) | 40155 (36.5) | 44675 (37.8) | 1.00 (0.99-1.01) | 0.54 | 1.04 (1.02-1.05) | <0.001 |
| Missed | 8765 (8.3) | 9495 (8.6) | 9555 (8.1) | 1.04 (1.01-1.07) | 0.003 | 0.94 (0.91-0.96) | <0.001 |
| Incident | 1010 (1.0) | 980 (0.9) | 1100 (0.9) | 0.93 (0.86-1.02) | 0.13 | 1.04 (0.96-1.14) | 0.32 |
| **Diabetes mellitus** | | | | | | | |
| Overall | 29840 (28.2) | 31945 (29.1) | 36425 (30.8) | 1.03 (1.02-1.04) | <0.001 | 1.06 (1.05-1.07) | <0.001 |
| Prevalent | 29415 (27.8) | 31535 (28.7) | 35905 (30.4) | 1.03 (1.02-1.05) | <0.001 | 1.06 (1.05-1.07) | <0.001 |
| Treated | 18725 (17.7) | 20520 (18.7) | 24140 (20.4) | 1.06 (1.04-1.07) | <0.001 | 1.09 (1.05-1.11) | <0.001 |
| Missed | 2050 (1.9) | 2135 (1.9) | 2345 (2.0) | 1.00 (0.94-1.07) | 0.93 | 1.02 (0.96-1.08) | 0.47 |
| Incident | 425 (0.4) | 410 (0.4) | 520 (0.4) | 0.93 (0.81-1.06) | 0.28 | 1.18 (1.04-1.34) | 0.01 |
| **Hyperlipidemia** | | | | | | | |
| Overall | 58075 (54.9) | 61825 (56.3) | 69810 (59.1) | 1.03 (1.02-1.03) | <0.001 | 1.05 (1.04-1.06) | <0.001 |
| Prevalent | 57770 (54.6) | 61515 (56.0) | 69485 (58.8) | 1.03 (1.02-1.03) | <0.001 | 1.05 (1.04-1.06) | <0.001 |
| Treated | 47505 (44.9) | 51680 (47.0) | 58785 (49.8) | 1.05 (1.04-1.06) | <0.001 | 1.06 (1.05-1.07) | <0.001 |
| Missed | 4280 (4.0) | 4370 (4.0) | 4785 (4.1) | 0.98 (0.94-1.02) | 0.41 | 1.02 (0.98-1.06) | 0.37 |
| Incident | 305 (0.3) | 310 (0.3) | 325 (0.3) | 0.98 (0.84-1.15) | 0.79 | 0.98 (0.84-1.14) | 0.75 |
| **Atrial fibrillation** | | | | | | | |
| Overall | 32210 (30.4) | 32285 (29.4) | 35660 (30.2) | 0.97 (0.95-0.98) | <0.001 | 1.03 (1.02-1.04) | <0.001 |
| Prevalent | 31040 (29.3) | 31210 (28.4) | 34510 (29.2) | 0.97 (0.96-0.98) | <0.001 | 1.03 (1.02-1.04) | <0.001 |
| Treated | 15675 (14.8) | 16615 (15.1) | 19220 (16.3) | 1.02 (1.00-1.04) | 0.049 | 1.08 (1.06-1.10) | <0.001 |
| Missed | 7610 (7.2) | 7200 (6.6) | 7880 (6.7) | 0.91 (0.88-0.94) | <0.001 | 1.02 (0.99-1.05) | 0.26 |
| Incident | 1170 (1.1) | 1075 (1.0) | 1150 (1.0) | 0.89 (0.81-0.96) | 0.003 | 1.00 (0.92-1.08) | 0.91 |

Numbers are presented as n (%). RR=risk ratio

**Appendix M Time trends of incident, prevalent, missed, and treated vascular risk factors in England between 2018 and 2024 in Women**

|  | **2018-2020** (n=100510) | **2020-2022**  (n=101540) | **2022-2024**  (n=108055) | **2020-2022 vs 2018-2020** | | **2022-2024 vs 2020-2022** | |
| --- | --- | --- | --- | --- | --- | --- | --- |
|  |  |  |  | RR (95%CI) | p | RR (95%CI) | p |
| **Hypertension** | | | | | | | |
| Overall | 75750 (75.4) | 75865 (74.7) | 81025 (75.0) | 0.99 (0.99-1.00) | <0.001 | 1.00 (1.00-1.01) | 0.15 |
| Prevalent | 74965 (74.6) | 75135 (74.0) | 80205 (74.2) | 0.99 (0.99-1.00) | 0.002 | 1.00 (1.00-1.01) | 0.23 |
| Treated | 34115 (33.9) | 34860 (34.3) | 37765 (34.9) | 1.01 (1.00-1.02) | 0.07 | 1.02 (1.01-1.03) | 0.003 |
| Missed | 6815 (6.8) | 6700 (6.6) | 6965 (6.4) | 0.97 (0.94-1.01) | 0.10 | 0.98 (0.95-1.01) | 0.16 |
| Incident | 785 (0.8) | 730 (0.7) | 820 (0.8) | 0.92 (0.83-1.02) | 0.11 | 1.06 (0.96-1.17) | 0.29 |
| **Diabetes mellitus** | | | | | | | |
| Overall | 24060 (23.9) | 25020 (24.6) | 28090 (26.0) | 1.03 (1.01-1.05) | <0.001 | 1.06 (1.04-1.07) | <0.001 |
| Prevalent | 23830 (23.7) | 24710 (24.3) | 27770 (25.7) | 1.03 (1.01-1.04) | 0.001 | 1.06 (1.04-1.07) | <0.001 |
| Treated | 13985 (13.9) | 14590 (14.4) | 16870 (15.6) | 1.03 (1.01-1.06) | 0.003 | 1.09 (1.06-1.11) | <0.001 |
| Missed | 1630 (1.6) | 1565 (1.5) | 1790 (1.7) | 0.95 (0.89-1.02) | 0.15 | 1.08 (1.00-1.15) | 0.04 |
| Incident | 230 (0.2) | 310 (0.3) | 320 (0.3) | 1.33 (1.13-1.58) | <0.001 | 0.97 (0.83-1.13) | 0.70 |
| **Hyperlipidemia** | | | | | | | |
| Overall | 50090 (49.8) | 52565 (51.8) | 59460 (55.0) | 1.04 (1.03-1.05) | <0.001 | 1.06 (1.05-1.07) | <0.001 |
| Prevalent | 49805 (49.6) | 52335 (51.5) | 59215 (54.8) | 1.04 (1.03-1.05) | <0.001 | 1.06 (1.06-1.07) | <0.001 |
| Treated | 39250 (39.1) | 41600 (41.0) | 47640 (44.1) | 1.05 (1.04-1.06) | <0.001 | 1.08 (1.07-1.09) | <0.001 |
| Missed | 3610 (3.6) | 3745 (3.7) | 4160 (3.8) | 1.03 (0.98-1.07) | 0.25 | 1.04 (1.00-1.09) | 0.05 |
| Incident | 285 (0.3) | 230 (0.2) | 245 (0.2) | 0.80 (0.67-0.95) | 0.01 | 1.00 (0.84-1.20) | 0.99 |
| **Atrial fibrillation** | | | | | | | |
| Overall | 31960 (31.8) | 31255 (30.8) | 32825 (30.4) | 0.97 (0.96-0.98) | <0.001 | 0.99 (0.97-1.00) | 0.045 |
| Prevalent | 30960 (30.8) | 30320 (29.9) | 31825 (29.5) | 0.97 (0.96-0.98) | <0.001 | 0.99 (0.97-1.00) | 0.04 |
| Treated | 14620 (14.5) | 15080 (14.9) | 16855 (15.6) | 1.02 (1.00-1.04) | 0.05 | 1.05 (1.03-1.07) | <0.001 |
| Missed | 8210 (8.2) | 7980 (7.9) | 8015 (7.4) | 0.96 (0.93-0.99) | 0.01 | 0.94 (0.92-0.97) | <0.001 |
| Incident | 1000 (1.0) | 935 (0.9) | 1000 (0.9) | 0.93 (0.85-1.01) | 0.09 | 1.01 (0.92-1.10) | 0.91 |

Numbers are presented as n (%). RR=risk ratio

**Appendix N Time trends of incident, prevalent, missed, and treated vascular risk factors in England between 2018 and 2024 in people aged ≥ 55 years**

|  | **2018-2022** (n=179630) | **2020-2022** (n=183170) | **2022-2024** (n=196720) | **2020-2022 vs 2018-2020** | | **2022-2024 vs 2020-2022** | |
| --- | --- | --- | --- | --- | --- | --- | --- |
|  |  |  |  | RR (95% CI) | p | RR (95% CI) | p |
| **Hypertension** | | | | | | | |
| Overall | 141335 (78.7) | 143595 (78.4) | 154615 (78.6) | 1.00 (0.99-1.00) | 0.04 | 1.00 (1.00-1.01) | 0.13 |
| Prevalent | 139885 (77.9) | 142225 (77.6) | 153095 (77.8) | 1.00 (0.99-1.00) | 0.10 | 1.00 (1.00-1.01) | 0.19 |
| Treated | 67990 (37.9) | 69700 (38.1) | 76440 (38.9) | 1.01 (1.00-1.01) | 0.21 | 1.02 (1.01-1.03) | <0.001 |
| Missed | 12695 (7.1) | 12915 (7.1) | 13145 (6.7) | 1.00 (0.97-1.02) | 0.85 | 0.95 (0.93-0.97) | <0.001 |
| Incident | 1450 (0.8) | 1370 (0.7) | 1520 (0.8) | 0.93 (0.86-1.00) | 0.04 | 1.03 (0.96-1.11) | 0.38 |
| **Diabetes mellitus** | | | | | | | |
| Overall | 49540 (27.6) | 52050 (28.4) | 59110 (30.0) | 1.03 (1.02-1.04) | <0.001 | 1.06 (1.05-1.07) | <0.001 |
| Prevalent | 49000 (27.3) | 51465 (28.1) | 58430 (29.7) | 1.03 (1.02-1.04) | <0.001 | 1.06 (1.05-1.07) | <0.001 |
| Treated | 30045 (16.7) | 32115 (17.5) | 37575 (19.1) | 1.05 (1.03-1.06) | <0.001 | 1.09 (1.08-1.10) | <0.001 |
| Missed | 3215 (1.8) | 3095 (1.7) | 3485 (1.8) | 0.94 (0.89-0.99) | 0.02 | 1.05 (1.00-1.10) | 0.05 |
| Incident | 540 (0.3) | 585 (0.3) | 680 (0.3) | 1.06 (0.95-1.19) | 0.31 | 1.08 (0.97-1.21) | 0.16 |
| **Hyperlipidemia** | | | | | | | |
| Overall | 101065 (56.3) | 106485 (58.1) | 120510 (61.3) | 1.03 (1.03-1.04) | <0.001 | 1.05 (1.05-1.06) | <0.001 |
| Prevalent | 100575 (56.0) | 106045(57.9) | 120055 (61.0) | 1.03 (1.03-1.04) | <0.001 | 1.05 (1.05-1.06) | <0.001 |
| Treated | 81560 (45.4) | 87395 (47.7) | 99875 (50.8) | 1.05 (1.04-1.06) | <0.001 | 1.06 (1.06-1.07) | <0.001 |
| Missed | 6640 (3.7) | 6760 (3.7) | 7390 (3.8) | 1.00 (0.97-1.03) | 0.93 | 1.02 (0.99-1.05) | 0.28 |
| Incident | 490 (0.3) | 440 (0.2) | 455 (0.2) | 0.88 (0.77-1.00) | 0.05 | 0.96 (0.85-1.10) | 0.57 |
| **Atrial fibrillation** | | | | | | | |
| Overall | 62110 (34.6) | 61375 (33.5) | 66140 (33.6) | 0.97 (0.90-0.98) | <0.001 | 1.00 (0.99-1.01) | 0.46 |
| Prevalent | 60050 (33.4) | 59450 (32.5) | 64105 (32.6) | 0.97 (0.93-0.98) | <0.001 | 1.00 (0.99-1.01) | 0.39 |
| Treated | 29210 (16.3) | 30515 (16.7) | 34825 (17.7) | 1.02 (1.01-1.04) | 0.001 | 1.06 (1.05-1.08) | <0.001 |
| Missed | 15250 (8.5) | 14640 (8.0) | 15320 (7.8) | 0.94 (0.92-0.96) | <0.001 | 0.97 (0.95-1.00) | 0.02 |
| Incident | 2060 (1.1) | 1925 (1.1) | 2035 (1.0) | 0.92 (0.86-0.98) | 0.006 | 0.98 (0.93-1.05) | 0.62 |

Numbers are presented as n (%). RR=risk ratio

**Appendix O Time trends of incident, prevalent, missed, and treated vascular risk factors in England between 2018 and 2024 in White people**

|  | **2018-2020**  (n=185925) | **2020-2022**  (n=189240) | **2022-2024**  (n=200865) | **2020-2022 vs 2018-2020** | | **2022-2024 vs 2020-2022** | |
| --- | --- | --- | --- | --- | --- | --- | --- |
|  |  |  |  | RR (95%CI) | p | RR (95%CI) | p |
| **Hypertension** | | | | | | | |
| Overall | 137620 (74.0) | 139550 (73.7) | 149095 (74.2) | 1.00 (0.99-1.00) | 0.05 | 1.01 (1.00-1.01) | <0.001 |
| Prevalent | 135990 (73.1) | 138030 (72.9) | 147380 (73.4) | 1.00 (0.99-1.00) | 0.16 | 1.01 (1.00-1.01) | 0.002 |
| Treated | 65165 (35.0) | 66650 (35.2) | 72795 (36.2) | 1.01 (1.00-1.01) | 0.27 | 1.03 (1.02-1.04) | <0.001 |
| Missed | 13635 (7.3) | 13975 (7.4) | 13940 (6.9) | 1.01 (0.98-1.03) | 0.55 | 0.94 (0.92-0.96) | <0.001 |
| Incident | 1630 (0.9) | 1520 (0.8) | 1715 (0.9) | 0.92 (0.85-0.98) | 0.01 | 1.06 (0.99-1.14) | 0.08 |
| **Diabetes mellitus** | | | | | | | |
| Overall | 45035 (24.2) | 47270 (25.0) | 53135 (26.5) | 1.03 (1.02-1.04) | <0.001 | 1.06 (1.05-1.07) | <0.001 |
| Prevalent | 44490 (23.9) | 46690 (24.7) | 52460 (26.1) | 1.03 (1.02-1.04) | <0.001 | 1.06 (1.05-1.07) | <0.001 |
| Treated | 26885 (14.5) | 28660 (15.1) | 33295 (16.6) | 1.05 (1.03-1.06) | <0.001 | 1.09 (1.08-1.11) | <0.001 |
| Missed | 3065 (1.6) | 3000 (1.6) | 3335 (1.7) | 0.96 (0.92-1.01) | 0.13 | 1.05 (1.00-1.10) | 0.06 |
| Incident | 545 (0.3) | 580 (0.3) | 675 (0.3) | 1.05 (0.93-1.18) | 0.45 | 1.10 (0.98-1.23) | 0.10 |
| **Hyperlipidemia** | | | | | | | |
| Overall | 97055 (52.2) | 102225 (54.0) | 114925 (57.2) | 1.04 (1.03-1.04) | <0.001 | 1.06 (1.05-1.07) | <0.001 |
| Prevalent | 96550 (51.9) | 101765 (53.8) | 114435 (57.0) | 1.04 (1.03-1.04) | <0.001 | 1.06 (1.05-1.07) | <0.001 |
| Treated | 78090 (42.0) | 83545 (44.1) | 94975 (47.3) | 1.05 (1.04-1.06) | <0.001 | 1.07 (1.06-1.08) | <0.001 |
| Missed | 6925 (3.7) | 7115 (3.8) | 7680 (3.8) | 1.01 (0.98-1.04) | 0.57 | 1.02 (0.99-1.05) | 0.30 |
| Incident | 505 (0.3) | 460 (0.2) | 490 (0.2) | 0.90 (0.79-1.02) | 0.08 | 1.00 (0.88-1.14) | 0.96 |
| **Atrial fibrillation** | | | | | | | |
| Overall | 60230 (32.4) | 59615 (31.5) | 63990 (31.9) | 0.97 (0.96-0.98) | <0.001 | 1.01 (1.00-1.02) | 0.02 |
| Prevalent | 58185 (31.3) | 57755 (30.5) | 62015 (30.9) | 0.98 (0.97-0.98) | <0.001 | 1.01 (1.00-1.02) | 0.02 |
| Treated | 28580 (15.4) | 29970 (15.8) | 33965 (16.9) | 1.03 (1.02-1.05) | <0.001 | 1.07 (1.05-1.08) | <0.001 |
| Missed | 14635 (7.9) | 14020 (7.4) | 14640 (7.3) | 0.94 (0.92-0.96) | <0.001 | 0.98 (0.96-1.01) | 0.15 |
| Incident | 2045 (1.1) | 1860 (1.0) | 1975 (1.0) | 0.89 (0.84-0.95) | <0.001 | 1.00 (0.94-1.07) | 0.99 |

Numbers are presented as n (%). RR=risk ratio

**Appendix P Time trends of incident, prevalent, missed, and treated vascular risk factors in England between 2018 and 2024 in people aged < 55 years**

|  | **2018-2020** (n=26660) | **2020-2022** (n=28250) | **2022-2024** (n=29455) | **2020-2022 vs 2018-2020** | | **2022-2024 vs 2020-2022** | |
| --- | --- | --- | --- | --- | --- | --- | --- |
|  |  |  |  | RR (95% CI) | p | RR (95% CI) | p |
| **Hypertension** | | | | | | | |
| Overall | 12000 (45.0) | 12960 (45.9) | 13835 (47.0) | 1.02 (1.00-1.04) | 0.04 | 1.02 (1.01-1.04) | 0.008 |
| Prevalent | 11655 (43.7) | 12620 (44.7) | 13435 (45.6) | 1.02 (1.00-1.04) | 0.02 | 1.02 (1.00-1.04) | 0.02 |
| Treated | 4915 (18.4) | 5315 (18.8) | 6000 (20.4) | 1.02 (0.99-1.06) | 0.26 | 1.08 (1.05-1.12) | <0.001 |
| Missed | 2885 (10.8) | 3280 (11.6) | 3375 (11.5) | 1.07 (1.02-1.13) | 0.003 | 0.99 (0.94-1.03) | 0.57 |
| Incident | 345 (1.3) | 340 (1.2) | 400 (1.4) | 0.93 (0.80-1.08) | 0.34 | 1.13 (0.98-1.30) | 0.10 |
| **Diabetes mellitus** | | | | | | | |
| Overall | 4360 (16.4) | 4910 (17.4) | 5405 (18.4) | 1.06 (1.02-1.10) | 0.001 | 1.06 (1.02-1.09) | 0.002 |
| Prevalent | 4240 (15.9) | 4775 (16.9) | 5245 (17.8) | 1.06 (1.02-1.10) | 0.001 | 1.05 (1.02-1.09) | 0.004 |
| Treated | 2660 (10.0) | 2990 (10.6) | 3435 (11.7) | 1.06 (1.01-1.12) | 0.02 | 1.10 (1.02-1.15) | <0.001 |
| Missed | 470 (1.8) | 605 (2.1) | 650 (2.2) | 1.22 (1.08-1.37) | 0.001 | 1.03 (0.92-1.15) | 0.59 |
| Incident | 120 (0.5) | 135 (0.5) | 160 (0.5) | 1.06 (0.83-1.36) | 0.63 | 1.14 (0.90-1.43) | 0.27 |
| **Hyperlipidemia** | | | | | | | |
| Overall | 7100 (26.6) | 7905 (28.0) | 8755 (29.7) | 1.05 (1.02-1.08) | <0.001 | 1.06 (1.04-1.09) | <0.001 |
| Prevalent | 7000 (26.3) | 7810 (27.6) | 8640 (29.3) | 1.05 (1.02-1.08) | <0.001 | 1.06 (1.03-1.09) | <0.001 |
| Treated | 5200 (19.5) | 5890 (20.8) | 6545 (22.2) | 1.07 (1.03-1.11) | <0.001 | 1.07 (1.03-1.10) | <0.001 |
| Missed | 1255 (4.7) | 1350 (4.8) | 1555 (5.3) | 1.02 (0.94-1.09) | 0.69 | 1.11 (1.03-1.19) | 0.006 |
| Incident | 100 (0.4) | 95 (0.3) | 115 (0.4) | 0.90 (0.68-1.19) | 0.45 | 1.16 (0.89-1.52) | 0.28 |
| **Atrial fibrillation** | | | | | | | |
| Overall | 2060 (7.7) | 2170 (7.7) | 2350 (8.0) | 0.99 (0.94-1.05) | 0.84 | 1.04 (0.98-1.10) | 0.19 |
| Prevalent | 1955 (7.3) | 2080 (7.4) | 2235 (7.6) | 1.00 (0.95-1.07) | 0.89 | 1.03 (0.97-1.09) | 0.30 |
| Treated | 1090 (4.1) | 1185 (4.2) | 1250 (4.2) | 1.03 (0.95-1.11) | 0.53 | 1.01 (0.94-1.09) | 0.77 |
| Missed | 570 (2.1) | 535 (1.9) | 575 (2.0) | 0.89 (0.79-1.00) | 0.04 | 1.03 (0.92-1.16) | 0.61 |
| Incident | 105 (0.4) | 90 (0.3) | 115 (0.4) | 0.81 (0.61-1.07) | 0.14 | 1.23 (0.93-1.61) | 0.15 |

Numbers are presented as n (%). RR=risk ratio

**Appendix Q Time trends of incident, prevalent, missed, and treated vascular risk factors in England between 2018 and 2024 in Asian people**

|  | **2018-2020** (n=10150) | **2020-2022** (n=11370) | **2022-2024** (n=12895) | **2020-2022 vs 2018-2020** | | **2022-2024 vs 2020-2022** | |
| --- | --- | --- | --- | --- | --- | --- | --- |
|  |  |  |  | RR (95% CI) | p | RR (95% CI) | p |
| **Hypertension** | | | | | | | |
| Overall | 8060 (79.4) | 8840 (77.7) | 10040 (77.8) | 0.98 (0.97-0.99) | 0.003 | 1.00 (0.99-1.02) | 0.88 |
| Prevalent | 7975 (78.6) | 8745 (76.9) | 9925 (76.9) | 0.98 (0.97-0.99) | 0.003 | 1.00 (0.99-1.01) | 0.96 |
| Treated | 4355 (42.9) | 4735 (41.6) | 5530 (42.9) | 0.97 (0.94-1.00) | 0.06 | 1.03 (0.99-1.06) | 0.05 |
| Missed | 835 (8.2) | 1065 (9.4) | 1150 (8.9) | 1.14 (1.04-1.24) | 0.003 | 0.95 (0.88-1.03) | 0.22 |
| Incident | 85 (0.8) | 95 (0.8) | 115 (0.9) | 1.00 (0.75-1.34) | 0.99 | 1.07 (0.81-1.40) | 0.64 |
| **Diabetes mellitus** | | | | | | | |
| Overall | 5275 (52.0) | 5810 (51.1) | 6710 (52.0) | 0.98 (0.96-1.01) | 0.20 | 1.02 (0.99-1.04) | 0.15 |
| Prevalent | 5220 (51.4) | 5740 (50.5) | 6625 (51.4) | 0.98 (0.96-1.01) | 0.17 | 1.02 (0.99-1.04) | 0.18 |
| Treated | 3660 (36.1) | 4040 (35.5) | 4785 (37.1) | 0.99 (0.95-1.02) | 0.42 | 1.04 (1.01-1.08) | 0.01 |
| Missed | 295 (2.9) | 355 (3.1) | 395 (3.1) | 1.07 (0.92-1.25) | 0.36 | 0.98 (0.85-1.13) | 0.79 |
| Incident | 55 (0.5) | 70 (0.6) | 85 (0.7) | 1.14 (0.80-1.62) | 0.48 | 1.07 (0.78-1.47) | 0.67 |
| **Hyperlipidemia** | | | | | | | |
| Overall | 6455 (63.6) | 7085 (62.3) | 8300 (64.3) | 0.98 (0.96-1.000 | 0.05 | 1.03 (1.01-1.05) | 0.001 |
| Prevalent | 6410 (63.2) | 7050 (62.0) | 8265 (64.1) | 0.98 (0.96-1.00) | 0.08 | 1.03 (1.01-1.05) | 0.001 |
| Treated | 5145 (50.7) | 5825 (51.2) | 6855 (53.1) | 1.01 (0.98-1.04) | 0.43 | 1.04 (1.01-1.06) | 0.003 |
| Missed | 470 (4.6) | 485 (4.3) | 610 (4.7) | 0.92 (0.81-1.04) | 0.19 | 1.11 (0.99-1.25) | 0.08 |
| Incident | 45 (0.4) | 35 (0.3) | 35 (0.3) | 0.69 (0.45-1.08) | 0.10 | 0.88 (0.55-1.41) | 0.60 |
| **Atrial fibrillation** | | | | | | | |
| Overall | 1860 (18.3) | 1925 (16.9) | 2195 (17.0) | 0.92 (0.87-0.98) | 0.007 | 1.01 (0.95-1.06) | 0.86 |
| Prevalent | 1800 (17.7) | 1840 (16.2) | 2110 (16.4) | 0.91 (0.86-0.97) | 0.002 | 1.01 (0.95-1.07) | 0.71 |
| Treated | 820 (8.1) | 845 (7.4) | 1015 (7.9) | 0.92 (0.84-1.01) | 0.08 | 1.06 (0.97-1.16) | 0.20 |
| Missed | 480 (4.7) | 530 (4.7) | 590 (4.6) | 0.99 (0.87-1.11) | 0.82 | 0.98 (0.88-1.10) | 0.75 |
| Incident | 60 (0.6) | 85 (0.7) | 85 (0.7) | 1.27 (0.91-1.76) | 0.16 | 0.88 (0.65-1.19) | 0.41 |

Numbers are presented as n (%). RR=risk ratio

**Appendix R Time trends of incident, prevalent, missed, and treated vascular risk factors in England between 2018 and 2024 in Black people**

|  | **2018-2020** (n=5325) | **2020-2022** (n=6060) | **2022-2024** (n=7055) | **2020-2022 vs 2018-2020** | | **2022-2024 vs 2020-2022** | |
| --- | --- | --- | --- | --- | --- | --- | --- |
|  |  |  |  | RR (95%CI) | p | RR (95%CI) | p |
| **Hypertension** | | | | | | | |
| Overall | 4300 (80.8) | 4920 (81.2) | 5685 (80.6) | 1.01 (0.99-1.02) | 0.55 | 0.99 (0.98-1.01) | 0.38 |
| Prevalent | 4260 (80.0) | 4875 (80.4) | 5640 (79.9) | 1.01 (0.99-1.02) | 0.55 | 0.99 (0.98-1.01) | 0.47 |
| Treated | 1880 (35.3) | 2205 (36.4) | 2540 (36.0) | 1.03 (0.93-1.03) | 0.34 | 0.99 (0.94-1.040) | 0.65 |
| Missed | 480 (9.0) | 610 (10.1) | 735 (10.4) | 1.12 (1.00-1.25) | 0.06 | 1.04 (0.94-1.15) | 0.51 |
| Incident | 40 (0.8) | 45 (0.7) | 45 (0.6) | 0.99 (0.65-1.51) | 0.96 | 0.86 (0.57-1.30) | 0.47 |
| **Diabetes mellitus** | | | | | | | |
| Overall | 2180 (40.9) | 2480 (40.9) | 2970 (42.1) | 1.00 (0.96-1.05) | 0.99 | 1.03 (0.99-1.07) | 0.17 |
| Prevalent | 2145 (40.3) | 2435 (40.2) | 2915 (41.3) | 1.00 (0.95-1.04) | 0.91 | 1.03 (0.99-1.07) | 0.19 |
| Treated | 1340 (25.2) | 1515 (25.0) | 1875 (26.6) | 0.99 (0.93-1.06) | 0.84 | 1.06 (1.00-1.13) | 0.04 |
| Missed | 150 (2.8) | 205 (3.4) | 230 (3.3) | 1.20 (0.98-1.48) | 0.08 | 0.96 (0.80-1.16) | 0.70 |
| Incident | 35 (0.7) | 45 (0.7) | 55 (0.8) | 1.13 (0.73-1.75) | 0.59 | 1.05 (0.71-1.55) | 0.81 |
| **Hyperlipidemia** | | | | | | | |
| Overall | 2540 (47.7) | 2925 (48.3) | 3495 (49.5) | 1.01 (0.97-1.05) | 0.55 | 1.03 (0.99-1.06) | 0.15 |
| Prevalent | 2520 (47.3) | 2900 (47.9) | 3470 (49.2) | 1.01 (0.97-1.05) | 0.57 | 1.03 (0.99-1.07) | 0.13 |
| Treated | 1890 (35.5) | 2210 (36.5) | 2600 (36.9) | 1.03 (0.98-1.08) | 0.28 | 1.01 (0.97-1.06) | 0.65 |
| Missed | 275 (5.2) | 275 (4.5) | 375 (5.3) | 0.88 (0.75-1.03) | 0.12 | 1.17 (1.01-1.36) | 0.04 |
| Incident | 20 (0.4) | 25 (0.4) | 25 (0.4) | 1.10 (0.61-1.98) | 0.75 | 0.86 (0.49-1.49) | 0.59 |
| **Atrial fibrillation** | | | | | | | |
| Overall | 890 (16.7) | 990 (16.3) | 1200 (17.0) | 0.98 (0.90-1.06) | 0.59 | 1.04 (0.96-1.12) | 0.30 |
| Prevalent | 855 (16.1) | 955 (15.8) | 1140 (16.2) | 0.98 (0.90-1.07) | 0.67 | 1.03 (0.95-1.11) | 0.53 |
| Treated | 400 (7.5) | 440 (7.3) | 590 (8.4) | 0.97 (0.85-1.10) | 0.61 | 1.15 (1.02-1.30) | 0.02 |
| Missed | 265 (5.0) | 300 (5.0) | 310 (4.4) | 1.00 (0.85-1.17) | 0.95 | 0.89 (0.76-1.04) | 0.13 |
| Incident | 35 (0.7) | 35 (0.6) | 60 (0.9) | 0.88 (0.55-1.40) | 0.59 | 1.47 (0.97-2.23) | 0.07 |

Numbers are presented as n (%). RR=risk ratio

**Appendix S Time trends of incident, prevalent, missed, and treated vascular risk factors in England between 2018 and 2024 in Black people aged <55 years**

|  | **2018-2020**  (n=1610) | **2020-2022**  (n=1800) | **2022-2024**  (n=2035) | **2020-2022 vs 2018-2020** | | **2022-2024 vs 2020-2022** | |
| --- | --- | --- | --- | --- | --- | --- | --- |
|  |  |  |  | RR (95% CI) | p | RR (95% CI) | p |
| **Hypertension** | | | | | | | |
| Overall | 1035 (64.3) | 1180 (65.6) | 1280 (62.9) | 1.02 (0.97-1.07) | 0.44 | 0.96 (0.92-1.01) | 0.09 |
| Prevalent | 1015 (63.0) | 1155 (64.2) | 1255 (61.7) | 1.02 (0.97-1.07) | 0.50 | 0.96 (0.92-1.01) | 0.11 |
| Treated | 315 (19.6) | 380 (21.1) | 420 (20.6) | 1.08 (0.94-1.23) | 0.26 | 0.98 (0.86-1.11) | 0.72 |
| Missed | 250 (15.5) | 265 (14.7) | 330 (16.2) | 0.95 (0.81-1.11) | 0.51 | 1.10 (0.95-1.28) | 0.20 |
| Incident | 20 (1.2) | 25 (1.4) | 25 (1.2) | 1.12 (0.62-2.01) | 0.71 | 0.89 (0.51-1.53) | 0.66 |
| **Diabetes mellitus** | | | | | | | |
| Overall | 350 (21.7) | 400 (22.2) | 475 (23.3) | 1.02 (0.90-1.16) | 0.73 | 1.05 (0.93-1.18) | 0.41 |
| Prevalent | 335 (20.8) | 380 (21.1) | 450 (22.1) | 1.02 (0.89-1.16) | 0.83 | 1.05 (0.93-1.18) | 0.45 |
| Treated | 180 (11.2) | 195 (10.8) | 255 (12.5) | 0.97 (0.80-1.17) | 0.75 | 1.16 (0.97-1.38) | 0.10 |
| Missed | 40 (2.5) | 50 (2.8) | 65 (3.2) | 1.12 (0.74-1.69) | 0.59 | 1.15 (0.80-1.65) | 0.45 |
| Incident | 15 (0.9) | 20 (1.1) | 25 (1.2) | 1.19 (0.61-2.32) | 0.60 | 1.11 (0.62-1.98) | 0.74 |
| **Hyperlipidemia** | | | | | | | |
| Overall | 370 (23.0) | 460 (25.6) | 525 (25.8) | 1.11 (0.99-1.25) | 0.08 | 1.01 (0.91-1.12) | 0.86 |
| Prevalent | 360 (22.4) | 450 (25.0) | 515 (25.3) | 1.12 (0.99-1.26) | 0.07 | 1.01 (0.91-1.13) | 0.83 |
| Treated | 240 (14.9) | 310 (17.2) | 345 (17.0) | 1.16 (0.99-1.35) | 0.07 | 0.98 (0.86-1.13) | 0.83 |
| Missed | 80 (5.0) | 85 (4.7) | 115 (5.7) | 0.95 (0.71-1.28) | 0.74 | 1.20 (0.91-1.57) | 0.20 |
| Incident | 10 (0.6) | 10 (0.6) | 10 (0.5) | 0.89 (0.37-2.14) | 0.80 | 0.89 (0.37-2.12) | 0.78 |
| **Atrial fibrillation** | | | | | | | |
| Overall | 100 (6.2) | 130 (7.2) | 150 (7.4) | 1.16 (0.90-1.50) | 0.24 | 1.02 (0.81-1.28) | 0.86 |
| Prevalent | 90 (5.6) | 120 (6.7) | 140 (6.9) | 1.19 (0.92-1.55) | 0.19 | 1.03 (0.82-1.31) | 0.79 |
| Treated | 55 (3.4) | 65 (3.6) | 75 (3.7) | 1.06 (0.74-1.50) | 0.76 | 1.02 (0.74-1.41) | 0.90 |
| Missed | 30 (1.9) | 45 (2.5) | 40 (2.0) | 1.34 (0.85-2.12) | 0.21 | 0.79 (0.52-1.20) | 0.26 |
| Incident | 10 (0.6) | 10 (0.6) | 10 (0.5) | 0.89 (0.37-2.14) | 0.80 | 0.89 (0.37-2.12) | 0.78 |

Numbers are presented as n (%). RR=risk ratio

**Appendix T Time trends of incident, prevalent, missed, and treated vascular risk factors in England between 2018 and 2024 in Asian people aged <55 years**

|  | **2018-2020**  (n=2465) | **2020-2022**  (n=3065) | **2022-2024**  (n=3430) | **2020-2022 vs 2018-2020** | | **2022-2024 vs 2020-2022** | |
| --- | --- | --- | --- | --- | --- | --- | --- |
|  |  |  |  | RR (95% CI) | p | RR (95% CI) | p |
| **Hypertension** | | | | | | | |
| Overall | 1350 (54.8) | 1705 (55.6) | 1895 (55.2) | 1.02 (0.97-1.07) | 0.52 | 0.99 (0.95-1.04) | 0.76 |
| Prevalent | 1320 (53.5) | 1665 (54.3) | 1855 (54.1) | 1.01 (0.97-1.07) | 0.57 | 1.00 (0.95-1.04) | 0.85 |
| Treated | 605 (24.5) | 720 (23.5) | 875 (25.5) | 0.96 (0.87-1.05) | 0.36 | 1.09 (1.00-1.18) | 0.06 |
| Missed | 305 (12.4) | 445 (14.5) | 465 (13.6) | 1.17 (1.02-1.34) | 0.02 | 0.93 (0.83-1.05) | 0.27 |
| Incident | 30 (1.2) | 40 (1.3) | 40 (1.2) | 1.07 (0.67-1.72) | 0.77 | 0.89 (0.58-1.38) | 0.61 |
| **Diabetes mellitus** | | | | | | | |
| Overall | 745 (30.2) | 960 (31.3) | 1075 (31.3) | 1.04 (0.96-1.12) | 0.38 | 1.00 (0.93-1.08) | 0.99 |
| Prevalent | 730 (29.6) | 935 (30.5) | 1045 (30.5) | 1.03 (0.95-1.12) | 0.47 | 1.00 (0.93-1.08) | 0.97 |
| Treated | 470 (19.1) | 575 (18.8) | 675 (19.7) | 0.98 (0.88-1.10) | 0.77 | 1.05 (0.95-1.16) | 0.35 |
| Missed | 75 (3.0) | 125 (4.1) | 140 (4.1) | 1.34 (1.01-1.78) | 0.04 | 1.00 (0.80-1.27) | 1.00 |
| Incident | 15 (0.6) | 25 (0.8) | 30 (0.9) | 1.34 (0.71-2.54) | 0.37 | 1.07 (0.63-1.82) | 0.80 |
| **Hyperlipidemia** | | | | | | | |
| Overall | 900 (36.5) | 1095 (35.7) | 1295 (37.8) | 0.98 (0.91-1.05) | 0.55 | 1.06 (0.99-1.13) | 0.09 |
| Prevalent | 885 (35.9) | 1085 (35.4) | 1280 (37.3) | 0.99 (0.92-1.06) | 0.70 | 1.05 (0.99-1.12) | 0.11 |
| Treated | 650 (26.4) | 800 (26.1) | 955 (27.8) | 0.99 (0.91-1.08) | 0.82 | 1.07 (0.98-1.16) | 0.12 |
| Missed | 145 (5.9) | 180 (5.9) | 225 (6.6) | 1.00 (0.81-1.23) | 0.99 | 1.12 (0.92-1.35) | 0.25 |
| Incident | 15 (0.6) | 10 (0.3) | 15 (0.4) | 0.54 (0.24-1.19) | 0.12 | 1.34 (0.60-2.98) | 0.47 |
| **Atrial fibrillation** | | | | | | | |
| Overall | 155 (6.3) | 175 (5.7) | 190 (5.5) | 0.91 (0.74-1.12) | 0.37 | 0.97 (0.80-1.18) | 0.77 |
| Prevalent | 145 (5.9) | 165 (5.4) | 180 (5.2) | 0.93 (0.74-1.14) | 0.42 | 0.98 (0.79-1.20) | 0.81 |
| Treated | 90 (3.7) | 90 (2.9) | 100 (2.9) | 0.80 (0.60-1.07) | 0.14 | 0.99 (0.75-1.31) | 0.96 |
| Missed | 45 (1.8) | 60 (2.0) | 55 (1.6) | 1.07 (0.73-1.57) | 0.72 | 0.82 (0.57-1.18) | 0.28 |
| Incident | 10 (0.4) | 10 (0.3) | 10 (0.3) | 0.80 (0.34-1.93) | 0.63 | 0.89 (0.37-2.14) | 0.80 |

Numbers are presented as n (%). RR=risk ratio

**Appendix U Time trends of incident, prevalent, missed, and treated vascular risk factors in England between 2018 and 2024 in White people aged <55 years**

|  | **2018-2020**  (n=21460) | **2020-2022**  (n=22115) | **2022-2024**  (n=22535) | **2020-2022 vs 2018-2020** | | **2022-2024 vs 2020-2022** | |
| --- | --- | --- | --- | --- | --- | --- | --- |
|  |  |  |  | RR (95% CI) | p | RR (95% CI) | p |
| **Hypertension** | | | | | | | |
| Overall | 9090 (42.4) | 9500 (43.0) | 9985 (44.3) | 1.01 (0.99-1.04) | 0.21 | 1.03 (1.01-1.05) | 0.004 |
| Prevalent | 8810 (41.1) | 9235 (41.8) | 9665 (42.9) | 1.02 (1.00-1.04) | 0.14 | 1.03 (1.01-1.05) | 0.02 |
| Treated | 3825 (17.8) | 4025 (18.2) | 4455 (19.8) | 1.02 (0.98-1.06) | 0.31 | 1.09 (1.05-1.13) | <0.001 |
| Missed | 2170 (10.1) | 2390 (10.8) | 2375 (10.5) | 1.07 (1.01-1.13) | 0.02 | 0.98 (0.92-1.03) | 0.36 |
| Incident | 280 (1.3) | 265 (1.2) | 320 (1.4) | 0.92 (0.78-1.09) | 0.32 | 1.19 (1.01-1.39) | 0.04 |
| **Diabetes mellitus** | | | | | | | |
| Overall | 3070 (14.3) | 3335 (15.1) | 3585 (15.9) | 1.05 (1.01-1.10) | 0.02 | 1.06 (1.01-1.10) | 0.02 |
| Prevalent | 2985 (13.9) | 3260 (14.7) | 3485 (15.5) | 1.06 (1.01-1.11) | 0.01 | 1.05 (1.00-1.10) | 0.03 |
| Treated | 1925 (9.0) | 2100 (9.5) | 2340 (10.4) | 1.06 (1.00-1.12) | 0.06 | 1.09 (1.03-1.16) | 0.002 |
| Missed | 315 (1.5) | 395 (1.8) | 400 (1.8) | 1.22 (1.05-1.41) | 0.009 | 0.99 (0.87-1.14) | 0.93 |
| Incident | 85 (0.4) | 75 (0.3) | 100 (0.4) | 0.86 (0.63-1.17) | 0.33 | 1.31 (0.97-1.76) | 0.08 |
| **Hyperlipidemia** | | | | | | | |
| Overall | 5565 (25.9) | 6055 (27.4) | 6570 (29.2) | 1.06 (1.02-1.09) | <0.001 | 1.07 (1.03-1.10) | <0.001 |
| Prevalent | 5495 (25.6) | 5980 (27.0) | 6485 (28.8) | 1.06 (1.02-1.09) | <0.001 | 1.06 (1.03-1.10) | <0.001 |
| Treated | 4135 (19.3) | 4575 (20.7) | 4985 (22.1) | 1.07 (1.03-1.12) | <0.001 | 1.07 (1.03-1.11) | <0.001 |
| Missed | 965 (4.5) | 1020 (4.6) | 1140 (5.1) | 1.03 (0.94-1.12) | 0.56 | 1.10 (1.01-1.20) | 0.03 |
| Incident | 70 (0.3) | 75 (0.3) | 85 (0.4) | 1.04 (0.75-1.44) | 0.81 | 1.11 (0.82-1.52) | 0.50 |
| **Atrial fibrillation** | | | | | | | |
| Overall | 1725 (8.0) | 1790 (8.1) | 1915 (8.5) | 1.01 (0.95-1.07) | 0.83 | 1.05 (0.99-1.12) | 0.12 |
| Prevalent | 1630 (7.6) | 1725 (7.8) | 1825 (8.1) | 1.03 (0.96-1.10) | 0.42 | 1.04 (0.98-1.11) | 0.24 |
| Treated | 905 (4.2) | 990 (4.5) | 1020 (4.5) | 1.06 (0.97-1.16) | 0.18 | 1.01 (0.93-1.10) | 0.80 |
| Missed | 460 (2.1) | 415 (1.9) | 450 (2.0) | 0.88 (0.77-1.00) | 0.047 | 1.06 (0.93-1.21) | 0.36 |
| Incident | 95 (0.4) | 65 (0.3) | 90 (0.4) | 0.66 (0.48-0.91) | 0.01 | 1.36 (0.99-1.87) | 0.06 |

Numbers are presented as n (%). RR=risk ratio

**Appendix V Prevalence of patients on relevant treatment for known modifiable stroke risk factors stratified by ethnic groups and time periods** (all p<0.001)

A. **All patients**

Treatment initiation (%)

|  | Blacks | White | Asians |
| --- | --- | --- | --- |
| Hypertension 2018-2020 | 44.1 | 47.9 | 54.6 |
| Hypertension 2020-2022 | 45.2 | 48.2 | 54.1 |
| Hypertension 2022-2024 | 45 | 49.4 | 55.7 |
| Diabetes 2018-2020 | 62.5 | 60.4 | 70.1 |
| Diabetes 2020-2022 | 62.2 | 61.4 | 70.4 |
| Diabetes 2022-2024 | 64.3 | 63.5 | 72.2 |
| Hyperlipidaemia 2018-2020 | 75 | 80.9 | 80.3 |
| Hyperlipidaemia 2020-2022 | 76.2 | 82.1 | 82.6 |
| Hyperlipidaemia 2022-2024 | 74.9 | 83 | 82.9 |
| Atrial fibrillation 2018-2020 | 46.8 | 49.1 | 45.6 |
| Atrial fibrillation 2020-2022 | 46.1 | 51.9 | 45.9 |
| Atrial fibrillation 2022-2024 | 51.8 | 54.7 | 48.1 |

B. **Age<55 years**

Treatment initiation (%)

|  | Blacks | White | Asians |
| --- | --- | --- | --- |
| Hypertension 2018-2020 | 31.0345 | 43.4166 | 45.8333 |
| Hypertension 2020-2022 | 32.9004 | 43.5842 | 43.2432 |
| Hypertension 2022-2024 | 33.4661 | 46.0942 | 47.1698 |
| Diabetes 2018-2020 | 53.7313 | 64.4891 | 64.3836 |
| Diabetes 2020-2022 | 51.3158 | 64.4172 | 61.4973 |
| Diabetes 2022-2024 | 56.6667 | 67.1449 | 64.5933 |
| Hyperlipidaemia 2018-2020 | 66.6667 | 75.2502 | 73.4463 |
| Hyperlipidaemia 2020-2022 | 68.8889 | 76.505 | 73.7327 |
| Hyperlipidaemia 2022-2024 | 66.9903 | 76.8697 | 74.6094 |
| Atrial fibrillation 2018-2020 | 61.1111 | 55.5215 | 62.069 |
| Atrial fibrillation 2020-2022 | 54.1667 | 57.3913 | 54.5455 |
| Atrial fibrillation 2022-2024 | 53.5714 | 55.8904 | 55.5556 |
