# Supplementary material for: Time trends in stroke incidence and modifiable risk factor prevalence in England (2018–2024): A record linkage population-based study
Source: PLoS Med. 2026 Aug 7;23(8):e1004999. doi: 10.1371/journal.pmed.1004999 (PMC13485125; doi:10.1371/journal.pmed.1004999)
Supplement: Supplementary file 3 [file pmed.1004999.s005.docx]

**MS reference number: PMEDICINE-D-26-00739R1**

**Recent trends in stroke incidence and risk factor control in England (2018-2024): a record linkage population-based study**

*We thank the editor and the reviewers for their very helpful comments. We have provided below a point-by-point response to the points raised.*

===========================================
**Response to the editor**

===========================================

**1. Please provide justification regarding the advance offered by your study, compared to the existing literature.**

*Response: Thank you for this suggestion.*

*We have clarified the justification for the current study in the Introduction and also in the Discussion.*

*Briefly, we and others previously showed that there was an age-specific divergence in stroke incidence until 2018 but there has been no data since. This current study provides important data looking at what is happening to strokes at younger vs. older ages since 2018, especially during and after the pandemic. Moreover, giving ongoing monitoring remains crucial, we also showed that linkage with overlapping health system data is feasible and can offer potentially more timely monitoring of trends in disease incidence. Finally, our results highlight that research into the best method to reduce missed opportunities for primary prevention, especially for younger patients and for ethnic minority groups, is urgently needed.*

*We have incorporated the above points in the manuscript. It now reads:*

*Introduction (page 4, first and last paragraph):*

*“Although stroke incidence has decreased by about 42% in high-income countries over the last four decades, this is predominantly driven by a decline at older ages [1-2]. In younger people (under 55 years), there has been a doubling in stroke incidence over the last 10 years in the UK, and a consistent age-specific divergence in all other high-income countries, with a less favourable trend at younger ages [2,4]. However, available data only looked time trends until 2018 and little is known about the ongoing trend, especially in more recent periods, during and after the COVID-19 pandemic.”*

*“We aimed to determine the age-specific changes in stroke incidence since 2018, particularly if there was any impact related to the COVID-19 pandemic, and to assess whether changes in the management of modifiable risk factors coincided with age-specific divergence in stroke incidence in England.”*

*Discussion*

*(page 10, second paragraph):*

*“Our data adds to existing literature on time trends in stroke incidence by age. We previously showed that stroke incidence almost doubled between 2002 and 2018 in Oxfordshire, UK [2]. The absolute increase of approximately 10% between 2018 and 2024 in the current study may appear to be slower than expected but might still be an underestimation due to possible under-ascertainment between 2020 and 2022 as patients with less severe symptoms were less likely to seek medical attention for stroke symptoms during the pandemic [14].”*

*(page 11, middle paragraph):*

*“Our study also has other important policy and research implications. Whilst disease specific population-based studies are still invaluable, they are costly and resource intensive. In contrast, we showed that linkage with overlapping health system data is feasible and can offer potentially more timely monitoring of ongoing trends in disease incidence.”*

===========================================
**Response to the reviewers’ comments**
===========================================

**Reviewer #1 Comments:**

**1. This is an interesting methodological and clinically relevant paper addressing stroke risk pre, per and post the covid pandemic. This means the time window is necessarily relatively small when looking at trends in incidence especially in subgroups but the findings are relevant to policy, service delivery as well as to populations at most risk. There are a few points the authors should consider in the paper if possible.**

*Response: Thank you for this nice summary.*

**2. As these are either routine data sets or audits can the authors further comment on reliability of the diagnosis of first stroke (particularly in primary care records and death certificates), ethnic group and stroke severity if not admitted.**

*Response: Thank you. As we mentioned in the Discussion, previous work from us and others showed that when using linked record in the UK, data sources were 30% hospital admission only, 39% primary care only, 28% hospital and primary care, and 3% death records only. PPVs were 79% for any stroke (89% for hospital admission codes, 80% for primary care codes and 83% for ischemic stroke. The sensitivity is about 70-80% (European Stroke Journal*. 2020;5:26-35*). We have added this point as a limitation in the Discussion:*

*“Finally, our analyses were based on record-linkage of health system data, which relies on the accuracy of diagnostic coding. Although studies showed that, compared to chart review, the positive predictive value was reasonable for identifying any stroke (89% for hospital admission codes, 80% for primary care codes) [30,33], the overall sensitivity was only 70% for hospitalised stroke [30], and further approaches to improve coding accuracy are still required. Moreover, data assessing the timeliness of stroke recording by various sources are also important for ongoing monitoring.”*

*As for stroke severity, we were only able to assess the NIHSS if patients were admitted to the stroke unit – i.e. if they were captured by SSNAP, the national audit. We have also included this as a limitation (page 12, top part of the paragraph):*

*“Thirdly, stroke severity as measured by the NIHSS was only available for a subset of patients who were admitted primarily to acute stroke units. Therefore, we were not able to determine if there were changes in stroke severity more generally during the study periods.”*

*As for ethnicity, recent data (*[*https://pmc.ncbi.nlm.nih.gov/articles/PMC11864522/*](https://pmc.ncbi.nlm.nih.gov/articles/PMC11864522/)*) showed that that the accuracy was overall >95%: 98% for White, 92% for Asians and 90% for Blacks. We also added as a limitation (page 12, middle paragraph):*

*“There was also a very small percentage of patients without any data on ethnicity (<1%) or with potential misclassification (<5%).”*

**3. It would be interesting to illustrate the overlap in data sources with regard to capture and which are the most likely to be useful for further such studies.**

*Response: Thank you. We did not particularly include this data for the current paper as there is a separate paper focusing on this question, which is currently under review: (*[*https://www.medrxiv.org/content/10.64898/2025.12.03.25340732v2*](https://www.medrxiv.org/content/10.64898/2025.12.03.25340732v2)*)*

*Overall we felt that a linked approach would be important. In the paper mentioned above, stroke ascertainment was highest when combining all sources, with 10.8% of non-fatal ischaemic strokes recorded exclusively in primary care and 19.4% of fatal strokes identified solely through death records. Previous work from UKB also showed similar estimates with 30% hospital admission only, 39% primary care only, 28% hospital and primary care, and 3% death records only (Neurology 2020. 95: e607-707)*

*In the current paper, as shown in web appendix 5, changes in patient behaviour and admission policy during the pandemic affected the apparent changes in stroke incidence if only a single source was used. For example, there was a sharp apparent decrease in numbers of strokes recorded by SSNAP and HES during the pandemic due to the fact that many stroke patients were managed as outpatient or in the community without stroke unit admission. We added this element in the Discussion:*

*“The combination of primary care data with hospital episodes is particularly important for the assessment of a condition like acute stroke, when a significant proportion of cases are not hospitalised in the UK [30]. Compared with a single source, linkage with multiple sources also helped to minimise bias related to changes in patient behaviour. This also highlights support for the ongoing effort, policy and initiatives from funding agencies to improve electronic data collection and integration between various data sources.”*

**4. Is there any further exploration of the interaction of ethnicity with deprivation indices?**

*Response: Thank you for this very important point. Unfortunately, we did not have any population level data stratified by age, ethnicity and deprivation for the denominators. We have added this point as a limitation. It now reads (page 12, first paragraph):*

*“Fourth, age and sex-specific population data were not available for smaller strata by ethnicity and region, or by ethnicity and deprivation, hence we were not able to provide more detailed analyses looking at any synergistic effect with these metrics.”*

**5. Minor point-in abstract conclusion the authors talk about risk factor screening, yet there is no screening programme per se but case finding through optimising contacts in health settings and responses for health checks.**

*Response: Thank you. We have now changed “screening” to “identification” to make it clear that we are not referring to health screening or health checks particularly. It now reads (Abstract/page 2):*

*“There was an increase in stroke incidence at younger ages, with no increase at older ages, especially for ethnic minority groups, during and after the COVID-19 pandemic. This coincided with worsening in risk factor identification and diagnosis in primary care, highlighting missed opportunities that need to be urgently addressed.”*

**6. The identification of younger people from ethnic minority groups at high risk is important yet how to identify these groups and improve diagnostic and treatment rates of vascular risk factors is really unsolved and requires some discussion on approaches.**

*Response: Thank you for this important point. We have now added some discussion around possible approaches. It now reads (page 11, second paragraph):*

*“This suggests that whilst recovery of management of vascular risk factors, irrespective of ethnicity, is still ongoing, targeted intervention might be needed to narrow the long-standing inequalities by ethnicity.This is likely to require targeted public health education to enhance health literacy, the use of culturally sensitive screening strategies to improve uptake of risk factor screening and adherence to treatment, and greater community engagement.”*

**Reviewer #2 Comments:**

1. **Thanks for the opportunity to read your manuscript. My role is statistical reviewer, so I have focused on the design, data, and analysis that are presented. I have put general comments first, followed by questions relevant to a specific section of the manuscript (with a page/paragraph reference).

   This manuscript describes incidence of stroke and prevalence of risk factors for stroke in England from 2018 and 2024, with a focus on younger age groups. The study uses linked NHS health data that includes GP visits, hospital admissions, death records, and stroke registry data. Events were included if they occurred between April 2018 and March 2028, and there was no previous stroke recorded in the data. Information on the stroke, as well as patient information, comorbidities, and medications used was also derived from linked data. Fatal stroke was defined as any death within 30 days of a recorded stroke. Risk factors were considered 'prevalent' if they were recorded at least 30 days before the stroke. The study period was divided into pre-COVID, COVID, and post-COVID. Incidence rates were standardised to the 2011 population of England and Wales. Incident rate ratios are reported relative to the 'COVID' study period, and are stratified by age, sex, ethnicity, area measure of
   deprivation, regions, stroke sub-types and data source. Poisson regression was used to estimate IRRs.There was a higher incidence rate of stroke in 2022-2024 compared to 2020-2022, this was consistent across nearly all of the stratification groups in both of the age-groups.

   The rationale for the time-periods is explained well - but would it also be possible to see overall (and by the two age-groups) incidence rates per year across the study period?**

*Response: Thank you for this suggestion and we have now added the results per year across the study period. The results were consistent with the time-periods analysis that the main change happened perhaps around 2022 after the pandemic and remained thereafter.*

*Web appendix 4 Age-standardised incidence (per 1,000) in England by each financial year between 2018 and 2024*

|  | **Overall**  **Incidence (95%CI)** | **<55 years**  **Incidence (95%CI)** | **≥55 years**  **Incidence (95%CI)** |
| --- | --- | --- | --- |
| **2018-2019** | 2.15 (2.13-2.16) | 0.47 (0.46-0.48) | 5.16 (5.12-5.19) |
| **2019-2020** | 2.18 (2.16-2.19) | 0.48 (0.47-0.49) | 5.22 (5.19-5.26) |
| **2020-2021** | 2.10 (2.09-2.11) | 0.49 (0.48-0.50) | 5.00 (4.97-5.03) |
| **2021-2022** | 2.24 (2.22-2.25) | 0.52 (0.51-0.53) | 5.31 (5.28-5.35) |
| **2022-2023** | 2.25 (2.23-2.26) | 0.53 (0.52-0.53) | 5.34 (5.30-5.37) |
| **2023-2024** | 2.25 (2.24-2.26) | 0.53 (0.52-0.54) | 5.33 (5.30-5.36) |

We have added the above both under Methods and also under Results. It now reads:

Methods – statistical analysis (page 6): “*Poisson regression models, adjusted for the population age (18-34, 35-44, 45-54, 54-64, 65-74, 75-84, ≥85) and sex structures, were used to calculate incidence rate ratios (IRRs) for 2020-2022 vs. 2018-2020 and for 2022-2024 vs. 2020-2022. We also added an additional post-hoc analysis reporting the overall and the age-stratified incidence for each financial year per reviewers’ request.”*

Results – page 7: “*Results were consistent in analyses by each financial year (web appendix 4).”*

**2. P4, Paragraph 2. What is the extent of the datasets for determining strokes that occur before 2018? i.e. what is the look-back period?**

*Response: Thank you for this question. As we had access to GDPPR, which included the life-long comprehensive primary care records of the patients, we were able to link diagnostic codes since the patients were registered with GDPPR, which is usually from birth. We have now clarified this under Methods (page 5, top paragraph): it now reads:*

*“Patients were excluded if they had stroke recorded before 1st April 2018 in any source in their lifetime …”*

**3. P5, Paragraph 3. Were the Poisson regression models checked for overdispersion?**

*Response: Thank you for this question. We used direct standardisation for the incident rates and the IRRs were calculated as previously reported in time trends studies in stroke incidence (Lancet 2004;363:1925-33; JAMA 2022;328:563-574). In light of the reviewer’s comments, we have also run an additional analysis taking overdispersion into account. The results are listed below in comparison with current results.*

*We have taken two approaches, Poisson regression models with robust standard errors were used to estimate age-adjusted incidence rate ratios (IRRs), with population denominators included as exposure terms. Although goodness-of-fit statistics suggested deviation from strict Poisson assumptions, negative binomial sensitivity analyses produced similar estimates and indicated minimal overdispersion (α = 0.0004).*

*We haven’t included these new results in the paper, as the current way of calculating standardised IR and IRR was more commonly used but we would be very happy to be guided by the editors and include them as additional analyses if the editor and the reviewer feel it is required.*

| *IRR (95%CI)* | ***Current results*** | ***Robust SE*** | ***Negative binomial*** |
| --- | --- | --- | --- |
| ***All*** |  |  |  |
| *IRR (2020-2022 vs. 2018-2020)* | 1.00 (1.00-1.01) | 1.00 (0.98-1.03) | 1.02 (1.00-1.05) |
| *IRR (2022-2024 vs. 2020-2022)* | 1.04 (1.03-1.04) | 1.04 (1.02-1.06) | 1.04 (1.02-1.06) |
| ***Age<55 years*** |  |  |  |
| *IRR (2020-2022 vs. 2018-2020)* | 1.07 (1.05-1.09) | 1.07 (1.06-1.07) | 1.07 (1.05-1.08) |
| IRR (2022-2024 vs. 2020-2022) | 1.04 (1.02-1.06) | 1.05 (1.04-1.05) | 1.05 (1.03-1.06) |
| ***Age≥55 years*** |  |  |  |
| *IRR (2020-2022 vs. 2018-2020)* | 0.99 (0.99-1.00) | 0.99 (0.97-1.02) | 1.00 (0.97-1.03) |
| *IRR (2022-2024 vs. 2020-2022)* | 1.04 (1.03-1.04) | 1.03 (1.01-1.05) | 1.03 (1.01-1.06) |

**4. P5, Paragraph 7. I tried to access the repository - but this didn't load. I also couldn't find it listed in the BHFDSC github page.**

*Response: Apologies. There was some admin delay to make the repo public. We can confirm that it is now available with the link.*

**5. P16, Table 1. Is 'per 1,000' per 1,000 person-years?**

*Response: Apologies – it is per 1,000 person-years. We have now clarified it.*

**6. P20, Figure 2. This is a nice figure. Optionally, you could add an explainer to the x-axis saying 'more common in 2020-2022' and 'more common in 2018-2020'**

*Response: Thank you for this suggestion. We have now added an explanation to the figure legends to make it easier for the readers to understand. It now reads:*

*“Each blue diamond represents the relative risk of hypertension, diabetes, dyslipidemia, or atrial fibrillation within the relevant population subgroup. The horizontal tip on each side of the blue diamond represents the 95% CI of the relative risk. The vertical dashed red line represents the line of no difference (risk ratio = 1) between the periods compared (2020-2022 vs 2018-2020 or 2022-2024 vs 2020-2022). Risk ratio (RR)>1 means that the risk factor of interest is more common in the period listed first (i.e., 2020-2022 in the plots of the top row and 2022-2024 in the plots of the bottom row). RR<1 means that the risk factor of interest is less common in the period listed first (i.e., 2020-2022 in the plots of the top row and 2022-2024 in the plots of the bottom row).”*

**Reviewer #3 Comments:**

1. **This article describes trends in English stroke incidence and pharmacological risk factor management, stratified by pre/ during/ post pandemic periods. The paper was clear and well-written and presented. My major query is the rationale for the <55 years age cut-off, which many of the analyses were based on, but didn't seem to be borne out by the descriptive data as far as I could see. Some other minor comments/ suggestions are also outlined below.**

   *Response: Thank you for this important point. We agree that the cut-off for “young” at age<55 might appear to be arbitrary. We used this cut-off as a priori (i.e. not based on the current data) as this is one of the most commonly used cut-off in the literature of strokes at younger ages (DOI: 10.1177/17474930251362583). Previous literate chose this age cut-off as more traditional stroke risk factors and aetiologies begin to emerge after age 55 years. Moreover, this is the cut-off we also used in a previous population-based study and a systematic review which showed for the first time the age-specific divergence (age<55y vs. age>=55y) in stroke incidence in high-income countries (DOI: 10.1177/17474930251362583; DOI: 10.1177/17474930251362583).*

*In light of the reviewer’s comments, we have now added this as a limitation. It now reads (page 12, middle paragraph):*

*”Sixth, we used age<55 years to define “young stroke” based on previous work showing an age-specific divergence comparing age<55 years vs. age≥ 55 years.^2,4^ Although this cut-off is most commonly used in the young stroke literature, it is nevertheless arbitrary.”*

2. **Introduction: Given you have published previously on age-divergent trends in stroke incidence, in the first and last paras of this section the paper would be better framed as an investigation of peri-pandemic trends and risk factors. Or are you using better data sources? If so, you should state this.**

*Response: apologies for not making this point clearer. As the reviewer pointed out, we and others previously showed that there was an age-specific divergence in stroke incidence until 2018. The reasons for this divergence remained unclear, highlighting the importance of ongoing monitoring. However, there has been no recent data since 2018. This current study provides important data looking at what is happening to strokes at younger vs. older ages since 2018, especially during and after the pandemic.*

*We have incorporated the above points in the manuscript. It now reads:*

*Introduction (page 4, first and last paragraph):*

*“Although stroke incidence has decreased by about 42% in high-income countries over the last four decades, this is predominantly driven by a decline at older ages.^1-3^ In younger people (under 55 years), there has been a doubling in stroke incidence over the last 10 years in the UK, and a consistent age-specific divergence in all other high-income countries, with a less favourable trend at younger ages.^2, 4^ However, available data only looked time trends until 2018 and little is known about the ongoing trend, especially in more recent periods, during and after the COVID-19 pandemic.”*

*“We aimed to determine the age-specific changes in stroke incidence since 2018, particularly if there was any impact related to the COVID-19 pandemic, and to assess whether changes in the management of modifiable risk factors coincided with age-specific divergence in stroke incidence in England.”*

3. **You postulate decreased insulin use in people with type 1 diabetes during the pandemic as an explanation for higher stroke incidence in younger people - this doesn't sound likely to me. Were there any data on age trends in statin/ antihypertensive prescriptions over the pre/ during/ post pandemic periods in the paper cited by Dale et al?**

*Response: Apologies for the confusion. In the paper cited by Dale et al, the authors did not report any time trends by age. We used the decreased use in insulin as an indirect pointer to suggest that there might have been changes in the use of preventive treatment by age – as type 1 diabetes are more common at younger ages. We were not intending to associate the decreased use in insulin with the increase in stroke incidence at younger ages. Rather, our hypothesis was that there might be age-specific changes in the use of general preventative treatment (blood pressure lowering/statins/diabetic treatment) during the pandemic, which might be associated with the continued age-specific divergence in stroke incidence. We have clarified the above points in the Introduction. It now reads (page 4, middle paragraph):*

*“Third, particularly during the pandemic, the control of vascular risk has declined, with fewer face-to-ace primary care appointment and prescription of antihypertensive, lipid lowering drugs and anticoagulation for atrial fibrillation [8,9]. Whether these changes differed by age is unclear but the decreased use of insulin - needed mainly in younger people with type 1 diabetes, but not other anti-diabetic medications, suggests that the use of preventative treatment more generally might have differentially affected patients at younger versus older ages [8].”*

4. **Also you haven't mentioned a key driver of CVD risk in younger people -** **rising obesity levels - shown to be worse for young than old people during the pandemic (**[**https://pubmed.ncbi.nlm.nih.gov/38913709/**](https://pubmed.ncbi.nlm.nih.gov/38913709/)**).**

*Response: thank you for this suggestion. We have now added this important point in the Introduction. It now reads (page 4, middle paragraph):*

*“Fourth, rising obesity levels appeared to be worse for younger than older individuals during the pandemic [10].”*

5. **Did you also consider competing risks here (and in the analysis)? COVID-19 had far higher mortality in older people.**

*Response: thank you for this question. As we started with the disease population to calculate incidence rather than from the general population in a prospective cohort to assess risk, we did not consider competing risk. The reviewer is absolutely right that COVID-19 had higher mortality at older ages. The underlying population estimates were taking the mid-point population structure at the given time period, which would have taken into account the population structure change as a result of COVID-19.*

6. **Methods: The text would be clearer if the covariates (sex, ethnicity etc) were described in a separate para to the stroke ascertainment.**

*Response: thank you for this excellent suggestion. We have now added three sub-headings under “Diagnosis” - Stroke; demographics; vascular risk factors, each with a separate paragraph.*

7. **How did you ascertain presence of risk factors and from which sources?**

*Response: thank you and apologies for not being clear. Presence of risk factors were ascertained with pre-defined diagnostic codes (*[*https://github.com/BHFDSC/CCU084_01*](https://github.com/BHFDSC/CCU084_01)*) from linked records including diagnosis either made in primary care (GDPPR) or in hospital (HES and SSNAP). We have now clarified this in the manuscript under Methods. It now reads (page 5, middle paragraph):*

*“We also collected data on the presence and date of diagnosis of hypertension, diabetes, atrial fibrillation and hyperlipidaemia using pre-defined diagnostic codes in primary care (SNOMED CT codes from GDPPR) and in hospital (ICD-10 codes from HES).”*

*We also added the Github link above where the readers can see the codelist and definitions.*

8. **How was the overlap dealt with between prevalent/ incident/ missed versus treated groups (see comment below)?**

*Response: Apologies for the confusion. We include the schematic representation of the definition below. We have also included this in the web appendix. The goal of these analyses was not to build mutually exclusive categories but to report statistics on specific subpopulations of interest. Similarly, we provided incidence for people <55 years old and for people aged 18-34. The two groups are not mutually exclusive and we used these groups to represent different subpopulations of interest.*

*Web appendix 1*

9. **Results: It would be good to see a table of baseline characteristics. Ideally by pandemic period and presence/ absence of stroke so the data structure can be understood at a glance, including missingness for ethnicity etc.**

*Response: Apologies for the confusion. As our study is looking at incident strokes in England, the population is the population with the disease – i.e. it did not include patients without any stroke. The prevalences of risk factors by period, by age, by sex, and by ethnicity are presented in web appendix 10-21.*

*We have now also clarified that participants with missing ethnicity data (n=3244) or those classified as “mixed” (n=4957), or “other” (n=6797) ethnic groups were not included in analyses stratified by ethnicity. It now reads (page 6, bottom paragraph):*

*“… Participants with missing ethnicity data (n=3244) or those classified as “mixed” (n=4957), or “other” (n=6797) ethnic groups were not included in analyses stratified by ethnicity.”*

10. **Why did you choose to stratify age at 55 years? It seems slightly counter-intuitive given that patterns of increasing incidence seems to hold until up to 75 yrs, according to table 1. But perhaps there's a clinical justification for this threshold.**

*Response: Thank you for this important point. We agree that the cut-off for “young” at age<55 might appear to be arbitrary. We used this cut-off as a priori (i.e. not based on the current data) as this is one of the most commonly used cut-off in the literature of strokes at younger ages (DOI: 10.1177/17474930251362583). Previous literate chose this age cut-off as more traditional stroke risk factors and aetiologies begin to emerge after age 55 years. Moreover, this is the cut-off we also used in a previous population-based study and a systematic review which showed for the first time the age-specific divergence (age<55y vs. age>=55y) in stroke incidence in high-income countries (DOI: 10.1177/17474930251362583; DOI: 10.1177/17474930251362583).*

*In light of the reviewer’s comments, we have now added this as a limitation. It now reads (page 12, middle paragraph):*

*”Sixth, we used age<55 years to define “young stroke” based on previous work showing an age-specific divergence comparing age<55 years vs. age≥ 55 years [2,4]. Although this cut-off is most commonly used in the young stroke literature, it is nevertheless arbitrary.”*

11. **For clarity, can you expand "ASI" in the legend of table 2? And reiterate what the RTTRs are comparing in the text of the results section?**

*Response: Thank you. We have now expanded “ASI” for table 2. It now reads in the title:*

*“Table 2 Time trends of age-standardised stroke incidence (per 1,000) in England between 2018 and 2024 stratified by sex, ethnicity, deprivation, stroke subtypes and region in those aged ≥55 years (ASI=Absolute stroke incidence)”*

*We have also reiterated what the RTTRs were comparing under Results. It now reads (page 7):*

*“Consequently, the age divergence in stroke incidence with a less favourable trend at younger ages was greater in 2020-2022 compared to 2018-2020 (incidence at younger vs. older ages between the two periods - RTTR=1.07, 95%CI 1.05-1.08, p<0.001; Figure 1A).”*

12. **In web appendix 8, the overlap between prevalent/ incident/ missed versus treated groups is confusing. For example, in 2018-2020, if 73.5% had prevalent hypertension but only 35.3% (presumably nested within that 73.5%) were treated, were the remaining 38.2% classified as missed? Yet only 7.6% are missed in the table. Or were some of them below treatment thresholds? Clearly people with hypertension requiring treatment who aren't on it will be at higher risk, so it's important to show that in the table somehow. See above comment in the methods section.**

*Response: apologies for not making this clearer. As mentioned in the response to an earlier question, cases with missed diagnosis were those that had the risk factor of interest diagnosed at the time of the stroke suggesting that the condition is most likely already present before the stroke – e.g. new diagnosis of diabetes at the time of the stroke would suggest that the diabetes was most likely already there before the stroke but undiagnosed.*

*We have now clarified the definitions with a further figure listed below (web appendix 1).*

*The reviewer is absolutely right to point out many patients with known risk factors were not on treatment – they are not classified as “missed” as the diagnosis was known. This phenomenon is not uncommon. We and others previously also found that about 50% of the patients with known hypertension and 80% of those with known hyperlipidaemia were not treated before the stroke. This is largely due to a risk score based approach with primary care physicians using a threshold of 10% risk of cardiovascular disease in 10 years to decide whether to offer pharmacological treatment (BMC Med. 2026;24:107; JAMA. 2022;328:563-574). As our current study only had patients with stroke, we were not able to address the associations between treatment status and stroke risk. We have highlighted how this differed by age and ethnicity in the main Results.*

*“…compared with older patients, those aged<55 years with known hypertension were less likely treated prior to stroke, similarly over time (age</≥55 years – 2018-2020 42.2% vs 48.6%; 2020-2022 42.1% vs 49.0%; 2022-2024 44.7% vs 49.9%; all p<0.0001).”*

*“It is also worth noting that compared to White and Asian, Black was less often prescribed any blood pressure lowering drug despite known history of hypertension, which again remained unchanged during the three study periods (2018-2020: Blacks 44.1% vs White 47.9% vs Asians 54.6%; 2020-2022: Blacks 45.2% vs White 48.2% vs Asians 54.1%; 2022-2024: Blacks 45.0% vs. White 49.4% vs. Asians 55.7%; all p<0.0001)”*

*“Similar to hypertension, there were differences in initiation of treatment by age and ethnicity in patients with known diabetes and hyperlipidaemia. Compared with patients ≥55 years, patients <55 years with known hyperlipidaemia were less likely to be treated with lipid-lowering drugs throughout the three study periods (age</≥55 years: 2018-2020 74.3% vs 81.1%; 2020-2022 75.4% vs 82.4%; 2022-2024 75.8% vs 83.2%; all p<0.0001; web appendix 12-1313-14), although there was no difference in initiation of diabetic-related treatment in those with diabetes (age</≥55 year: 2018-2020 62.7% vs 61.3%, p=0.15; 2020-2022 62.6% vs 62.4%, p=0.78; 2022-2024 65.5% vs 64.3%, p=0.78; web appendix 15-16). For ethnic groups, compared to White and Asian, Black was again less often prescribed lipid lowering or diabetic treatment despite known history of diabetes and hyperlipidaemia, which remained unchanged during the three study periods. The ethnic differences were most prominent at age<55 years (web appendix 22).”*

13. **Also did you have any data on risk factor control?**

*Response: thank you for this important point. As mentioned in the Methods and Results, we measured if patients were on preventative treatment for risk factors (e.g. diabetic medication, lipid lowering and blood pressure lowering. For example, we reported that*

*“…compared with older patients, those aged<55 years with known hypertension were less likely treated prior to stroke, similarly over time (age</≥55 years – 2018-2020 42.2% vs 48.6%; 2020-2022 42.1% vs 49.0%; 2022-2024 44.7% vs 49.9%; all p<0.0001).”*

*“It is also worth noting that compared to White and Asian, Black was less often prescribed any blood pressure lowering drug despite known history of hypertension, which again remained unchanged during the three study periods (2018-2020: Blacks 44.1% vs White 47.9% vs Asians 54.6%; 2020-2022: Blacks 45.2% vs White 48.2% vs Asians 54.1%; 2022-2024: Blacks 45.0% vs. White 49.4% vs. Asians 55.7%; all p<0.0001)”*

*“Similar to hypertension, there were differences in initiation of treatment by age and ethnicity in patients with known diabetes and hyperlipidaemia. Compared with patients ≥55 years, patients <55 years with known hyperlipidaemia were less likely to be treated with lipid-lowering drugs throughout the three study periods (age</≥55 years: 2018-2020 74.3% vs 81.1%; 2020-2022 75.4% vs 82.4%; 2022-2024 75.8% vs 83.2%; all p<0.0001; web appendix 12-1313-14), although there was no difference in initiation of diabetic-related treatment in those with diabetes (age</≥55 year: 2018-2020 62.7% vs 61.3%, p=0.15; 2020-2022 62.6% vs 62.4%, p=0.78; 2022-2024 65.5% vs 64.3%, p=0.78; web appendix 15-16). For ethnic groups, compared to White and Asian, Black was again less often prescribed lipid lowering or diabetic treatment despite known history of diabetes and hyperlipidaemia, which remained unchanged during the three study periods. The ethnic differences were most prominent at age<55 years (web appendix 22).”*

*One limitation is that we did not have data on HbA1c, lipid levels or BP levels. We have now added this a limitation under Discussion. It now reads (page 12, middle paragraph):*

*“Seventh, we used presence of preventative medication as a surrogate marker to assess control of risk factors. However, we did not have measurements of blood pressure, lipid level or diabetes control during the study periods.”*

14. **Did you consider adjusting you IRR for the age differences in stroke rate increases for the risk factors? That could enable a firmer conclusion as to the cause of the age/ pandemic period divergences.**

*Response: thank you for this suggestion. As we started with the stroke population, we were not able to assess how stroke incidence varied by different levels of risk factor changes. This would require data on the prevalence of risk factors by age and sex for the underlying population, which we did not have.*

*We have added this as a limitation. It now reads (page 12, middle paragraph):*

*“Eighth, we previously showed that there was similar magnitude of increase in stroke incidence at younger ages in those with vs. without vascular risk factors [2]. As we did not have the age specific risk factor prevalence data in more recent periods in the underlying population, we were not able to determine if the observed changes since 2018 were driven by patients with certain risk factor profiles.”*

15. **Discussion: Here again the spiraling rates of obesity/ type 2 diabetes are only mentioned cursorily, but are likely to be a key driver of strokes in younger people.**

*Response: thank you. We agree that the increasing incidence of obesity and type 2 diabetes are important but we previously showed that they were unlikely the main drivers for the increase in time trends in young stroke (JAMA. 2022;328:563-574). In this previous work, we showed that there was the same magnitude of increase in patients without these risk factors and there was an increase in patients without any vascular risk factors. Moreover, we found a decrease of myocardial infarction at younger ages, where the risk associations with the above risk factors were usually stronger. Whilst obesity and diabetes are certainly increasing in the background population and they are certainly important, there might be some synergistic effect with other emerging risk factors that ultimately contributed to the increase in young stroke. We have incorporated this point in the Discussion.*

*“Changes in patient behaviour or investigations are unlikely to be the main drivers and some synergistic effect of traditional vascular risk factors, such as hypertension, diabetes and obesity, and emerging stroke risk factors, such as stress and pollution, could likely be playing a role [2].”*

16. **You do not mention missing data in your limitations para. Presumably there was missingness for ethnicity, and some of the risk factor data in the SDE (or did you ascertain that from other sources?).**

*Response: apologies – we have now added the missingness for ethnicity as a limitation. There were 3244 participants with missing ethnicity data. As we were also to link three resources (primary care - GDPPR, hospital admission - HES and national stroke audit - SSNAP). It now reads (page 12, middle paragraph):*

*“There was also a very small percentage of patients (<1%) without any data on ethnicity.”*

**Reviewer #4 Comments:**

1. **This manuscript reports a large, methodologically ambitious, and policy-relevant population-based study using linked national data sources to examine age-specific trends in stroke incidence before, during, and after the COVID-19 pandemic. The study addresses an important and timely question and has the potential to make a substantial contribution to the literature. The observation of a persisting increase in stroke incidence among adults aged <55 years, particularly among ethnic minority groups, has important implications for both clinical practice and health policy and is appropriately highlighted by the authors. The manuscript is generally well written, the analyses are coherent, and the conclusions are largely supported by the presented data.**

*Response: Thank you for this nice summary.*

2. **Case ascertainment and pandemic related issues
   The use of multiple overlapping data sources is a major strength; however, the manuscript would benefit from clearer reassurance that changes in healthcare utilisation during the pandemic did not differentially affect stroke ascertainment by age group.
   a.      Could you provide any details on whether the completeness and timeliness of stroke recording (especially in primary care and audit data) varied across the three study periods?
   b.      Younger adults may have been more likely to delay or avoid care during the pandemic; could you discuss how this might bias estimates?**

*Response: thank you for these two important points. As shown in web appendix 5, changes in patient behaviour and admission policy during the pandemic affected the apparent changes in stroke incidence if only a single source was used. For example, there was a sharp apparent decrease in numbers of strokes recorded by SSNAP and HES during the pandemic due to the fact that many stroke patients were managed as outpatient or in the community without stroke unit admission. We added this element in the Discussion (page 11, middle paragraph):*

*“The combination of primary care data with hospital episodes is particularly important for the assessment of a condition like acute stroke, when a significant proportion of cases are not hospitalised in the UK [30]. Compared with a single source, linkage with multiple sources also helped to minimise bias related to changes in patient behaviour. This also highlights support for the ongoing effort, policy and initiatives from funding agencies to improve electronic data collection and integration between various data sources.”*

*We did not have a gold standard to formally compare the timeliness of stroke recording by source and study periods. We have added this as a limitation. It now reads (page 12, last point in limitations):*

*“Finally, our analyses were based on record-linkage of health system data, which relies on the accuracy of diagnostic coding. Although studies showed that, compared to chart review, the positive predictive value was reasonable for identifying any stroke (89% for hospital admission codes, 80% for primary care codes) [30,33], the overall sensitivity was only 70% for hospitalised stroke [30], and further approaches to improve coding accuracy are still required. Moreover, data assessing the timeliness of stroke recording by various sources are also important for ongoing monitoring.”*

*The reviewer is also right that we might have underestimated the incidence of stroke at younger ages during the pandemic due to behaviour changes. We have added this to the Discussion (page 10, second paragraph):*

*“Our data adds to existing literature on time trends in stroke incidence by age. We previously showed that stroke incidence at age <55 years almost doubled between 2002 and 2018 in Oxfordshire, UK [2]. he absolute increase of approximately 10% between 2018 and 2024 in the current study may appear to be slower than expected but might still be an underestimation due to possible under-ascertainment between 2020 and 2022 as younger patients with less severe symptoms were less likely to seek medical attention for stroke symptoms during the pandemic [14.]^”^*

3. **Choice of age threshold (<55 vs ≥55 years)
   The dichotomisation at age 55 seems reasonable and aligns with some prior literature, but it remains somewhat arbitrary. Please justify this cut point.**

*Response: Thank you for this important point. We agree that the cut-off for “young” at age<55 might appear to be arbitrary. We used this cut-off as a priori (i.e. not based on the current data) as this is one of the most commonly used cut-off in the literature of strokes at younger ages (DOI: 10.1177/17474930251362583). Previous literate chose this age cut-off as more traditional stroke risk factors and aetiologies begin to emerge after age 55 years. Moreover, this is the cut-off we also used in a previous population-based study and a systematic review which showed for the first time the age-specific divergence (age<55y vs. age>=55y) in stroke incidence in high-income countries (DOI: 10.1177/17474930251362583; DOI: 10.1177/17474930251362583).*

*In light of the reviewer’s comments, we have now added this as a limitation. It now reads (page 12, middle paragraph):*

*”Sixth, we used age<55 years to define “young stroke” based on previous work showing an age-specific divergence comparing age<55 years vs. age≥ 55 years [2,4]. Although this cut-off is most commonly used in the young stroke literature, it is nevertheless arbitrary.”*

4. **Interpretation of increasing post pandemic incidence
   The increase in stroke incidence at age ≥55 years in the post pandemic period deserves more careful interpretation. Could this reflect delayed presentations or diagnostic backlog rather than a true rise in incidence?**

*Response: we agree that delayed presentations could potentially contribute to an apparent increase in incidence if the increase was brief and short lifting. However, we saw a more sustained effect, which suggested that this unlikely due to delayed presentations or diagnostic backlog. We have now added the additional analysis by each year in the web appendix.*

*Web appendix 4 Age-standardised incidence (per 1,000) in England by each financial year between 2018 and 2024*

|  | **Overall**  **Incidence (95%CI)** | **<55 years**  **Incidence (95%CI)** | **≥55 years**  **Incidence (95%CI)** |
| --- | --- | --- | --- |
| **2018-2019** | 2.15 (2.13-2.16) | 0.47 (0.46-0.48) | 5.16 (5.12-5.19) |
| **2019-2020** | 2.18 (2.16-2.19) | 0.48 (0.47-0.49) | 5.22 (5.19-5.26) |
| **2020-2021** | 2.10 (2.09-2.11) | 0.49 (0.48-0.50) | 5.00 (4.97-5.03) |
| **2021-2022** | 2.24 (2.22-2.25) | 0.52 (0.51-0.53) | 5.31 (5.28-5.35) |
| **2022-2023** | 2.25 (2.23-2.26) | 0.53 (0.52-0.53) | 5.34 (5.30-5.37) |
| **2023-2024** | 2.25 (2.24-2.26) | 0.53 (0.52-0.54) | 5.33 (5.30-5.36) |

*Having said that, the reviewer is absolutely right that patients with minor symptoms might not have presented at all during the pandemic hence there could be an underestimation of incidence during the pandemic resulting in an apparent “increase” after the pandemic. But we saw the same increase for ICH, for which patients would almost always present due to the severity of the symptoms (table 3). Moreover, the incidence in 2022, 2023 and 2023 was also higher than 2018 and 2019, which was before the pandemic.*

5. **Undiagnosed risk factors
   The finding of increased "undiagnosed" hypertension and diabetes in younger adults is important but requires clearer methodological explanation. How exactly were "undiagnosed" cases defined? Is it possible that reduced primary care contact led to delayed coding rather than truly missed detection?**

*Response: “undiagnosed” cases were those with potentially false new diagnosis: among those with prevalent risk factors, if the diagnosis was absent before the index event but made at the time of the index stroke or within 30 days thereafter. Risk factors were considered prevalent if the patient received the diagnosis or the relevant treatment before or within 30 days of the index stroke.*

*We think the “underdiagnosis” scenario is genuine as missed opportunistic screening has been a concern in primary care since the pandemic. Moreover as we looked at all diagnostic codes and medication dispensing data before the stroke to ascertain the status of these risk factors., the missing diagnosis is unlikely related to delayed coding. As diagnosis and treatment are usually initiated by primary care, even there could be a brief delay in recording the risk factor itself by a couple of days, it shouldn’t have a big gap and the treatment records should still reflect the diagnosis.*

6. **Stroke subtype analyses: Specify whether ANY trends differed meaningfully between ischaemic and haemorrhagic stroke.**

*Response: thank you and as reported in the paper, there was no difference in trends for ischaemic vs. haemorrhagic stroke.*

*“… These overall trends were consistent by sex, deprivation, region and were also seen for ischaemic stroke, ICH and unknown stroke. Age standardized incidence for SAH was stable between 2020-2022 versus 2018-2020 and declined by 4% in 2022-2024.”*

7. **Terminology and clarity
   a.      I would suggest providing an explanation of the Relative Temporal Rate Ratio (RTTR) in the Methods, as this metric may be unfamiliar to some readers.
   b.      The sentence "Research into the best method to reduce missed opportunities for primary prevention, especially for younger patients" in the Discussion sounds unclear to me.**

*Response: apologies for the typo. This sentence should be*

*“Research into the best method to reduce missed opportunities for primary prevention is needed, especially for younger patients.”*

As for RTTR, we have now also expanded the section to explain how it was calculated. It now reads (page 6, middle paragraph):

“*To measure the age-specific divergence in IRR, the relative temporal difference (Relative Temporal Rate Ratio- RTTR) was calculated by dividing the IRR at younger than 55 years by the IRR at age 55 years or older (web appendix 2).*^4^”

*Web appendix 2 Calculation and interpretation of the relative temporal trend ratio (RTTR)*

A. **Where crude numbers are available for each time period**

Where:	*_y_n_p0_*= number of new cases stroke age<55 years during earliest time period (p_0_)

*_y_N_p0_*= population of those age <55 years ×time during which cases were ascertained during p_0_

*_y_n_p1_*= number of new cases stroke age<55 years during latest time period (p_1_)

*_y_N_p1_*= population of those age <55 years ×time during which cases were ascertained during p_1_

*_o_n_p0_*= number of new cases stroke age ≥55 years during p_0_

*_o_N_p0_*= population of those age ≥55 years ×time during which cases were ascertained during p_0_

*_o_n_p1_*= number of new cases stroke age ≥55 years during p_1_

*_o_N_p1_*= population of those age ≥55 years ×time during which cases were ascertained during p_1_

IR=incidence rate; IRR=incidence rate ratio

1. IR for those aged under 55 years (_y_IR) during earliest time period (p_0_):

$${yIR}_{p0}=\frac{{yn}_{p0}}{{yN}_{p0}}$$

2. IR for those aged 55 years or older (_o_IR) during earliest time period (p_0_):

$${oIR}_{p0}=\frac{on_{p0}}{{oN}_{p0}}$$

3. IR for those aged under 55 years (_y_IR) during latest time period (p_1_):

$${yIR}_{p1}=\frac{{yn}_{p1}}{{yN}_{p1}}$$

4. IR for those aged 55 years or older (_o_IR) during latest time period (p_1_):

$${oIR}_{p1}=\frac{{on}_{p1}}{{oN}_{p1}}$$

5. IRR for those aged under 55 years (_y_IRR):

$$yIRR=\frac{{yIR}_{p1}}{{yIR}_{p0}}$$

6. IRR for those aged 55 years or older (_o_IRR):

$$oIRR=\frac{{oIR}_{p1}}{{oIR}_{p0}}$$

7. Relative temporal trend ratio (RTTR):

$$RTTR=\frac{\mathrm{yIRR}}{oIRR}$$

Uncertainty about the estimate of the RTTR due to chance were estimated by calculating the standard error (s.e) of the log (RTTR) as follows:

$$s.e.(logRTTR)=\sqrt{\frac{1}{{yn}_{p0}}-\frac{1}{{yN}_{p0}}+\frac{1}{{on}_{p0}}-\frac{1}{{oN}_{p0}}+\frac{1}{{yn}_{p1}}-\frac{1}{{yN}_{p1}}+\frac{1}{{on}_{p1}}-\frac{1}{{oN}_{p1}}}$$

B. **Where raw numbers were not reported but age specific IRR and 95% CI were reported:**

IRR and 95% CI were used to estimate SE as follows:

$$s.e.\left( IRR \right)=\frac{\left( \frac{ln (95\% upper limit of IRR) - ln(IRR)}{1.96} \right)+\left( \frac{ln(IRR) -ln (95\% lower limit of IRR)}{1.96} \right)}{2}$$

$$s.e.\left( RTTR \right)=\sqrt{{s.e(yIRR)}^{2}+{s.e(oIRR)}^{2}}$$

**Interpretation of RTTR**

The relative temporal trend ratio (RTTR) is a comparative measure to identify and quantify divergence in stroke incidence trends between younger and older people within a study. The RTTR is calculated from age-specific temporal incidence rate ratios (IRR), as demonstrated in the formula above. Here, we define IRR as the incidence rate in the latest reported time period divided by the incidence rate in the earliest reported time period. The RTTR is then calculated by dividing the IRR at younger ages by the IRR at older ages. The RTTR can therefore help identify age-specific differences in incidence trends which may be masked by overall incidence trends especially in those studies where the IRR at younger ages was also declining. While the RTTR is cruder than comparisons of age-specific average annual percent change (AAPC) in incidence, it allows a single summary comparison of age-specific incidence trends which can be measured in different studies reporting data in different age groups, at different time periods, including those which only reported at 2 time periods, and can ultimately be meta-analysed across studies.

The RTTR can be interpreted as follows:

- RTTR less than 1 would indicate that stroke incidence trends at younger ages were more favourable than stroke incidence trends at older ages.
- RTTR of 1 would indicate the same trends in stroke incidence were seen at younger and older ages.
- RTTR of greater than 1 would indicate that the stroke incidence trends at younger ages were less favourable than at older ages.

As the RTTR is a ratio of two measures, additional contextual information is required to interpret differences in the RTTR; for instance a RTTR of less than 1 could be driven either by a decrease in stroke incidence at younger ages or an increase in stroke incidence at older ages, or a combination of both to differing degrees. Two particular issues should also be understood:

1. The magnitude (but not the direction) of the RTTR will depend to some degree on the duration of the time period studied; therefore the RTTR should be interpreted with caution when comparing studies with very different durations. In a sensitivity analysis, we therefore attempted to standardise the RTTR to a 10-year study duration.

2. The RTTR can also be impacted by age-specific changes in population structure over time or any age-specific changes in completeness of case-ascertainment. For example, estimates of RTTR were based only on the crude incidence of stroke in each of the two age groups and would not therefore fully adjust for the ageing of the underlying study population over time. Any fall in age-specific stroke incidence in the older age group would be underestimated by the crude incidence rate due to the continuing population aging that will have occurred during these studies in high-income countries. Since this same bias would not be seen in the younger age group, the RTTR will tend to underestimate divergence.

8. **References
   Please use full metrics for Refs #15, #19, #26, #27:
   #15: PLoS Med. 2024;21(11):****e1004485
   #19: Eur Heart J. 2025;46(42):****4359-4378
   #26: Nat Commun. 2025;****16(1):6059.
   #27: EClinicalMedicine. 2023;61:****102077**

*Response: apologies and we have now used full metrics for the mentioned references.*
