# Supplementary material for: Time trends in stroke incidence and modifiable risk factor prevalence in England (2018–2024): A record linkage population-based study
Source: PLoS Med. 2026 Aug 7;23(8):e1004999. doi: 10.1371/journal.pmed.1004999 (PMC13485125; doi:10.1371/journal.pmed.1004999)
Supplement: Supplementary file 4 [file pmed.1004999.s006.docx]

**MS reference number: PMEDICINE-D-26-00739R2**

**Recent trends in stroke incidence and risk factor control in England (2018-2024): a record linkage population-based study**

*We thank the editor and the reviewers for their very helpful comments. We have provided below a point-by-point response to the points raised.*

===========================================
**Response to the editor**

===========================================

**1. In the Background of the Abstract and in the first section of the Author Summary, please add a sentence explaining the need for this study at this time.**

*Response: Thank you and we have revised the sentence to explain that the need for this study at this time is that we need to understand current trend. It now reads:*

*Abstract:*

*“The incidence of stroke at younger ages appears to be increasing, whilst it is falling at older ages. We aimed to determine the current trend and how it might have changed during and after the COVID-19 pandemic.”*

*Author summary:*

*“•The divergence in incidence by age predates the COVID-19 pandemic, and trends during and after the pandemic were poorly characterised.*

*•Understanding the current trend is crucial for public health and service planning.”*

**2. Please ensure that the Introduction ends with a clear description of the study question or hypothesis.**

*Response: Thank you and the last paragraph of the Introduction states the study aims:*

*“We aimed to determine the age-specific changes in stroke incidence since 2018, particularly if there was any impact related to the COVID-19 pandemic, and to assess whether changes in the management of modifiable risk factors coincided with age-specific divergence in stroke incidence in England.”*

**3. Please ensure that all abbreviations are defined at first use throughout the text.**

*Response: thank you and done.*

**4. Please confirm that all numbers presented in the abstract are present and identical to numbers presented in the main manuscript text.**

*Response: thank you and we can confirm.*

**5. Please disclose any use of AI tools and technologies to the study or article content at the end of the Methods. Please specify the name(s) and version(s) of any tool(s) used and a description of their use, including how the AI-assisted or generated content was evaluated by authors, and a clear statement of which aspects of the study, article contents, data, or supporting files were affected/generated by AI tool usage. Please also include a statement specifying who retains responsibility for the final manuscript. See our guidelines for more information:** [**https://journals.plos.org/plosmedicine/s/ethical-publishing-practice#loc-artificial-intelligence-tools-and-technologies**](https://journals.plos.org/plosmedicine/s/ethical-publishing-practice#loc-artificial-intelligence-tools-and-technologies)**.**

*Response: thank you and we can confirm that AI tools had no role and were not used in any form during the conduct of the study. The corresponding author retains responsibility for the final manuscript.*

**6. Statistical reporting:**

**- Please report statistical information as follows to improve clarity for the reader "22% (95% CI [13,28]; p</=)".**

*Response: thank you and all revised accordingly.*

**- Please separate upper and lower bounds with commas instead of hyphens as the latter can be confused with reporting of negative values.**

*Response:* *thank you and all revised accordingly.*

**- Please repeat statistical definitions (HR, CI etc.) for each set of parentheses.**

*Response:* *thank you and done.*

*** In the last sentence of the Abstract Methods and Findings section, please describe the main limitation(s) of the study's methodology.**

*Response: thank you and we added the following sentence:*

*“A key limitation was the use of routinely collected data, which lacked granularity to explore potential mechanisms.”*

*** In the abstract, please include the important dependent variables that are adjusted for in the analyses.**

*Response: apologies for not making this clearer. Standardised age-specific (age<55 versus ≥55 years) stroke incidence was calculated and compared using incidence rate ratio (IRR) and there were no adjusted analyses in the traditional regression analyses framework hence dependent variables are not relevant.*

**7. In the author summary, in the final bullet point of 'What Do These Findings Mean?', please include the main limitations of the study in non-technical language.**

*Response: thank you and we added the main limitation as suggested. It now reads:*

*“The main limitation of this study is the use of routinely collected data, which lacked granularity to allow further exploration of potential mechanistic drivers behind the apparent trends.”*

**8. FUNDING STATEMENT
The funding statement should include: specific grant numbers, initials of authors who received each award, URLs to sponsors’ websites. The funder URLs are missing from the funding statement.
It appears that one or more study authors is affiliated with one or more of the agencies that funded the study. Thus, the statement “The funders had no role in study design, data collection and analysis, decision to publish, or preparation of the manuscript” does not apply. Please revise the Financial Disclosure accordingly, as in "[Author name] is [author's role] at [funding agency]. The funders had no other role in study design…..”**

*Response: thank you and URLs added as hyperlink for the funders. We also updated the funders’ roles. It now reads:*

*“JF, JN, SL, WNW are part of the BHF Data Science Centre’s Health Data Team, who provided data curation resources and support. Otherwise the funders had no role in study design, data collection and analysis, decision to publish, or preparation of the manuscript.”*

**9. COMPETING INTERESTS STATEMENT
All authors must declare their relevant competing interests per the PLOS policy, which can be seen here:** [**https://journals.plos.org/plosmedicine/s/competing-interests**](https://journals.plos.org/plosmedicine/s/competing-interests) **For authors with ties to industry, please indicate whether any of the interests has a financial stake in the results of the current study.**

*Response: thank you and the authors have declared that no competing interest exist.*

**10. DATA AVAILABILITY**

*** PLOS defines the “minimal data set” to consist of the data set used to reach the conclusions drawn in the manuscript with related metadata and methods, and any additional data required to replicate the reported study findings in their entirety. Authors do not need to submit their entire data set, or the raw data collected during an investigation. Please submit the following data:

The values behind the means, standard deviations and other measures reported;
The values used to build graphs;
The points extracted from images for analysis.

Thank you for agreeing to make your data available. At this time, please provide the links to the national data custodians for requests to access data.**

*Response: Thank you.*

*We included the aggregated data (i.e. stroke counts) by age and year, which are the values used to calculate the relevant IRRs already in Table 1.*

*Table 1 also reported the population denominators used.*

*All risk factor data (i.e. counts by age, sex, ethnicity, time periods) presented in figure 2 and 3 are reported in full in the web-appendices.*

*As the analyses were done in a secured data environment per requirement from NHS England, no raw data were allowed to be downloaded or presented outside of the SDE.*

*We have revised the data availability statement, which was also used for similar publications in the journal (https://journals.plos.org/plosmedicine/article?id=10.1371/journal.pmed.1004485)*

*“Data availability*

*The data used in this study are available in NHS England’s Secure Data Environment (SDE) service for England, but as restrictions apply they are not publicly available (https://digital.nhs.uk/services/secure-data-environment-service). The CVD-COVID-UK/COVID-IMPACT research programme, led by the BHF Data Science Centre (https://bhfdatasciencecentre.org/), received approval to access data in NHS England’s SDE service for England from the Advisory Group for Data (AGD) (https://digital.nhs.uk/about-nhs-digital/corporate-information-and-documents/advisory-group-for-data) – formerly the Independent Group Advising on the Release of Data (IGARD) – via an application made in the Data Access Request Service (DARS) Online system (ref. DARS-NIC-381078-Y9C5K) (https://digital.nhs.uk/services/data-access-request-service-dars/dars-products-and-services). The CVD-COVID-UK/COVID-IMPACT Approvals & Oversight Board (https://bhfdatasciencecentre.org/areas/cvd-covid-uk-covid-impact/) subsequently granted approval to this project (CCU084) to access the data within NHS England’s SDE service for England. The anonymised data used in this study were made available to accredited researchers only. Those wishing to gain access to the data should contact bhfdsc@hdruk.ac.uk in the first instance and follow the application process of the relevant national data custodian (https://bhfdatasciencecentre.org/areas/cvd-covid-uk-covid-impact/). A pre-specified analysis plan is published on GitHub, along with the phenotyping and analysis code (*[*https://github.com/BHFDSC/CCU084_01*](https://github.com/BHFDSC/CCU084_01)*)”*

**11. ETHICS AND CONSENT
Please move the Ethics statement to the Methods.**

*Response: thank you and done.*

**12. Please revise for use of patient-centered language. Please note that patient-centered language is constructed with the use of post-modified nouns (e.g. 'patients with psoriasis’ (or similar) instead of ‘psoriasis patients’) putting the person first in the sentence structure.
Please do not use Asian, Black, White as a noun (see your Methods), define ‘Other’, and state whether race/ethnicity was self-reported.**

*Response:* *thank you and all checked and revised accordingly.*

**13. Please review your manuscript and edit to ensure compliance with our inclusive language requirements** [**https://journals.plos.org/plosmedicine/s/human-subjects-research#loc-categorization**](https://journals.plos.org/plosmedicine/s/human-subjects-research#loc-categorization)

*Response:* *thank you and all checked and revised accordingly.*

**14. FIGURES & TABLES
* Please ensure that where relevant figures include 95% CIs.
* When a p value is given, please specify the statistical test used to determine it in the legend.
* Please provide the unadjusted comparisons as well as the adjusted comparisons in all relevant Tables
* Please specify the variables controlled for in all relevant Tables
* Please expand the legend for Figure 1.**

*Response:* *thank you and all checked and revised accordingly. Only unadjusted comparisons were relevant for all of the analyses presented. We have now clarified that the analyses were standardised by age.*

===========================================
**Response to the reviewers**

===========================================

**Reviewer #1: The authors have responded thoroughly to the reviewers comments.
This is a clearly written, well thought through paper of trend in stroke risk in the UK using linked data with important findings**

*Response:* *thank you.*

**Reviewer #2: Thanks for the revised manuscript and responses to my original review.
I agree with the authors with the small dispersion parameter they estimated in the negbin models, that using the Poisson is reasonable. The limitations from missing data is explained well and I think that given the very small amount (~0.5%) this is very unlikely to impact the results unless there is a severe missingness mechanism.**

*Response:* *thank you.*

**Reviewer #3: Thanks for your response; my concerns have been satisfactorily addressed.**

*Response:* *thank you.*

**Reviewer #4: I thank the authors for their careful and comprehensive response to the comments raised during the previous round of major revision. Having reviewed the revised manuscript together with the point-by-point responses to my own comments, I find that the authors have engaged constructively with every point and have made changes that materially strengthen the manuscript. All eight of the comments I raised have been addressed satisfactorily.
The study remains an important and timely contribution: it provides the first age-specific data on stroke incidence in England since 2018, spanning the COVID-19 pandemic, and demonstrates the feasibility and value of linking overlapping health-system data sources for more timely disease surveillance. The conclusions are supported by the data, the methodology is sound, and the limitations are now stated transparently. I have no further substantive concerns.**

*Response:* *thank you.*
