# Supplementary material for: Time trends in stroke incidence and modifiable risk factor prevalence in England (2018–2024): A record linkage population-based study
Source: PLoS Med. 2026 Aug 7;23(8):e1004999. doi: 10.1371/journal.pmed.1004999 (PMC13485125; doi:10.1371/journal.pmed.1004999)
Supplement: Peer review history

# Editor report: Decision Letter 0

**DOI:** 10.1371/journal.pmed.1004999.r001

**Authors:** Louise Gaynor-Brook

4 Mar 2026

Dear Dr Li,

Thank you for submitting your manuscript entitled "Recent trends in stroke incidence and risk factor control in England (2018-2024): a record linkage population-based study" for consideration by PLOS Medicine.

Your manuscript has now been evaluated by the PLOS Medicine editorial staff, and I am writing to let you know that we would like to send your submission out for external peer review.

However, before we can send your manuscript to reviewers, we need you to complete your submission by providing the metadata that is required for full assessment. To this end, please login to Editorial Manager where you will find the paper in the 'Submissions Needing Revisions' folder on your homepage. Please click 'Revise Submission' from the Action Links and complete all additional questions in the submission questionnaire. Please include continuous line numbers in your manuscript (i.e. ideally not starting from 1 with each new page.)

For clinical studies, please upload a copy of your trial study protocol as a supporting information file. The study protocol should be the version submitted for approval to the institutional review board or ethics committee, should include any amendments to the study protocol, as well as the date of their approval by the institutional review or ethics committee. Please also detail any deviations from the study protocol in the Methods section of your manuscript. The editors will consider the protocol and study conduct prior to a final decision for external review.

Please re-submit your manuscript within two working days, i.e. by Mar 06 2026 11:59PM.

Login to Editorial Manager here: https://www.editorialmanager.com/pmedicine

Once your full submission is complete, your paper will undergo a series of checks in preparation for peer review. Once your manuscript has passed all checks it will be sent out for review.

Feel free to email me at lgaynor@plos.org if you have any queries relating to your submission.

Kind regards,

Louise Gaynor-Brook, MBBS PhD

Senior Editor

PLOS Medicine

# Peer review: Decision Letter 1

**DOI:** 10.1371/journal.pmed.1004999.r002

**Authors:** Louise Gaynor-Brook

14 Apr 2026

Dear Dr Li,

Many thanks for submitting your manuscript "Recent trends in stroke incidence and risk factor control in England (2018-2024): a record linkage population-based study" (PMEDICINE-D-26-00739R1) to PLOS Medicine. The paper has been reviewed by subject experts and a statistician; their comments are included below and can also be accessed here: [LINK]

After discussing the paper with the editorial team and an academic editor with relevant expertise, I'm pleased to invite you to revise the paper in response to the reviewers' comments. We plan to send the revised paper to some or all of the original reviewers, and we cannot provide any guarantees at this stage regarding publication.

In addition to these revisions, you may need to complete some formatting changes, which you will receive in a follow up email. A member of our team will be in touch with a set of requests shortly. If you do not receive a separate email within a few days, please assume that checks have been completed, and no additional changes are required.

When you upload your revision, please include a point-by-point response that addresses all of the reviewer and editorial points, indicating the changes made in the manuscript and either an excerpt of the revised text or the location (eg: page and line number) where each change can be found. Please also be sure to check the general editorial comments at the end of this letter and include these in your point-by-point response. When you resubmit your paper, please include a clean version of the paper as the main article file and a version with changes tracked as a marked-up manuscript. It may also be helpful to check the guidelines for revised papers at http://journals.plos.org/plosmedicine/s/revising-your-manuscript for any that apply to your paper.

We ask that you submit your revision by May 05 2026 11:59PM. However, if this deadline is not feasible, please contact me by email, and we can discuss a suitable alternative.

Don't hesitate to contact me directly with any questions (lgaynor@plos.org).

Best regards,

Louise

Louise Gaynor-Brook, MBBS PhD

Senior Editor

PLOS Medicine

lgaynor@plos.org

-----------------------------------------------------------

Comments from the editors:

Please provide justification regarding the advance offered by your study, compared to the existing literature.

-----------------------------------------------------------

Comments from the reviewers:

Reviewer #1: This is an interesting methodological and clinically relevant paper addressing stroke risk pre, per and post the covid pandemic. This means the time window is necessarily relatively small when looking at trends in incidence especially in subgroups but the findings are relevant to policy, service delivery as well as to populations at most risk. There are a few points the authors should consider in the paper if possible.

1. As these are either routine data sets or audits can the authors further comment on reliability of the diagnosis of first stroke (particularly in primary care records and death certificates), ethnic group and stroke severity if not admitted.

2. It would be interesting to illustrate the overlap in data sources with regard to capture and which are the most likely to be useful for further such studies.

3. Is there any further exploration of the interaction of ethnicity with deprivation indices?

4. Minor point-in abstract conclusion the authors talk about risk factor screening, yet there is no screening programme per se but case finding through optimising contacts in health settings and responses for health checks.

5. The identification of younger people from ethnic minority groups at high risk is important yet how to identify these groups and improve diagnostic and treatment rates of vascular risk factors is really unsolved and requires some discussion on approaches.

Reviewer #2: Thanks for the opportunity to read your manuscript. My role is statistical reviewer, so I have focused on the design, data, and analysis that are presented. I have put general comments first, followed by questions relevant to a specific section of the manuscript (with a page/paragraph reference).

This manuscript describes incidence of stroke and prevalence of risk factors for stroke in England from 2018 and 2024, with a focus on younger age groups. The study uses linked NHS health data that includes GP visits, hospital admissions, death records, and stroke registry data. Events were included if they occurred between April 2018 and March 2028, and there was no previous stroke recorded in the data. Information on the stroke, as well as patient information, comorbidities, and medications used was also derived from linked data. Fatal stroke was defined as any death within 30 days of a recorded stroke. Risk factors were considered 'prevalent' if they were recorded at least 30 days before the stroke. The study period was divided into pre-COVID, COVID, and post-COVID. Incidence rates were standardised to the 2011 population of England and Wales. Incident rate ratios are reported relative to the 'COVID' study period, and are stratified by age, sex, ethnicity, area measure of deprivation, regions, stroke sub-types and data source. Poisson regression was used to estimate IRRs.

There was a higher incidence rate of stroke in 2022-2024 compared to 2020-2022, this was consistent across nearly all of the stratification groups in both of the age-groups.

The rationale for the time-periods is explained well - but would it also be possible to see overall (and by the two age-groups) incidence rates per year across the study period?

P4, Paragraph 2. What is the extent of the datasets for determining strokes that occur before 2018? i.e. what is the look-back period?

P5, Paragraph 3. Were the Poisson regression models checked for overdispersion?

P5, Paragraph 7. I tried to access the repository - but this didn't load. I also couldn't find it listed in the BHFDSC github page.

P16, Table 1. Is 'per 1,000' per 1,000 person-years?

P20, Figure 2. This is a nice figure. Optionally, you could add an explainer to the x-axis saying 'more common in 2020-2022' and 'more common in 2018-2020'

Reviewer #3: This article describes trends in English stroke incidence and pharmacological risk factor management, stratified by pre/ during/ post pandemic periods. The paper was clear and well-written and presented. My major query is the rationale for the <55 years age cut-off, which many of the analyses were based on, but didn't seem to be borne out by the descriptive data as far as I could see. Some other minor comments/ suggestions are also outlined below.

Introduction

Given you have published previously on age-divergent trends in stroke incidence, in the first and last paras of this section the paper would be better framed as an investigation of peri-pandemic trends and risk factors. Or are you using better data sources? If so, you should state this.

You postulate decreased insulin use in people with type 1 diabetes during the pandemic as an explanation for higher stroke incidence in younger people - this doesn't sound likely to me. Were there any data on age trends in statin/ antihypertensive prescriptions over the pre/ during/ post pandemic periods in the paper cited by Dale et al?

Also you haven't mentioned a key driver of CVD risk in younger people - rising obesity levels - shown to be worse for young than old people during the pandemic (https://pubmed.ncbi.nlm.nih.gov/38913709/).

Did you also consider competing risks here (and in the analysis)? COVID-19 had far higher mortality in older people.

Methods

The text would be clearer if the covariates (sex, ethnicity etc) were described in a separate para to the stroke ascertainment.

How did you ascertain presence of risk factors and from which sources?

How was the overlap dealt with between prevalent/ incident/ missed versus treated groups (see comment below)?

Results

It would be good to see a table of baseline characteristics. Ideally by pandemic period and presence/ absence of stroke so the data structure can be understood at a glance, including missingness for ethnicity etc.

Why did you choose to stratify age at 55 years? It seems slightly counter-intuitive given that patterns of increasing incidence seems to hold until up to 75 yrs, according to table 1. But perhaps there's a clinical justification for this threshold.

For clarity, can you expand "ASI" in the legend of table 2? And reiterate what the RTTRs are comparing in the text of the results section?

In web appendix 8, the overlap between prevalent/ incident/ missed versus treated groups is confusing. For example, in 2018-2020, if 73.5% had prevalent hypertension but only 35.3% (presumably nested within that 73.5%) were treated, were the remaining 38.2% classified as missed? Yet only 7.6% are missed in the table. Or were some of them below treatment thresholds? Clearly people with hypertension requiring treatment who aren't on it will be at higher risk, so it's important to show that in the table somehow. See above comment in the methods section.

Also did you have any data on risk factor control?

Did you consider adjusting you IRR for the age differences in stroke rate increases for the risk factors? That could enable a firmer conclusion as to the cause of the age/ pandemic period divergences.

Discussion

Here again the spiraling rates of obesity/ type 2 diabetes are only mentioned cursorily, but are likely to be a key driver of strokes in younger people.

You do not mention missing data in your limitations para. Presumably there was missingness for ethnicity, and some of the risk factor data in the SDE (or did you ascertain that from other sources?).

Reviewer #4: This manuscript reports a large, methodologically ambitious, and policy-relevant population-based study using linked national data sources to examine age-specific trends in stroke incidence before, during, and after the COVID-19 pandemic. The study addresses an important and timely question and has the potential to make a substantial contribution to the literature. The observation of a persisting increase in stroke incidence among adults aged <55 years, particularly among ethnic minority groups, has important implications for both clinical practice and health policy and is appropriately highlighted by the authors.

The manuscript is generally well written, the analyses are coherent, and the conclusions are largely supported by the presented data.

I would recommend several methodological clarifications, refinements, and points to discuss. Addressing the comments outlined below would, in my view, strengthen the scientific rigour and translational relevance of this work.

1.Case ascertainment and pandemic related issues

The use of multiple overlapping data sources is a major strength; however, the manuscript would benefit from clearer reassurance that changes in healthcare utilisation during the pandemic did not differentially affect stroke ascertainment by age group.

a.Could you provide any details on whether the completeness and timeliness of stroke recording (especially in primary care and audit data) varied across the three study periods?

b.Younger adults may have been more likely to delay or avoid care during the pandemic; could you discuss how this might bias estimates?

2.Choice of age threshold (<55 vs ≥55 years)

The dichotomisation at age 55 seems reasonable and aligns with some prior literature, but it remains somewhat arbitrary. Please justify this cut point.

3.Interpretation of increasing post pandemic incidence

The increase in stroke incidence at age ≥55 years in the post pandemic period deserves more careful interpretation. Could this reflect delayed presentations or diagnostic backlog rather than a true rise in incidence?

4.Undiagnosed risk factors

The finding of increased "undiagnosed" hypertension and diabetes in younger adults is important but requires clearer methodological explanation. How exactly were "undiagnosed" cases defined? Is it possible that reduced primary care contact led to delayed coding rather than truly missed detection?

5.Stroke subtype analyses

Specify whether ANY trends differed meaningfully between ischaemic and haemorrhagic stroke.

6.Terminology and clarity

a.I would suggest providing an explanation of the Relative Temporal Rate Ratio (RTTR) in the Methods, as this metric may be unfamiliar to some readers.

b.The sentence "Research into the best method to reduce missed opportunities for primary prevention, especially for younger patients" in the Discussion sounds unclear to me.

7.References

Please use full metrics for Refs #15, #19, #26, #27:

#15: PLoS Med. 2024;21(11):e1004485

#19: Eur Heart J. 2025;46(42):4359-4378

#26: Nat Commun. 2025;16(1):6059.

#27: EClinicalMedicine. 2023;61:102077.

Any attachments provided with reviews can be seen via the following link: [LINK]

--------------------------------------------------------- ---

General editorial requests:

(Note: not all will apply to your paper, but please check each item carefully)

*We ask every co-author listed on the manuscript to fill in a contributing author statement, making sure to declare all competing interests. If any of the co-authors have not filled in the statement, we will remind them to do so when the paper is revised. If all statements are not completed in a timely fashion this could hold up the re-review process. If new competing interests are declared later in the revision process, this may also hold up the submission. Should there be a problem getting one of your co-authors to fill in a statement we will be in contact. Please do not add or remove authors without first discussing this with the handling editor. You can see our competing interests policy here: http://journals.plos.org/plosmedicine/s/competing-interests.

*Please upload any figures associated with your paper as individual TIF or EPS files with 300dpi resolution at resubmission; please read our figure guidelines for more information on our requirements: http://journals.plos.org/plosmedicine/s/figures. While revising your submission, we strongly recommend that you use PLOS's NAAS tool (https://ngplosjournals.pagemajik.ai/artanalysis) to test your figure files. NAAS can convert your figure files to the TIFF file type and meet basic requirements (such as print size, resolution), or provide you with a report on issues that do not meet our requirements and that NAAS cannot fix.

After uploading your figures to PLOS's NAAS tool - https://ngplosjournals.pagemajik.ai/artanalysis, NAAS will process the files provided and display the results in the "Uploaded Files" section of the page as the processing is complete.

If the uploaded figures meet our requirements (or NAAS is able to fix the files to meet our requirements), the figure will be marked as "fixed" above. If NAAS is unable to fix the files, a red "failed" label will appear above.

When NAAS has confirmed that the figure files meet our requirements, please download the file via the download option, and include these NAAS processed figure files when submitting your revised manuscript.

*Please ensure that the paper adheres to the PLOS Data Availability Policy (see http://journals.plos.org/plosmedicine/s/data-availability), which requires that all data underlying the study's findings be provided in a repository or as Supporting Information. For data residing with a third party, authors are required to provide instructions with contact information (web or email address) for obtaining the data. Please note that a study author cannot be the contact person for the data. PLOS journals do not allow statements supported by "data not shown" or "unpublished results." For such statements, authors must provide supporting data or cite public sources that include it.

*We expect all researchers with submissions to PLOS in which author-generated code underpins the findings in the manuscript to make all author-generated code available without restrictions upon publication of the work. In cases where code is central to the manuscript, we may require the code to be made available as a condition of publication. Authors are responsible for ensuring that the code is reusable and well documented. Please make any custom code available, either as part of your data deposition or as a supplementary file. Please add a sentence to your data availability statement regarding any code used in the study, e.g. "The code used in the analysis is available from Github [URL] and archived in Zenodo [DOI link]" Please review our guidelines at https://journals.plos.org/plosmedicine/s/materials-software-and-code-sharing and ensure that your code is shared in a way that follows best practice and facilitates reproducibility and reuse. Because Github depositions can be readily changed or deleted, we encourage you to make a permanent DOI'd copy (e.g. in Zenodo) and provide the URL.

*Please ensure that the study is reported according to the RECORD guideline and include the completed checklist as Supporting Information.

FORMATTING - GENERAL

*Abstract: Please structure your abstract using the PLOS Medicine headings (Background, Methods and Findings, Conclusions). Please combine the Methods and Findings sections into one section.

*At this stage, we ask that you include a short, non-technical Author Summary of your research to make findings accessible to a wide audience that includes both scientists and non-scientists. The Author Summary should immediately follow the Abstract in your revised manuscript. This text is subject to editorial change and should be distinct from the scientific abstract. Ideally each sub-heading should contain 2-3 single sentence, concise bullet points containing the most salient points from your study. In the final bullet point of 'What Do These Findings Mean?', please include the main limitations of the study in non-technical language. Please see our author guidelines for more information: https://journals.plos.org/plosmedicine/s/revising-your-manuscript#loc-author-summary.

*Please express the main results with 95% CIs as well as p values. When reporting p values please report as p<0.001 and where higher as the exact p value p=0.002, for example. Throughout, suggest reporting statistical information as follows to improve clarity for the reader "22% (95% CI [13%,28%]; p</=)". Please be sure to define all numerical values at first use.

*Please include page numbers and line numbers in the manuscript file. Use continuous line numbers (do not restart the numbering on each page).

*Please cite the reference numbers in square brackets. Citations should precede punctuation.

FIGURES AND TABLES

*Please provide titles and legends for all figures and tables (including those in Supporting Information files).

*Please define all abbreviations used in each figure/table (including those in Supporting Information files).

*Please consider avoiding the use of red and green in order to make your figure more accessible to those with color blindness.

SUPPLEMENTARY MATERIAL

*Please note that supplementary material will be posted as supplied by the authors. Therefore, please amend it according to the relevant comments outlined here.

*Please cite your Supporting Information as outlined here: https://journals.plos.org/plosmedicine/s/supporting-information

REFERENCES

*PLOS uses the numbered citation (citation-sequence) method and first six authors, et al.

*Please ensure that journal name abbreviations match those found in the National Center for Biotechnology Information (NCBI) databases (http://www.ncbi.nlm.nih.gov/nlmcatalog/journals), and are appropriately formatted and capitalised.

*Where website addresses are cited, please include the complete URL and specify the date of access (e.g. [accessed: 12/06/2023]).

*Please also see https://journals.plos.org/plosmedicine/s/submission-guidelines#loc-references for further details on reference formatting.

STUDY TYPE-SPECIFIC REQUESTS - OBSERVATIONAL STUDIES

*Abstract: Please include the study design, population and setting, number of participants, years during which the study took place (enrollment and follow up), length of follow up, and main outcome measures.

*Please ensure that the study is reported according to the RECORD guideline (available from https://www.record-statement.org) and include the completed checklist as Supporting Information. Please add the following statement, or similar, to the Methods: "This study is reported as per the Reporting of Studies Conducted using Observational Routinely-Collected Data (RECORD) guideline (S1 Checklist)." When completing the checklist, please use section and paragraph numbers, rather than page numbers.

*For all observational studies, in the manuscript text, please indicate: (1) the specific hypotheses you intended to test, (2) the analytical methods by which you planned to test them, (3) the analyses you actually performed, and (4) when reported analyses differ from those that were planned, transparent explanations for differences that affect the reliability of the study's results. If a reported analysis was performed based on an interesting but unanticipated pattern in the data, please be clear that the analysis was data driven.

*Please state in the Methods section whether the study had a prospective protocol or analysis plan. If a prospective analysis plan (from your funding proposal, IRB or other ethics committee submission, study protocol, or other planning document written before analyzing the data) was used in designing the study, please include the relevant document(s) with your revised manuscript as a Supporting Information file to be published alongside your study and cite it in the Methods section. A legend for this file should be included at the end of your manuscript. If no such document exists, please make sure that the Methods section transparently describes when analyses were planned, and when/why any data-driven changes to analyses took place. Changes in the analysis, including those made in response to peer review comments, should be identified as such in the Methods section of the paper, with rationale.

# Author comment: Author response to Decision Letter 2

**DOI:** 10.1371/journal.pmed.1004999.r003

21 May 2026

Attachment Submitted filename: R1_response_time trends stroke incidence.docx

# Peer review: Decision Letter 2

**DOI:** 10.1371/journal.pmed.1004999.r004

**Authors:** Louise Gaynor-Brook

13 Jul 2026

Dear Dr. Li,

Thank you very much for re-submitting your manuscript "Recent trends in stroke incidence and risk factor control in England (2018-2024): a record linkage population-based study" (PMEDICINE-D-26-00739R2) for review by PLOS Medicine. I am writing on behalf of my colleague, Dr Louise Gaynor-Brook.

I have discussed the paper with my colleagues and the academic editor and it was also seen again by 4 reviewers. I am pleased to say that provided the remaining editorial and production issues are dealt with we are planning to accept the paper for publication in the journal.

The remaining issues that need to be addressed are listed at the end of this email. Any accompanying reviewer attachments can be seen via the link below. Please take these into account before resubmitting your manuscript:

[LINK]

***Please note while forming your response, if your article is accepted, you may have the opportunity to make the peer review history publicly available. The record will include editor decision letters (with reviews) and your responses to reviewer comments. If eligible, we will contact you to opt in or out.***

In revising the manuscript for further consideration here, please ensure you address the specific points made by each reviewer and the editors. In your rebuttal letter you should indicate your response to the reviewers' and editors' comments and the changes you have made in the manuscript. Please submit a clean version of the paper as the main article file. A version with changes marked must also be uploaded as a marked up manuscript file.

Please also check the guidelines for revised papers at http://journals.plos.org/plosmedicine/s/revising-your-manuscript for any that apply to your paper. If you haven't already, we ask that you provide a short, non-technical Author Summary of your research to make findings accessible to a wide audience that includes both scientists and non-scientists. The Author Summary should immediately follow the Abstract in your revised manuscript. This text is subject to editorial change and should be distinct from the scientific abstract.

In addition to these revisions, you may need to complete some formatting changes, which you will receive in a follow up email. A member of our team will be in touch with a set of requests shortly. If you do not receive a separate email within a few days, please assume that checks have been completed, and no additional changes are required.

We expect to receive your revised manuscript within 1 week. Please email us (plosmedicine@plos.org) if you have any questions or concerns.

We ask every co-author listed on the manuscript to fill in a contributing author statement. If any of the co-authors have not filled in the statement, we will remind them to do so when the paper is revised. If all statements are not completed in a timely fashion this could hold up the re-review process. Should there be a problem getting one of your co-authors to fill in a statement we will be in contact. YOU MUST NOT ADD OR REMOVE AUTHORS UNLESS YOU HAVE ALERTED THE EDITOR HANDLING THE MANUSCRIPT TO THE CHANGE AND THEY SPECIFICALLY HAVE AGREED TO IT.

Please ensure that the paper adheres to the PLOS Data Availability Policy (see http://journals.plos.org/plosmedicine/s/data-availability), which requires that all data underlying the study's findings be provided in a repository or as Supporting Information. For data residing with a third party, authors are required to provide instructions with contact information for obtaining the data. PLOS journals do not allow statements supported by "data not shown" or "unpublished results." For such statements, authors must provide supporting data or cite public sources that include it.

To enhance the reproducibility of your results, we recommend that you deposit your laboratory protocols in protocols.io, where a protocol can be assigned its own identifier (DOI) such that it can be cited independently in the future. Additionally, PLOS ONE offers an option to publish peer-reviewed clinical study protocols. Read more information on sharing protocols at https://plos.org/protocols?utm_medium=editorial-email&utm_source=authorletters&utm_campaign=protocols

Please review your reference list to ensure that it is complete and correct. If you have cited papers that have been retracted, please include the rationale for doing so in the manuscript text, or remove these references and replace them with relevant current references. Any changes to the reference list should be mentioned in the rebuttal letter that accompanies your revised manuscript.

Please note, when your manuscript is accepted, an uncorrected proof of your manuscript will be published online ahead of the final version, unless you've already opted out via the online submission form. If, for any reason, you do not want an earlier version of your manuscript published online or are unsure if you have already indicated as such, please let the journal staff know immediately at plosmedicine@plos.org.

If you have any questions in the meantime, please contact me or the journal staff on plosmedicine@plos.org.

We look forward to receiving the revised manuscript by Jul 20 2026 11:59PM.

Sincerely,

Alison Farrell, PhD

Senior Editor

PLOS Medicine

plosmedicine.org

------------------------------------------------------------

Requests from Editors:

GENERAL EDITORIAL REQUESTS

* In the Background of the Abstract and in the first section of the Author Summary, please add a sentence explaining the need for this study at this time.

* Please ensure that the Introduction ends with a clear description of the study question or hypothesis.

* Please ensure that all abbreviations are defined at first use throughout the text.

* Please confirm that all numbers presented in the abstract are present and identical to numbers presented in the main manuscript text.

GENERAL

* Please disclose any use of AI tools and technologies to the study or article content at the end of the Methods. Please specify the name(s) and version(s) of any tool(s) used and a description of their use, including how the AI-assisted or generated content was evaluated by authors, and a clear statement of which aspects of the study, article contents, data, or supporting files were affected/generated by AI tool usage. Please also include a statement specifying who retains responsibility for the final manuscript. See our guidelines for more information: https://journals.plos.org/plosmedicine/s/ethical-publishing-practice#loc-artificial-intelligence-tools-and-technologies.

* Statistical reporting: Please revise throughout the manuscript, including tables and figures.

- Please report statistical information as follows to improve clarity for the reader "22% (95% CI [13,28]; p</=)".

- Please separate upper and lower bounds with commas instead of hyphens as the latter can be confused with reporting of negative values.

- Please repeat statistical definitions (HR, CI etc.) for each set of parentheses.

* In the last sentence of the Abstract Methods and Findings section, please describe the main limitation(s) of the study's methodology.

* In the abstract, please include the important dependent variables that are adjusted for in the analyses.

* In the author summary, in the final bullet point of 'What Do These Findings Mean?', please include the main limitations of the study in non-technical language.

FUNDING STATEMENT

* The funding statement should include: specific grant numbers, initials of authors who received each award, URLs to sponsors’ websites. The funder URLs are missing from the funding statement.

* It appears that one or more study authors is affiliated with one or more of the agencies that funded the study. Thus, the statement “The funders had no role in study design, data collection and analysis, decision to publish, or preparation of the manuscript” does not apply. Please revise the Financial Disclosure accordingly, as in "[Author name] is [author's role] at [funding agency]. The funders had no other role in study design…..”

COMPETING INTERESTS STATEMENT

* All authors must declare their relevant competing interests per the PLOS policy, which can be seen here: https://journals.plos.org/plosmedicine/s/competing-interests For authors with ties to industry, please indicate whether any of the interests has a financial stake in the results of the current study.

DATA AVAILABILITY

* PLOS defines the “minimal data set” to consist of the data set used to reach the conclusions drawn in the manuscript with related metadata and methods, and any additional data required to replicate the reported study findings in their entirety. Authors do not need to submit their entire data set, or the raw data collected during an investigation. Please submit the following data:

The values behind the means, standard deviations and other measures reported;

The values used to build graphs;

The points extracted from images for analysis.

* Thank you for agreeing to make your data available. At this time, please provide the links to the national data custodians for requests to access data.

ETHICS AND CONSENT

Please move the Ethics statement to the Methods.

* Please revise for use of patient-centered language. Please note that patient-centered language is constructed with the use of post-modified nouns (e.g. 'patients with psoriasis’ (or similar) instead of ‘psoriasis patients’) putting the person first in the sentence structure.

** Please do not use Asian, Black, White as a noun (see your Methods), define ‘Other’, and state whether race/ethnicity was self-reported.

* Please review your manuscript and edit to ensure compliance with our inclusive language requirements https://journals.plos.org/plosmedicine/s/human-subjects-research#loc-categorization

FIGURES & TABLES

* Please ensure that where relevant figures include 95% CIs.

* When a p value is given, please specify the statistical test used to determine it in the legend.

* Please provide the unadjusted comparisons as well as the adjusted comparisons in all relevant Tables

* Please specify the variables controlled for in all relevant Tables

* Please expand the legend for Figure 1.

Comments from Reviewers:

Reviewer #1: The authors have responded thoroughly to the reviewers comments.

This is a clearly written, well thought through paper of trend in stroke risk in the UK using linked data with important findings

Reviewer #2: Thanks for the revised manuscript and responses to my original review.

I agree with the authors with the small dispersion parameter they estimated in the negbin models, that using the Poisson is reasonable. The limitations from missing data is explained well and I think that given the very small amount (~0.5%) this is very unlikely to impact the results unless there is a severe missingness mechanism.

Reviewer #3: Thanks for your response; my concerns have been satisfactorily addressed.

Reviewer #4: I thank the authors for their careful and comprehensive response to the comments raised during the previous round of major revision. Having reviewed the revised manuscript together with the point-by-point responses to my own comments, I find that the authors have engaged constructively with every point and have made changes that materially strengthen the manuscript. All eight of the comments I raised have been addressed satisfactorily.

The study remains an important and timely contribution: it provides the first age-specific data on stroke incidence in England since 2018, spanning the COVID-19 pandemic, and demonstrates the feasibility and value of linking overlapping health-system data sources for more timely disease surveillance. The conclusions are supported by the data, the methodology is sound, and the limitations are now stated transparently. I have no further substantive concerns.

Any attachments provided with reviews can be seen via the following link:

[LINK]

# Author comment: Author response to Decision Letter 3

**DOI:** 10.1371/journal.pmed.1004999.r005

21 Jul 2026

Attachment Submitted filename: R2_response_time trends stroke incidence.docx

# Editor report: Decision Letter 3

**DOI:** 10.1371/journal.pmed.1004999.r006

**Authors:** Alison Farrell

24 Jul 2026

Dear Dr Li,

On behalf of my colleagues and the Academic Editor, Joshua Willey I am pleased to inform you that we have agreed to publish your manuscript "Recent trends in stroke incidence and risk factor control in England (2018-2024): a record linkage population-based study" (PMEDICINE-D-26-00739R3) in PLOS Medicine.

Before your manuscript can be formally accepted you will need to complete some formatting changes, which you will receive in a follow up email. Please be aware that it may take several days for you to receive this email; during this time no action is required by you. Once you have received these formatting requests, please note that your manuscript will not be scheduled for publication until you have made the required changes.

We further ask you to make the following revisions to the manuscript:

Please revise the title to : "Time trends in stroke incidence and modifiable risk factor prevalence in England (2018-2024): A record linkage population-based study"

The funder URLs are contained as hyperlinks in the manuscript. Please provide spelled out versions for the manuscript metadata.

The Github entry requires a DOI. Please refer to email.

The sentence " Otherwise, the funders had no role in study design, data collection and analysis, decision to publish, or preparation of the manuscript" does not seem appropriate in view of the fact that several co-authors are employees of the funder and participated in drafting the manuscript. Please delete it.

In the Statistical Analysis subsection of the Methods, please add the rationale for the definition of the post pandemic time period (i.e. aligned with the UK switching from pandemic to epidemic response?), given that this differes from the WHO definition of post-pandemic.

In the meantime, please log into Editorial Manager at http://www.editorialmanager.com/pmedicine/, click the "Update My Information" link at the top of the page, and update your user information to ensure an efficient production process.

PRESS

We frequently collaborate with press offices. If your institution or institutions have a press office, please notify them about your upcoming paper at this point, to enable them to help maximise its impact. If the press office is planning to promote your findings, we would be grateful if they could coordinate with medicinepress@plos.org. If you have not yet opted out of the early version process, we ask that you notify us immediately of any press plans so that we may do so on your behalf.

We also ask that you take this opportunity to read our Embargo Policy regarding the discussion, promotion and media coverage of work that is yet to be published by PLOS. As your manuscript is not yet published, it is bound by the conditions of our Embargo Policy. Please be aware that this policy is in place both to ensure that any press coverage of your article is fully substantiated and to provide a direct link between such coverage and the published work. For full details of our Embargo Policy, please visit http://www.plos.org/about/media-inquiries/embargo-policy/.

To enhance the reproducibility of your results, we recommend that you deposit your laboratory protocols in protocols.io, where a protocol can be assigned its own identifier (DOI) such that it can be cited independently in the future. Additionally, PLOS ONE offers an option to publish peer-reviewed clinical study protocols. Read more information on sharing protocols at https://plos.org/protocols?utm_medium=editorial-email&utm_source=authorletters&utm_campaign=protocols

Thank you again for submitting to PLOS Medicine. We look forward to publishing your paper.

Sincerely,

Alison Farrell, PhD

Senior Editor

PLOS Medicine
